# Supplementary material for: SARS-CoV-2 variants of concern in children and adolescents with COVID-19: a systematic review
Source: BMJ Open. 2023 Oct 9;13(10):e072280. doi: 10.1136/bmjopen-2023-072280 (PMC10565293; doi:10.1136/bmjopen-2023-072280)
Supplement: Supplementary data [file bmjopen-2023-072280supp001.pdf]

Search strategy

1. COAP database (https://zika.ispm.unibe.ch/assets/data/pub/search_beta/)
searched from: 1 January 2020 to 1 March 2022 - the database was not updated after 28 February 2022
search query: child* AND variant*
search field: Title OR Abstract

2. PubMed
   a) Search strategy from 1 August 2020 to 7 March 2022.
   Publication date filter:
   1 August 2020 to 30 January 2022: none (publications from before 1 August 2020 were manually removed afterwards)
   31 January to 7 March 2022: 2022/1/20- 2022/3/7

   Search query:
   “child*"[Title/Abstract] OR "neonat*"[Title/Abstract] OR "adolesc*"[Title/Abstract] OR "infan*"[Title/Abstract] OR "teen*"[Title/Abstract] OR "pediatr*"[Title/Abstract] OR "paediatr*"[Title/Abstract] OR ("educational status"[MeSH Terms] OR ("educational"[Title/Abstract] AND "status"[Title/Abstract]) OR "educational status"[Title/Abstract] OR "schooling"[Title/Abstract] OR "education"[MeSH Terms] OR "education"[Title/Abstract] OR "school s"[Title/Abstract] OR "schooled"[Title/Abstract] OR "schools"[MeSH Terms] OR "schools"[Title/Abstract] OR "school"[Title/Abstract]) OR ("nurseries, infant"[MeSH Terms] OR ("nurseries"[Title/Abstract] AND "infant"[Title/Abstract]) OR "infant nurseries"[Title/Abstract] OR "nurseries"[Title/Abstract] OR "nursery"[Title/Abstract]) OR ("infant, newborn"[MeSH Terms] OR ("infant"[Title/Abstract] AND "newborn"[Title/Abstract]) OR "newborn infant"[Title/Abstract] OR "newborn"[Title/Abstract] OR "newborns"[Title/Abstract] OR "newborns"[Title/Abstract]) OR "toddler*"[Title/Abstract] OR ("minority groups"[MeSH Terms] OR ("minority"[Title/Abstract] AND "groups"[Title/Abstract]) OR "minority groups"[Title/Abstract] OR "minorities"[Title/Abstract] OR "minority"[Title/Abstract] OR "minority s"[Title/Abstract] OR "minors"[MeSH Terms] OR "minors"[Title/Abstract] OR "minor"[Title/Abstract]) AND “COVID-19”[tiab] OR 2019-novel-cov*[tiab] OR coronavirus[tiab] OR coronavirusdisease-19*[tiab] OR corona-virus-disease-19*[tiab] OR covid-19*[tiab] OR covid19*[tiab] OR new-coronavirus[tiab] OR new-corona-virus[tiab] OR novelcoronavirus[tiab] OR novel-corona-virus[tiab] OR sars-2*[tiab] OR sars2*[tiab] OR sars-cov-19*[tiab] OR sarscov19*[tiab] OR sars-cov-2*[tiab] OR sars-cov2*[tiab] OR sarscov2*[tiab] OR sarscov-2*[tiab] AND "VOC"[All Fields] OR "variant of concern"[All Fields] OR "alpha variant"[All Fields] OR "beta variant"[All Fields] OR "gamma variant"[All Fields] OR "delta variant"[All Fields] OR “omicron variant”[All Fields] OR "variant of interest"[All Fields]

   b) Search strategy from 8 March 2022 to 9 May 2022
   Publication date filter: 2022/3/1- 2022/5/9

Search query:
(("Alpha"[Title/Abstract] OR "Beta"[Title/Abstract] OR "Gamma"[Title/Abstract] OR "Omicron"[Title/Abstract] OR "Delta"[Title/Abstract]) AND "variant"[Title/Abstract]) OR "B.1.1.529"[Title/Abstract] OR "B.1.617.2"[Title/Abstract] OR "B.1.1.7"[Title/Abstract] OR "B.1.351"[Title/Abstract] AND ((((((((child*) OR (neonat*)) OR (adolesc*)) OR (infant*)) OR (teen*)) OR (pediatr*)) OR (paediatr*)) OR (toddler*)) OR (newborn*)

The date filters applied in the last 2 searches overlapped on purpose to reduce the risk of us missing any publications due to their publication date.
