# Supplementary material for: SARS-CoV-2 variants of concern in children and adolescents with COVID-19: a systematic review
Source: BMJ Open. 2023 Oct 9;13(10):e072280. doi: 10.1136/bmjopen-2023-072280 (PMC10565293; doi:10.1136/bmjopen-2023-072280)
Supplement: Supplementary data [file bmjopen-2023-072280supp005.pdf]

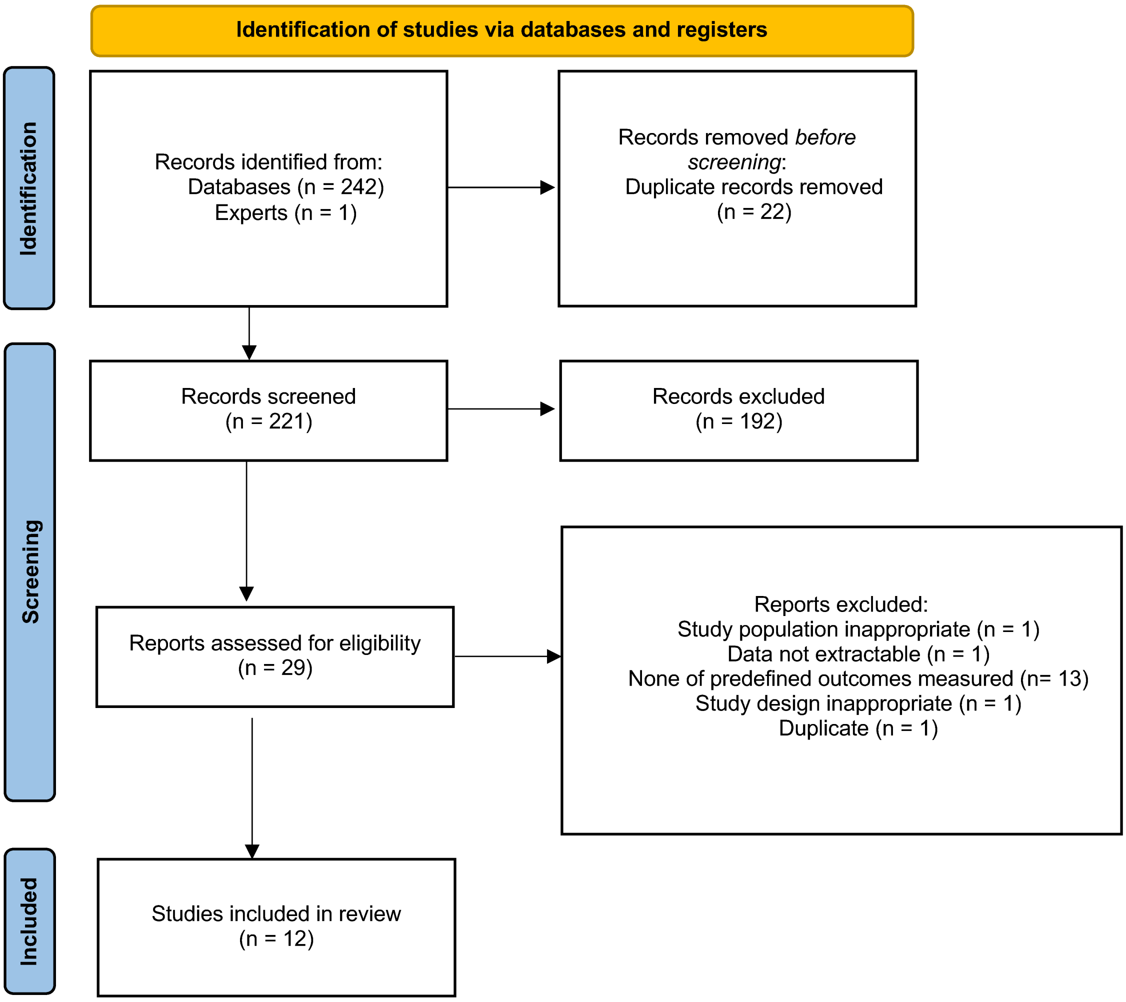

Supplementary figure 4: Flow chart – fourth search (07.03.2022-09.05.2022)
Of the 243 studies found via the database search (incl. 1 study recommended by experts) 12 studies were found to be eligible.

From: Page MJ, McKenzie JE, Bossuyt PM, Boutron I, Hoffmann TC, Mulrow CD, et al. The PRISMA 2020 statement: an updated guideline for reporting systematic reviews. BMJ 2021;372:n71. doi: 10.1136/bmj.n71

For more information, visit: http://www.prisma-statement.org/
