# Supplementary material for: SARS-CoV-2 variants of concern in children and adolescents with COVID-19: a systematic review
Source: BMJ Open. 2023 Oct 9;13(10):e072280. doi: 10.1136/bmjopen-2023-072280 (PMC10565293; doi:10.1136/bmjopen-2023-072280)
Supplement: Supplementary data [file bmjopen-2023-072280supp007.pdf]

Supplementary table 2: Risk of bias assessment - Child/adolescent symptoms/severity of disease when infected with a VOC

Cohort studies[1]	Exposed and non-exposed cohorts from the same population	Confidence in exposure assessment	Confidence that outcome of interest was not present at start of study	Matching of exposed and unexposed for all variables associated with outcome of interest or adjustment of prognostic variables by statistical analysis	Confidence in assessment of presence/absence of prognostic factors	Confidence in outcome assessment	Adequate follow-up of cohorts	Similar co-interventions between groups
Ulyte et al. [16]	definitely yes	probably no	definitely no	probably no	probably yes	probably no	definitely no	definitely yes
Nakel et al. [19]	probably yes	definitely yes	probably yes	definitely no	definitely no	probably no	definitely no	probably yes
Somekh et al. [33]	probably yes	definitely yes	probably yes	probably no	probably no	definitely yes	probably yes	probably no
Swann et al. [20]	definitely yes	definitely yes	probably no	probably yes	definitely yes	definitely yes	probably yes	probably yes
Edward et al. [28]	probably yes	definitely yes	definitely yes	probably yes	probably no	definitely yes	probably yes	probably no
Celebi et al. [41]	probably yes	probably yes	definitely yes	definitely no	definitely no	probably yes	probably no	definitely no
Stewart et al. [21]	probably yes	definitely yes	probably yes	probably no	probably no	definitely yes	definitely yes	probably no
Brookman et al. [34]	probably yes	definitely yes	definitely yes	definitely no	definitely no	probably yes	probably no	probably yes
Li et al. [22]	definitely yes	definitely yes	probably yes	definitely yes	probably no	probably yes	definitely yes	definitely yes
Shoji et al. [39]	definitely yes	definitely yes	definitely yes	probably no	probably yes	definitely yes	probably yes	probably no
Fisman et al. [38]	probably yes	definitely yes	definitely yes	probably yes	probably yes	definitely yes	probably yes	probably yes
Ryu et al. [24]	probably yes	definitely yes	probably yes	definitely no	definitely no	probably yes	probably no	probably no
Martin et al. [40]	probably yes	probably yes	definitely yes	probably no	probably no	definitely yes	probably no	definitely no
Molteni et al. [26]	definitely yes	probably yes	probably yes	probably yes	probably no	probably yes	probably yes	probably no
Murillo-Zamora et al. [37]	probably yes	definitely yes	definitely yes	definitely no	probably no	definitely yes	probably no	probably no
Butt et al. [25]	probably yes	probably yes	probably yes	probably yes	probably no	definitely yes	probably no	probably no
Oliveira et al. [35]	probably yes	definitely yes	definitely yes	definitely no	definitely no	definitely yes	probably no	probably no
Cloete et al. [29]	probably yes	probably yes	probably yes	probably yes	probably yes	probably yes	definitely yes	definitely yes
Wang et al. [42]	probably yes	probably yes	probably yes	probably yes	probably yes	definitely yes	probably no	probably no
Wang et al. [43]	probably yes	probably yes	probably yes	probably no	probably no	definitely yes	probably no	probably no

[1] CLARITY Group at McMaster University. Tool to Assess Risk of Bias in Cohort Studies [Available from: https://www.evidencepartners.com/wp-content/uploads/2021/03/Tool-to-Assess-Risk-of-Bias-in-Cohort-Studies-DistillerSR.pdf.

Marks et al. [44]	definitely yes	definitely yes	definitely yes	definitely no	definitely no	probably no	definitely yes	probably no
Butt et al. [31]	probably yes	definitely yes	probably yes	probably yes	probably yes	definitely yes	definitely no	probably yes
Edward et al. [36]	probably yes	definitely yes	definitely yes	probably yes	probably no	definitely yes	probably yes	probably no
Kildegaard et al. [27]	probably yes	probably yes	probably yes	definitely no	probably no	definitely yes	definitely yes	probably yes
Fowlkes et al. [32]	definitely yes	probably yes	probably yes	probably no	definitely no	probably yes	probably yes	probably yes
Hao et al. [23]	probably yes	probably yes	probably yes	probably no	definitely no	probably yes	probably yes	probably yes
Brewster et al. [14]	definitely yes	definitely yes	definitely yes	definitely no	definitely yes	definitely yes	probably yes	probably no
Waltenburg et al. [18]	probably yes	probably yes	probably yes	probably no	probably no	probably yes	definitely no	probably yes
Marks et al. [47]	probably yes	probably no	probably yes	probably no	probably no	definitely yes	probably yes	probably yes
Nygaard et al. [48]	probably yes	probably yes	definitely yes	probably no	definitely no	definitely yes	definitely yes	probably yes

**Case control studies[2]**	**Confidence in exposure assessment**	**Confidence in cases but not controls acquiring outcome of interest**	**Proper selection of cases (those that were exposed and developed outcome of interest)**	**Proper selection of controls (those that were exposed and did not develop outcome of interest)**	**Matching of cases and controls according to prognostic variables or statistical adjustment for these variables**
Roberts et al. [15]	probably yes	probably yes	definitely yes	probably yes	definitely no

[2] CLARITY Group at McMaster University. Tool to Assess Risk of Bias in Case Control Studies [Available from: https://www.evidencepartners.com/wp-content/uploads/2021/03/Tool-to-Assess-Risk-of-Bias-in-Case-Control-Studies-DistillerSR.pdf.

Cross sectional studies	Exposed and non-exposed groups from the same population	Confidence in exposure assessment	Confidence that outcome of interest was not present at start of study	Matching of exposed and unexposed for all variables associated with outcome of interest or adjustment of prognostic variables by statistical analysis	Confidence in assessment of presence/absence of prognostic factors	Confidence in outcome assessment	Similar co-interventions between groups
Iuliano et al. [45]	probably yes	probably yes	definitely yes	definitely no	definitely no	definitely yes	probably no
Shi et al. [46]	definitely yes	definitely yes	probably yes	definitely no	definitely yes	definitely yes	probably yes
Somekh et al. [17]	probably yes	definitely yes	probably no	definitely no	probably no	definitely yes	probably yes

Supplementary table 3: Risk of bias assessment - Child/Adolescent risk of severe disease when infected with a VOC

**Cohort studies**[1]	**Exposed and non-exposed cohorts from the same population**	**Confidence in exposure assessment**	**Confidence that outcome of interest was not present at start of study**	**Matching of exposed and unexposed for all variables associated with outcome of interest or adjustment of prognostic variables by statistical analysis**	**Confidence in assessment of presence/absence of prognostic factors**	**Confidence in outcome assessment**	**Adequate follow-up of cohorts**	**Similar co-interventions between groups**
Butt et al. [31]	probably yes	definitely yes	probably yes	probably yes	probably yes	definitely yes	definitely no	probably yes

**Cross sectional studies**	**Exposed and non-exposed groups from the same population**	**Confidence in exposure assessment**	**Confidence that outcome of interest was not present at start of study**	**Matching of exposed and unexposed for all variables associated with outcome of interest or adjustment of prognostic variables by statistical analysis**	**Confidence in assessment of presence/absence of prognostic factors**	**Confidence in outcome assessment**	**Similar co-interventions between groups**
Shi et al. [46]	definitely yes	definitely yes	probably yes	definitely no	definitely yes	definitely yes	probably yes

[1] CLARITY Group at McMaster University. Tool to Assess Risk of Bias in Cohort Studies [Available from: https://www.evidencepartners.com/wp-content/uploads/2021/03/Tool-to-Assess-Risk-of-Bias-in-Cohort-Studies-DistillerSR.pdf.

Supplementary table 4: Risk of bias assessment - Child/Adolescent risk of becoming infected with a VOC

Cohort studies[1]	Exposed and non-exposed cohorts from the same population	Confidence in exposure assessment	Confidence that outcome of interest was not present at start of study	Matching of exposed and unexposed for all variables associated with outcome of interest or adjustment of prognostic variables by statistical analysis	Confidence in assessment of presence/absence of prognostic factors	Confidence in outcome assessment	Adequate follow-up of cohorts	Similar co-interventions between groups
Nakel et al. [19]	probably yes	definitely yes	probably yes	definitely no	definitely no	probably no	definitely no	probably yes
Somekh et al. [33]	probably yes	definitely yes	probably yes	probably no	probably no	definitely yes	probably yes	probably no
Abu Raddad et al. [57]	definitely yes	definitely yes	probably no	definitely no	probably no	probably yes	definitely yes	probably no
Loenenbach et al. [55]	probably yes	probably yes	probably no	definitely no	definitely no	definitely yes	probably no	probably no
Lorthe et al. [56]	definitely yes	definitely yes	probably yes	definitely no	probably yes	probably yes	definitely yes	definitely yes
Clifford et al. [58]	probably yes	probably yes	definitely yes	definitely no	definitely no	definitely yes	probably no	probably yes
Dougherty et al. [51]	probably yes	definitely yes	definitely yes	probably no	probably yes	definitely yes	probably yes	probably no
Singanayagam et al. [52]	probably yes	probably yes	probably yes	probably no	probably no	definitely yes	probably yes	probably no
Schenk et al. [53]	probably yes	probably yes	probably no	probably no	probably no	probably yes	probably no	probably yes
Lorthe et al. [59]	probably yes	definitely yes	definitely no	definitely no	definitely no	definitely yes	probably no	definitely no
Waltenburg et al. [18]	probably yes	probably yes	probably yes	probably no	probably no	probably yes	definitely no	probably yes
Ng et al. [50]	probably yes	probably yes	probably yes	probably no	definitely no	probably yes	probably yes	probably yes

[1] CLARITY Group at McMaster University. Tool to Assess Risk of Bias in Cohort Studies [Available from: https://www.evidencepartners.com/wp-content/uploads/2021/03/Tool-to-Assess-Risk-of-Bias-in-Cohort-Studies-DistillerSR.pdf.

Case control studies[2]	Confidence in exposure assessment	Confidence in cases but not controls acquiring outcome of interest	Proper selection of cases (those that were exposed and developed outcome of interest)	Proper selection of controls (those that were exposed and did not develop outcome of interest)	Matching of cases and controls according to prognostic variables or statistical adjustment for these variables
Roberts et al. [15]	probably yes	probably yes	definitely yes	probably yes	definitely no

Cross sectional studies	Exposed and non-exposed groups from the same population	Confidence in exposure assessment	Confidence that outcome of interest was not present at start of study	Matching of exposed and unexposed for all variables associated with outcome of interest or adjustment of prognostic variables by statistical analysis	Confidence in assessment of presence/absence of prognostic factors	Confidence in outcome assessment	Similar co-interventions between groups
Somekh et al. [17]	probably yes	definitely yes	probably no	definitely no	probably no	definitely yes	probably yes
Neuberger et al. [54]	probably yes	probably yes	probably yes	probably no	probably no	probably yes	probably yes

[2] CLARITY Group at McMaster University. Tool to Assess Risk of Bias in Case Control Studies [Available from: https://www.evidencepartners.com/wp-content/uploads/2021/03/Tool-to-Assess-Risk-of-Bias-in-Case-Control-Studies-DistillerSR.pdf.

Supplementary table 5: Risk of bias assessment - Child/adolescent risk of transmission when infected with a VOC

Cohort studies[1]	Exposed and non-exposed cohorts from the same population	Confidence in exposure assessment	Confidence that outcome of interest was not present at start of study	Matching of exposed and unexposed for all variables associated with outcome of interest or adjustment of prognostic variables by statistical analysis	Confidence in assessment of presence/absence of prognostic factors	Confidence in outcome assessment	Adequate follow-up of cohorts	Similar co-interventions between groups
Somekh et al. [33]	probably yes	definitely yes	probably yes	probably no	probably no	definitely yes	probably yes	probably no
Buchan et al. [63]	definitely yes	probably yes	probably no	definitely no	definitely no	probably yes	probably no	probably no
Lindstrom et al. [66]	definitely yes	definitely yes	definitely no	probably no	definitely no	probably yes	probably no	probably yes
Loenenbach et al. [55]	probably yes	probably yes	probably no	definitely no	definitely no	definitely yes	probably no	probably no
Lorthe et al. [56]	definitely yes	definitely yes	probably yes	definitely no	probably yes	probably yes	definitely yes	definitely yes
Julin et al. [67]	probably yes	definitely yes	definitely yes	probably no	definitely no	definitely yes	definitely yes	probably yes
Lyngse et al. [60]	definitely yes	definitely yes	definitely yes	probably yes	probably yes	probably yes	probably yes	probably no
Chudasama et al. [61]	probably yes	probably no	probably no	definitely no	definitely no	probably yes	probably no	definitely no
Lorthe et al. [59]	probably yes	definitely yes	definitely no	definitely no	definitely no	definitely yes	probably no	definitely no
Trobajo-Sanmartin et al. [65|	probably yes	definitely yes	probably yes	probably yes	probably yes	definitely yes	probably no	probably yes
Waltenburg et al. [18]	probably yes	probably yes	probably yes	probably no	probably no	probably yes	definitely no	probably yes
Ng et al. [50]	probably yes	probably yes	probably yes	probably no	definitely no	probably yes	probably yes	probably yes

[1] CLARITY Group at McMaster University. Tool to Assess Risk of Bias in Cohort Studies [Available from: https://www.evidencepartners.com/wp-content/uploads/2021/03/Tool-to-Assess-Risk-of-Bias-in-Cohort-Studies-DistillerSR.pdf.

Case control studies[2]	Confidence in exposure assessment	Confidence in cases but not controls acquiring outcome of interest	Proper selection of cases (those that were exposed and developed outcome of interest)	Proper selection of controls (those that were exposed and did not develop outcome of interest)	Matching of cases and controls according to prognostic variables or statistical adjustment for these variables
Loss et al. [62]	probably yes	definitely yes	definitely yes	definitely yes	probably no
Allen et al. [64]	definitely yes	definitely yes	definitely yes	definitely yes	probably yes

Modelling studies[3]	Is the population relevant?	Are any critical interven-tions missing?	Are any relevant out-comes missing?	Is the context (settings and circum-stances) applicable?	Is external validation of the model sufficient to make its results credible for your decision?	Is internal verification of the model sufficient to make its results credible for your decision?	Does the model have sufficient face validity to make its results credible for your decision?	Is the design of the model adequate for your decision problem?	Are the data used in populating the model suitable for your decision problem?	Were the analyses performed using the model adequate to inform your decision problem?	Was there an adequate assessment of the effects of uncertainty?	Was the reporting of the model adequate to inform your decision problem?	Was the interpre-tation of results fair and balanced?	Were there any potential conflicts of interest?	If there were potential conflicts of interest, were steps taken to address these?
Ratman et al. [68]	definitely yes	probably yes	probably no	probably yes	definitely no	definitely no	probably no	probably yes	probably yes	probably yes	definitely no	probably yes	probably yes	definitely no	probably no

[2] CLARITY Group at McMaster University. Tool to Assess Risk of Bias in Case Control Studies [Available from: https://www.evidencepartners.com/wp-content/uploads/2021/03/Tool-to-Assess-Risk-of-Bias-in-Case-Control-Studies-DistillerSR.pdf.
[3] Jaime Caro J, Eddy DM, Kan H, Kaltz C, Patel B, Eldessouki R, et al. Questionnaire to assess relevance and credibility of modeling studies for informing health care decision making: an ISPOR-AMCP-NPC Good Practice Task Force report. Value Health. 2014;17(2):174-82. doi: 10.1016/j.jval.2014.01.003.

Supplementary table 6: Risk of bias assessment - Risk of long-term effects when infected with a VOC

Cohort studies[7]	Exposed and non-exposed cohorts from the same population	Confidence in exposure assessment	Confidence that outcome of interest was not present at start of study	Matching of exposed and unexposed for all variables associated with outcome of interest or adjustment of prognostic variables by statistical analysis	Confidence in assessment of presence/absence of prognostic factors	Confidence in outcome assessment	Adequate follow-up of cohorts	Similar co-interventions between groups
Molteni et al. [38]	definitely yes	probably yes	probably yes	probably yes	probably no	probably yes	probably yes	probably no
Kildegaard et al. [27]	probably yes	probably yes	probably yes	definitely no	probably no	definitely yes	definitely yes	probably yes

[7]CLARITY Group at McMaster University. Tool to Assess Risk of Bias in Cohort Studies [Available from: https://www.evidencepartners.com/wp-content/uploads/2021/03/Tool-to-Assess-Risk-of-Bias-in-Cohort-Studies-DistillerSR.pdf.
