# Supplementary material for: SARS-CoV-2 variants of concern in children and adolescents with COVID-19: a systematic review
Source: BMJ Open. 2023 Oct 9;13(10):e072280. doi: 10.1136/bmjopen-2023-072280 (PMC10565293; doi:10.1136/bmjopen-2023-072280)
Supplement: Supplementary data [file bmjopen-2023-072280supp008.pdf]

Supplementary table 7: Review question 1 - Child/adolescent symptoms/severity of disease when infected with the alpha variant (n=9)

Author	Study design	Country	Study setting	Number of study participants <20 yo[1]	Number of adult study participants	Age groups	Mean/ Median age	VOC[2]	Symptoms/Severity	Comments
Roberts et al. [15]	case-control study	United Kingdom	schools kindergartens crèches	overall: 83<br><br>infected: 21	34	≤4yo, adults	n/r	Alpha	**Symptoms**<br>24% fever, 19% cough (52% asymptomatic)	**sequencing:** 1 case was sequenced, rest was assumed based on date of sampling and national prevalence
Ulyte et al. [16]	cohort study	Switzerland	schools kindergartens crèches	overall: 2 487<br><br>newly infected: 182	n/a	7-17 yo	median: 12 yo	Alpha	**Symptoms**<br>17% fatigue (30/182), 17% sore throat (30/182), 15% headache (27/182), 14% fever (26/182)	**sequencing:** n/r
Somekh et al. [17]	cross-sectional study	Israel	households	alpha infection: 31<br><br>non-VOC infection: 20	n/a	11-17 yo	n/r	Alpha	**Symptoms (alpha vs. non-VOC)**<br>sensory impairment: 6.5% (2/31) vs. 40% (8/20)<br>(RR[3]: 0.16; 95%CI[4]: 0.04–0.6)	**sequencing:** n/r

[1] yo: years old
[2] VOC: Variants Of Concern
[3] RR: Risk Ratio
[4] 95%CI: 95% Confidence Interval

Wiedenmann M, et al. BMJ Open 2023; 13:e072280. doi: 10.1136/bmjopen-2023-072280

Waltenburg et al. [18]	cohort study	USA[5]	households	n/r	n/r	<5 yo, 5-11 yo, 12-17 yo	median: 12 yo (IQR[6] 7-15)	Alpha	**Symptoms (alpha vs. non-VOC)**<br>constitutional (chills, fever, myalgia): OR[7] 5.5 (95%CI: 1.55-19.20)<br>lower respiratory (e.g. cough): OR 3.52 (95%CI: 1.26-9.86)<br>gastrointestinal (e.g. diarrhoea, pain): OR 3.55 (95%CI: 1.16-10.89)<br>**Severity (alpha vs. non-VOC):**<br>Symptom duration (median, days): 3 vs. 2, p[8] = 0.64	**vaccination status:** 97% unvaccinated (147/151), 3% partially vaccinated (4/151)<br>**sequencing:** majority of primary cases sequenced
Nakel et al. [19]	cohort study	Germany	schools kindergartens crèches	infected: 40<br>alpha infection: 18<br>non-VOC infection: 22	n/a	≤6 yo	n/r	Alpha	**Symptoms (alpha vs. non-VOC)**<br>fever: 38.9% (7/18) vs. 50% (11/22)<br>cough: 44.4% (8/18) vs. 36.4% (8/22)<br>shortness of breath: 11.1% (2/18) vs. 0% (0/22)<br>**Severity (alpha vs. non-VOC)**<br>ICU[9] admission: 0 vs. 0	**sequencing:** partially
Somekh et al. [33]	cohort study	Israel	hospital inpatients hospital outpatients	alpha infection: 72 796<br>non-VOC infection: 26 689	n/a	≤9 yo	n/r	Alpha	**Severity (alpha vs. non-VOC)**<br>Hospitalisation: 0.52% (379/72796) vs. 0.98% (261/26689)<br>RR 0.53 (95% CI: 0.46-0.63), p < 0.001<br>Severe disease/death: 6.9% (26/379) vs. 6.5% (17/261)<br>RR 0.99 (95% CI: 0.96-1.04)	**sequencing:** n/r
Swann et al. [20]	cohort study	United Kingdom	hospital inpatients	infected: 1 617<br>alpha infection: 952<br>non-VOC infection: 665	n/a	≤19 yo	median (alpha): 6.5 yo (IQR 0.3-14.9)<br>median (non-VOC): 4.0 yo (IQR 0.4-13.6)	Alpha	**Symptoms (alpha vs. non-VOC)**<br>fever: 57.1% (544/952) vs. 73.8% (491/665), p<0.001<br>**Severity (alpha vs. non-VOC)**<br>Length of hospitalisation: 2 days (IQR 1-4) vs. 2 days (IQR 1-4), p=0.079<br>critical care: 12.0% (109/910) vs. 12.7 (78/616), p=0.989	**sequencing:** n/r

[5] USA: United States of America
[6] IQR: Interquartile Range
[7] OR: Odd Ratio
[8] p: p-value
[9] ICU: Intensive Care Unit

Wiedenmann M, *et al. BMJ Open* 2023; 13:e072280. doi: 10.1136/bmjopen-2023-072280

Brookman et al. [34]	cohort study	United Kingdom	hospital outpatients	infected: 80<br><br>alpha infection: 60<br><br>non-VOC infection: 20	n/a	≤18 yo	n/r	Alpha	**Severity (alpha vs. non-VOC)**<br>Critical disease: 3% (2/60) vs. 20% (4/20)<br>Oxygen: 8% (5/60) vs. 35% (7/20)<br>non-invasive ventilation: 3% (2/60) vs. 15% (3/20)<br>invasive ventilation: 2% (1/60) vs. 20% (4/20)	**sequencing:** n/r
Stewart et al. [21]	cohort study	United Kingdom	hospital inpatients	infected: 110<br><br>alpha infection: 67<br>non-VOC infection: 43	n/a	<18 yo	median overall: 10.2 yo (IQR 7.6-12.6)	Alpha	**Symptoms (alpha vs. non-VOC)**<br>PIMS-TS[10]: 67 vs. 43 cases<br>acute kidney injury in PIMS-TS:<br>28.4% (19/67) vs. 33% (14/43), p=0.85	**sequencing:** n/r

[10] [6]PIMS-TS/MIS-C: Paediatric Inflammatory Multisystem Syndrome/Multisystem Inflammatory Syndrome in Children

Wiedenmann M, *et al. BMJ Open* 2023; 13:e072280. doi: 10.1136/bmjopen-2023-072280

Supplementary table 8: Review question 1 - Child/adolescent symptoms/severity of disease when infected with the delta variant (n=13)

Author	Study design	Country	Study setting	Number of study participants <20 yo[1]	Number of adult study partici-pants	Age groups	Mean/ Median age	VOC[2]	Symptoms/Severity	Comments
Li et al. [22]	cohort study	China	hospital inpatients	infected: 77	n/a	<12 yo	median: 9 yo	Delta	**Symptoms**<br>75.3% fever (58/77), 18.2% cough (14/77), 7.8% expectoration (6/77)	**sequencing:** all<br>everyone was hospitalised for isolation
Hao et al. [23]	cohort study	China	hospital inpatients	infected: 66	n/a	≤18 yo	mean (±SD[3]): 10.35±5.86 yo	Delta	**Symptoms**<br>47.0% fever (31/66), 47.0% cough (31/66), 34.8% nasal congestion (23/66), 25.8% sore throat (17/66), 10.6% fatigue (7/66), 4.5% decreased sense of smell (3/66)	**Sequencing:** n/r
Nygaard et al. [48]	cohort study	Denmark	hospital inpatients	infected: 74<br><br>delta infection : 51<br><br>non-VOC infection: 23	n/a	≤17 yo	n/r	Delta	**MIS-C[4] symptoms (delta vs. non-VOC)**<br>hypotension/shock: 51% (26/51) vs. 57% (13/23)<br>cardiac involvement: 90% (46/51) vs. 100% (23/23)<br>gastrointestinal involvement: 98% (50/51) vs. 100% (23/23)<br>respiratory involvement: 12% (6/51) vs. 26% (6/23)<br><br>**MIS-C severity (delta vs. non-VOC)**<br>ICU[5] admission: 55% (28/51) vs. 52% (12/23)<br>vasoactive support: 16% (8/51) vs. 22% (5/23)<br>no need for mechanical ventilation or ECMO[6], no deaths for both delta and non-VOC infection<br><br>(risk of MIS-C comparable with delta and non-VOC infection)	**vaccination status:** none vaccinated<br><br>**sequencing:** majority sequenced

[1] yo: years old
[2] VOC: Variants Of Concern
[3] SD: Standrad Deviation
[4] MIS-C: Multisystem Inflammatory Syndrome in Children
[5] ICU: Intensive Care Unit
[6] ECMO: Extracorporeal Membrane Oxygenation

Murillo-Zamora et al. [37]	cohort study	Mexico	hospital inpatients	infected: 26 961<br><br>person-days: 297 099	n/r	≤19 yo	mean (±SD): 14.4±5.8 yo	Delta	**Severity (delta vs. pre-delta)**<br>pneumonia: incidence rate 30.3/10 000 person-days vs. 9.4/10 000 person-days<br>RR[7] 0.98 (95%CI: 0.97-0.99), p[8]<0.001	**sequencing:** n/r<br>no information on pre-delta strains
Ryu et al. [24]	cohort study	South Korea	population	infected: 235<br><br>delta infection: 92<br><br>pre-delta infection: 143	n/r	<18 yo	mean (delta): 10.2 yo<br><br>mean (pre-delta): 13.8 yo<br>p<0.001	Delta	**Symptoms (delta vs. pre-delta)**<br>rhinorrhoea: 10.5% (15/143) vs. 25% (23/92), p=0.003<br>nasal stuffiness: 15.4% (22/143) vs. 34.8% (32/92), p=0.001<br>sore throat: 12.6% (18/143) vs. 23.9% (22/143), p=0.02<br><br>no difference in prevalence of other COVID symptoms (e.g. fever, cough, pneumonia)	**sequencing:** n/r<br>no information on pre-delta strains
Fisman et al. [38]	cohort study	Canada	population	infected: 20 434<br><br>delta infection: 12 387<br><br>non-VOC infection: 8 047	n/a	≤19 yo	n/r	Delta	**Severity (delta vs. non-VOC)**<br>hospitalization (<10 yo): OR[9] 2.51 (95%CI: 1.23-5.11)<br>hospitalization (10-19 yo): OR 0.78 (95%CI: 0.38-1.91)<br><br>ICU admission (10-19 yo): OR 1.37 (95%CI: 0.11-16.46)	**sequencing:** all sequenced
Shoji et al. [39]	cohort study	Japan	hospital inpatients	infected: 1 299<br><br>delta infection: 349<br><br>pre-delta infection: 950	n/a	<18 yo	median (delta): 7.0 yo (IQR[10]: 2-13)<br><br>median (pre-delta): 10.0 yo (IQR: 4-15)<br>p<0.001	Delta	**Severity (delta vs. pre-delta)**<br>ICU admission: 1.4% (5/349) vs. 0.1% (1/950), p=0.006<br>length of hospitalisation (median, days): 7.0 (IQR 5.0-9.0) vs. 8.0 (IQR 5.0-9.0), p=0.031<br>mechanical ventilation: 0% vs. 0%	**sequencing:** n/r<br>no information on pre-delta strains

[7] RR: Risk Ratio
[8] p: p-value
[9] OR: Odd Ratio
[10] IQR: Interquartile Range

Martin et al. [40]	cohort study	USA[11]	hospital inpatients hospital outpatients	infected: 167 262<br><br>delta infection: 29 191<br><br>pre-delta infection: 138 071	n/a	<19 yo	median (delta): 10.3 yo (IQR 5.3-14.9)<br><br>median (pre-delta): 12.3 yo (IQR 6.2-16.4)	Delta	**Severity (delta vs. pre-delta)**<br>hospitalisation: 6.0% (1738/29191) vs. 6.2% (8507/138071), p=0.18<br>severe disease: 10.3% (179/1738) vs. 14.6% (1242/8507), (OR 0.67, 95%CI: 0.57-0.79), p<0.001<br>severe disease (children classified as non-black, non-white): OR 1.69 (95%CI: 1.02-2.82, p=0.04	**sequencing:** n/r<br>no information on pre-delta strains
Butt et al. [25]	cohort study	Qatar	population	infected: 246<br><br>delta infection: 125<br><br>beta infection: 121	n/a	≤19 yo	n/a	Beta, Delta	**Symptoms (delta vs. beta)**<br>moderate disease: aOR[12] 0.17 (0.06-0.48) vs. 0.35 (0.09-1.39), p=0.54<br>severe disease: aOR 0.08 (0.01-0.48) vs. NA, p>0.99	**sequencing:** all sequenced
Molteni et al. [26]	cohort study	United Kingdom	population	infected: 1 400<br><br>delta infection: 706<br>5-11 yo: 227<br>12-17 yo: 479<br><br>alpha infection: 694<br>5-11 yo: 276<br>12-17 yo: 418	n/r	5-17 yo	median (alpha): 12 yo (IQR 9-15)<br><br>median (delta): 14 yo (IQR 13-16)	Alpha, Delta	**Symptoms (delta vs. alpha)**<br>5-11yo<br>headache: 60.8% (138/227) vs. 39.9% (110/276), OR 2.31 (95%CI: 1.6-3.33), p<0.001<br>fever: 46.3% (105/227) vs. 31.9% (88/276), OR 1.89 (95%CI: 1.31-2.73) (p=0.001)<br>12-17yo<br>headache: 73.7% (353/479) vs. 61.5% (257/418), OR 1.81 (95%CI: 1.36-2.42), p<0.001<br>fever: 43.2% (207/479) vs. 27.0% (113/418), OR 2.05 (95%CI: 1.55-2.72), p<0.001<br>rhinorrhoea: 57.6% (276/479) vs. 47.4% (198/418), OR 1.52 (95%CI: 1.17-1.98), p=0.002<br>sore throat: 60.5% (290/479) vs. 45.7% (191/418), OR 1.87 (95%CI: 1.43-2.45), p<0.001<br><br>**Severity (delta vs. alpha)**<br>symptom burden (5-11 yo, median/days): 4 (IQR 2-7) vs 3 (IQR 2-5)<br>symptom burden (12-17 yo, median/days): 6 (IQR 3-9) vs 5 (IQR 3-8)	**sequencing:** n/r

[11] USA: United States of America
[12] aOR: Adjusted Odd Ratio

Kildegaard et al. [27]	cohort study	Denmark	Hospital inpatients and outpatients	infected: 74 611<br><br>alpha infection: n/r<br><br>delta infection: n/r	n/a	<18 yo	Median (infected): 8 yo (IQR 1-14)	Alpha, Delta	**Severity (alpha vs. delta)**<br>hospitalization: RR[13] 0.49% (98/20102) (95%CI[14]: 0.4-0.59) vs. 0.48% (18/16694) (95%CI: 0.38-0.6)<br>MIS-C: RR 0.04% (9/20164) (95%CI: 0.02-0.08) vs. 0.04% (5/12778) (95%CI: 0.01-0.09)	**vaccination status:** partially vaccinated<br><br>**sequencing:** n/r
Çelebi et al. [41]	cohort study	Turkey	hospital inpatients hospital outpatients	infected: 680<br><br>alpha infection: 329<br><br>beta/gamma infection: 17<br><br>delta infection: 165<br><br>non-VOC infection: 169	n/a	≤17 yo	median: 9.25 yo	Alpha, Beta, Delta	**Severity (alpha vs. delta vs. beta/gamma vs. non-VOC)**<br>hospitalization: 9.4% (31/329) vs. 19.4% (32/165) vs. 18% (3/17) vs. 10.1% (17/169)<br><br>**Severity (alpha vs. delta)**<br>hospitalization: 9.4% (31/329) vs. 19.4% (32/165), p=0.016<br><br>**Severity (delta vs. non-VOC)**<br>hospitalization: 19.4% (32/165) vs. 10.1% (17/169), p=0.002	**sequencing:** all sequenced, but no differentiation between gamma and beta

[13] RR: Risk Ratio
[14] 95%CI: 95% Confidence Interval

Edward et al. [28]	cohort study	USA	hospital outpatients	infected: 499<br><br>alpha infection: 96<br><br>gamma infection: 38<br><br>delta infection: 119<br><br>non-VOC infection: 243	n/a	≤18 yo	median (alpha): 9 yo (IQR 2-14)<br><br>median (gamma): 7 yo (IQR 1-14)<br><br>median (delta): 4 yo (IQR 1-11)<br><br>median (non-VOC): 7 yo (IQR 1-12)	Alpha, Delta, Gamma	**Symptoms (alpha vs. gamma vs. delta vs. non-VOC)**<br>symptomatic: 91.6% (88/96) vs. 89.4% (34/38) vs. 89.9% (107/119) vs. 91.7% (223/243), p=0.92<br><br>**Severity - Hospitalization**<br>non-VOC: 3.2% (8/243) reference<br>alpha: 7.2% (7/96): OR 1.9 (95%CI: 0.59-6.3)<br>gamma: 15.7% (6/38): OR 5.9 (95%: 1.6-21.5), p=0.007<br>delta: 6.7% (8/119): OR 2.2 (95%CI: 0.69-7.7)<br><br>**ICU admission**<br>non-VOC: 1.2% (3/243): reference<br>alpha: 1.0% (1/96) :OR 0.47 (95%CI: 0.02-4.0)<br>gamma: 5.2% (2/38): OR 2.6 (95%CI: 0.31-17.5)<br>delta: 2.5% (3/119): OR 1.4 (95%CI: 0.24-9.0)<br><br>**Respiratory support**<br>non-VOC: 1.2% (3/243): reference<br>alpha: 0% : OR 0<br>gamma: 10.5% (4/38) : OR 8.3 (95%CI: 1.5-56.3), p=0.02<br>delta: 2.5% (3/119) : OR 2.2 (95%CI: 0.34-16.9)	**sequencing:** all sequenced

Supplementary table 9: Review question 1 - Child/adolescent symptoms/severity of disease when infected with the omicron variant (n=11)

Author	Study design	Country	Study setting	Number of study participants <20 yo[1]	Number of adult study partici-pants	Age groups	Mean/ Median age	VOC[2]	Symptoms/severity	Comments
Cloete et al. [29]	cohort study	South Africa	hospital inpatients	infected: 139	n/a	≤13 yo	n/r	Omicron	**Symptoms**<br>47% fever, 40% cough, 24% vomiting, 23% difficulty breathing, 20% convulsions<br><br>**Severity**<br>hospitalization: 92% standard ward care, 25% oxygen therapy, 2% high flow oxygen, 6% ventilation	**sequencing:** partially sequenced
Brewster et al. [14]	cohort study	USA[3]	hospital inpatients, hospital outpatients	infected: 75<br><br>omicron: 61<br><br>pre-omicron: 14	n/a	n/r	median (omicron): ED[4]: 2.4 yo (0.8–2.6) hospitalized: 1.6 yo (0.7–1.1)<br><br>median (pre-omicron): ED: 2.6 yo (1.6–3.4) hospitalized: 1.3 yo (1.1–1.5)	Omicron	**Symptoms (omicron vs. pre-omicron):**<br>Croup: 81% (61/75) vs. 19% (14/75)<br><br>Median weekly croup cases (omicron vs. pre-omicron): 11 [IQR[5] 2–17] vs. 0 (IQR 0-0), p[6] < 0.001<br><br>none required invasive ventilation or died (irrespective of variant)	**sequencing:** n/r
Ludvigsson et al. [30]	case series (>1 case)	Sweden	hospital inpatients	infected: 3	n/r	<15 yo	n/r	Omicron	**Symptoms**<br>**Case 1:** repeated convulsions over several hours, hospitalisation: 4 days<br>**Case 2:** status epilepticus, hospitalisation: 2 days<br>**Case 3:** one convulsion (30-60sec), short behavioural change, hospitalisation: 2 days	**sequencing:** n/r<br>14yo: clinical diagnosis

[1] yo: years old
[2] VOC: Variants Of Concern
[3] USA: United States of America
[4] ED: Emergency Department
[5] IQR: Interquartile Range
[6] p: p-value

Butt et al. [31]	cohort study	Qatar	population	Infected: 985<br><br>(both the omicron and delta cohort)	n/r	<18 yo	median (delta): 7 yo (IQR 3-9)<br><br>median (omicron): 6 yo (IQR 3-10)	Delta, Omicron	**Severity (omicron vs. delta)**<br>mild/not hospitalised: 97.8% (963/985) vs. 84.2% (829/985), p<0.001<br>moderate disease: 2.2% (22/985) vs. 15.7% (155/985)<br>critical disease: 0% (0/985) vs. 0.1% (1/985)<br><br>moderate+critical disease: 2.2% (22.985) vs. 15.8% (156/985), p<0.001, aOR[7] 0.12 (95%CI: 0.07-0.18)	**vaccination status:** none vaccinated<br><br>**sequencing:** n/r
Fowlkes et al. [32]	cohort study	USA	population	infected: 252<br><br>omicron infection: 150<br><br>delta infection: 102	n/r	5-11 yo, 12-15 yo	n/r	Delta, Omicron	**Symptoms (omicron vs. delta)**<br>COVID[8]- symptoms: 48.7% (73/150) vs. 65.7% (67/102); OR[9] 2.0 (95%CI[10]: 1.2-3.45), p=0.008'<br><br>**Severity (omicron vs. delta)**<br>total days of symptoms: 5.4% vs. 8.0%; OR -3.4 (95%CI: (-)5.7 - (-)1.0), p=0.006<br>hours of missed school: 21.8% vs. 24.1%**;** OR -10.6 (95%CI: (-)18.6 - (-)2.7), p=0.01<br><br>(no differences in febrile symptoms, received medical care, days spent sick in bed)	**vaccination status:**<br>5-11 yo: 65% (682/1052) double vaccinated, 7% (69/1052) 1 dose, and 29% (301/1052) unvaccinated.<br>12–15 yo: 68% (212/312) double vaccinated, 5% (15/312) 1 dose, 27% (85/312) unvaccinated<br><br>**sequencing:** n/r
Wang et al. [42]	cohort study	USA	hospital inpatients hospital outpatients	infected: 7 198<br><br>(both the omicron and the delta cohort)	n/r	<5 yo	mean (omicron): 1.49±1.42<br><br>mean (delta): 1.48±1.42	Delta, Omicron	**Severity (omicron vs. delta)**<br>ED visits: 18.83 %(1355/7198) vs. 26.67 (1920/7198); RR[11] 0.71 (95% CI: 0.66-0.75)<br>hospitalisation: 1.04% (75/7198) vs. 3.14 (226/7198); RR 0.33 (95%CI: 0.26-0.43)<br>ICU[12] admission: 0.14% (10/7198) vs. 0.43% (31/7198); RR 0.32 (95%CI: 0.16-0.66)<br>mech. ventilation: 0.33% (24/7198) vs. 1.15% (83/7198); RR 0.29 (95%CI: 0.18-0.46)	**sequencing:** n/r

[7] aOR: Adjusted Odd Ratio
[8] COVID: Coronavirus Disease
[9] OR: Odd Ratio
[10] 95%CI: 95% Confidence Interval
[11] RR: Risk Ratios
[12] ICU: Intensive Care Units

Wiedenmann M, *et al. BMJ Open* 2023; 13:e072280. doi: 10.1136/bmjopen-2023-072280

Wang et al. [43]	cohort study	USA	hospital inpatients hospital outpatients	infected ≤4 yo: 11 556 (both the omicron and the delta cohort)<br><br>infected 5-17 yo: 18 715 (both the omicron and the delta cohort)	n/a	≤4 yo, 5-17yo	n/a	Delta, Omicron	**Severity (omicron vs. delta)**<br>≤ 4yo<br>ED visits: 20.41% (2359/11556) vs. 23.96% (2796/1156); RR 0.85 (95%CI: 0.81-0.89)<br>hospitalization: 1.55% (179/11556) vs. 3.0% (347/11556); RR 0.52 (95%CI: 0.43-0.62)<br>ICU admissions: 0.1% (12/11556) vs. 0.42% (49/11556); RR 0.25 (95%CI: 0.13-0.46)<br>mech. ventilation: 0.09% (10/11556) vs. 0.2% (23/11556); RR 0.44 (95%CI: 0.21-0.91)<br><br>5-17yo<br>ED visits: 10.09% (1889/18715) vs. 13.07% (2446/18715); RR 0.77 (95%CI: 0.73-0.82)<br>hospitalization: 0.77% (144/18715) vs. 1.37% (144/18715);<br>RR 0.56 (95%CI: 0.46-0.69)<br>ICU admissions: 0.23% (43/18715) vs. 0.38% (71/18715); RR 0.61 (95%CI: 0.42-0.88)<br>mech. ventilation: 0.05% (10/18715) vs. 0.06 (11/18715); RR 0.91 (95%CI: 0.39-2.14)	**sequencing:** n/r<br>**vaccination status:** n/r
Marks et al. [44]	cohort study	USA	hospital inpatients	infected: 2 100<br><br>omicron infection: 266<br><br>delta infection: 1 834	n/r	≤4 yo, 5-11 yo, 12-17yo	median: 7 yo (IQR 1-14)	Delta, Omicron	**Severity (omicron vs. delta)**<br>ICU admission (≤17 yo): 20.2% (52/266) vs. 27.8% (510/1834)<br>mech. ventilation (≤17 yo): 2.3% (6/266) vs. 6.3% (112/1834)<br><br>hospitalization (≤4yo): 54.2% (142/266) vs. 42.5% (778/1834); RR 5.4 (95%CI: 4.0-7.2)<br>hospitalization (5-11 yo): 16.9% (43/266) vs. 22.5% (417/1834); RR 2.3 (95%CI: 1.5-3.6)<br>hospitalization (12–17 yo): 28.9% (81/266) vs. 34.9% (639/1834); RR 3.5 (95%CI: 2.5-5.0)	**sequencing:** n/r<br>**vaccination status:** n/r
Iuliano et al. [45]	cross-sectional study	USA	hospital inpatients	infected: 824<br><br>omicron infection: 405<br><br>delta infection: 272<br><br>non-VOC infection: 147	n/a	≤17 yo	n/r	Delta, Omicron	**Severity (omicron vs. delta)**<br>ICU admission: 10.4% (42/405) vs. 18.4% (50/272)<br>(ICU admission non-VOC: 17.0% (25/147))	**sequencing:** n/r<br>**vaccination status:** n/r

Shi et al. [46]	cross-sectional study	USA	hospital inpatients	infected: 1 475<br><br>omicron infection: 397<br><br>delta infection: 482<br><br>pre-Delta infection: 596	n/a	5-11 yo	median: 8 yo (IQR 6-10)	Delta, Omicron	**Severity (omicron vs. delta)**<br>ICU admission: 18.9% vs. 26.1%, p=0.05<br>mech. ventilation: 4.6% vs. 6.8%, p=0.28<br>death: 0% vs. 0%<br><br>**Severity (omicron vs. pre-delta)**<br>ICU admission pre-Delta: 18.9% vs. 32.6%, p<0.01<br>mech. ventilation: 4.6% vs. 6.7%, p=0.28<br>death: 0%. vs. 0.7%<br><br>**underlying diseases (omicron vs. delta):**<br>neurologic disorders: 33% vs. 21%, p<0.01<br>obesity: 33% vs. 21%, p = 0.01	**sequencing:** n/r<br>**vaccination status:** n/r
Marks et al. [47]	cohort study	USA	Hospital inpatients	infected: 2 562<br><br>omicron infection: 572<br>delta infection: 790<br>pre-delta infection: 1 200	n/a	≤4 yo	median: 0.6yo (IQR 0.1–1.0)	Delta, Omicron	**Severity (omicron vs. delta vs. pre-delta):**<br>length of hospitalisation (days, median, IQR): 1.5 (0.5-2.5) vs. 2 (1-3.5) vs. 1.5 (1-3.5), p (omicron vs. pre-delta)= 0.001, p (omicron vs. delta)=0.002<br>ICU admission: 21.0 % vs. 26.7% vs. 24%, p (omicron vs. pre-delta)= 0.19, p (omicron vs. delta)=0.02<br>BiPAP/CPAP[13]: 5.1% vs. 9.1% vs. 5.9%, p (omicron vs. pre-delta)= 0.53, p (omicron vs. delta)=0.008<br>high flow nasal canula: 13.4% vs. 20.4% vs. 8.3%, p (omicron vs. pre-delta)= 0.002, p (omicron vs. delta)=0.002<br><br>No difference in invasive mechanical ventilation and in hospital deaths	**vaccination status:** none vaccinated<br><br>**sequencing:** n/r

[13] BiPAP/CPAP: Bilevel Positive Airway Pressure/ Continuous Positive Airway Pressure

Wiedenmann M, *et al. BMJ Open* 2023; 13:e072280. doi: 10.1136/bmjopen-2023-072280

Supplementary table 10: Review question 1 - Child/adolescent symptoms/severity of disease when infected with the gamma variant (n=2)

Author	Study design	Country	Study setting	Number of study participants <20 yo[1]	Number of adult study participants	Age groups	Mean/Median age	VOC[2]	Symptoms/Severity	Comments
Oliveira et al. [35]	cohort study	Brazil	hospital inpatients	infected: 21 591<br><br>gamma infection: 10 017<br><br>non-VOC infection: 11 574	n/r	<20 yo	mean (gamma): 4.0 yo (IQR[3] 0.6 - 14.7)<br><br>mean (non-VOC): 5.2 yo (IQR 1.0 - 14.4)	Gamma	**Severity (gamma vs. non-VOC)**<br>hypoxemia: 52.5% vs. 41.1%, p[4]<0.0001<br>ICU[5] admissions: 28.3% (2498/8830) vs. 24.9% (2745/11037), p<0.0001<br>invasive vent. support: 10.1% (974/9659) vs. 10.8% (1156/10733) p<0.0001	**sequencing:** n/r

[1] yo: years old
[2] VOC: Variant Of Concern
[3] IQR: Interquartile Range
[4] p: p-value
[5] ICU: Intensive Care Unit

Edward et al. [36]	cohort study	USA[6]	hospital outpatients	infected: 499<br><br>alpha infection: 96<br><br>gamma infection: 38<br><br>delta infection: 119<br><br>non-VOC infection: 243	n/a	≤ 18 yo	median (alpha): 9 yo (IQR 2-14)<br><br>median (gamma): 7 yo (IQR 1-14)<br><br>median (delta): 4 yo (IQR 1-11)<br><br>median (non-VOC): 7 yo (IQR 1-12)	Alpha, Delta, Gamma	**Symptoms (alpha vs. gamma vs. delta vs. non-VOC)**<br>symptomatic: 91.6% (88/96) vs. 89.4% (34/38) vs. 89.9% (107/119) vs. 91.7% (223/243), p=0.92<br><br>**Severity - Hospitalization**<br>non-VOC: 3.2% (8/243) reference<br>alpha: 7.2% (7/96): OR[7] 1.9 (95%CI[8]: 0.59-6.3)<br>gamma: 15.7% (6/38):<br>OR 5.9 (95%: 1.6-21.5), p=0.007<br>delta: 6.7% (8/119):<br>OR 2.2 (95%CI: 0.69-7.7)<br><br>**ICU admission**<br>non-VOC: 1.2% (3/243): reference<br>alpha: 1.0% (1/96):<br>OR 0.47 (95%CI: 0.02-4.0)<br>gamma: 5.2% (2/38):<br>OR 2.6 (95%CI: 0.31-17.5)<br>delta: 2.5% (3/119):<br>OR 1.4 (95%CI: 0.24-9.0)<br><br>**Respiratory support**<br>non-VOC: 1.2% (3/243): reference<br>alpha: 0%: OR 0<br>gamma: 10.5% (4/38):<br>OR 8.3 (95%CI: 1.5-56.3), p=0.02<br>delta: 2.5% (3/119)<br>OR 2.2 (95%CI: 0.34-16.9)	**sequencing:** all sequenced

[6] USA: United States of America
[7] OR: Odds Ratio
[8] 95%CI: 95% Confidence Interval

Wiedenmann M, *et al. BMJ Open* 2023; 13:e072280. doi: 10.1136/bmjopen-2023-072280

Supplementary table 11: Review question 3 - Child/Adolescent risk of becoming infected with a SARS-CoV-2 variant of concern (n=15)

Author	Study design	Country	Study setting	Number of contacts <20 yo[1]	Number of adult contacts	Age groups	Mean/ Median age	VOC[2]	Risk of infection compared to non-VOCs/other VOCs	Risk of infection compared to adults	Comments
Schenk et al. [53]	cohort study	Germany	schools kindergartens crèches	non-VOC: 577<br><br>alpha: 756	non-VOC: 334<br>alpha: 226	≤7 yo, adults	median: 4yo	Alpha	**Risk of infection (alpha vs. non-VOC)**<br>0% (0/756) vs. 1.2% (7/577)	**Risk of infection (children vs. adults)**<br>0% (0/756) vs. 0% (0/226)	**vaccination status:** n/r<br>**sequencing:** n/r
Neuberger et al. [54]	cross-sectional study	Germany	schools kindergartens crèches	n/r<br>8500 early childhood education and care centres	n/r	≤2 yo, 4-6 yo, ≥7 yo, adults	n/r	Alpha	n/a	**Risk of infection (children vs. adults)**<br>higher absolute case number,<br>but overall rate of infection still higher in adults	**vaccination status:** n/r<br>**sequencing:** n/r
Nakel et al. [19]	cohort study	Germany	schools kindergartens crèches	113	31	≤6 yo	n/r	Alpha	**Risk of infection (alpha vs. non-VOC)**<br>15.9% (18/113) vs. 64.7% (22/34)	**Risk of infection (children vs. adults)**<br>15.9% (18/113) vs. 35.5% (11/31)	**vaccination status:** n/r<br>**Sequencing:** few cases sequenced, presence of variant assumed for the rest based on date of sampling and national prevalence
Roberts et al. [15]	case-control study	United Kingdom	schools kindergartens crèches	83	34	≤4 yo, adults	n/r	Alpha	n/a	**Risk of infection AR[3] (children vs. adults)**<br>25.3% (21/83) vs. 70.6% (24/34)<br><br>**AR (children):**<br>- 0-1yo: 44%<br>- 1-2 yo: 31%<br>- 2-3yo: 31%<br>- 3-4 yo: 4%	**vaccination status:** n/r<br>**sequencing:** 1 case sequenced, presence of variant assumed for the rest based on date of sampling and national prevalence

[1] yo: years old
[2] VOC: Variant Of Concern
[3] AR : Attack Rate

Wiedenmann M, *et al. BMJ Open* 2023; 13:e072280. doi: 10.1136/bmjopen-2023-072280

Loenenbach et al. [55]	cohort study	Germany	schools kindergartens crèches	133	60	≤6 yo, adults	n/r	Alpha	n/a	**Risk of infection SAR[4] overall (children vs. adults)**<br>21.1% (28/133) vs. 31.7% (19/60)<br><br>**SAR per daycare centre (children vs. adults)**<br>1. 31% (15/49) vs. 53% (10/19)<br>2. 27% (7/26) vs. 28% (5/18)<br>3. 17% (6/36) vs. 17% (4/23)	**vaccination status:** n/r<br>**sequencing:** n/r
Lorthe et al. [56]	cohort study	Switzerland	schools kindergartens crèches	70	9	4-6 yo, adults	n/r	Alpha	n/a	**Risk of infection AR (children vs. adults)**<br>27.4% (22/70) vs. 22.2% (2/9)<br><br>**AR (children by classes)**<br>1. 62.9% (13/21)<br>2. 10.5% (2/19)<br>3. 16.7% (3/18)<br>4. 13.3% (2/15)	**vaccination status:** n/r<br>**sequencing:** all cases
Abu-Raddad et al. [57]	cohort study	Qatar	population	55 638<br><br><10 yo: 28 791<br><br>10-19 yo: 26 847	30-39yo: 81 760	<10 yo, 10-19 yo, adults	n/r	Alpha	n/a	**Risk of infection (children <10 yo vs. adults)**<br>1.7% (502/28791) vs. 3.6% (2966/81760)<br>OR[5] 0.35 (95%CI[6]: 0.32–0.39)<br><br>**Risk of infection (adolescents 10-19 yo vs. adults)**<br>3.6% (965/26847) vs. 3.6% (2966/81760)	**vaccination status:** n/r<br>**sequencing:** 85% of all cases sequenced

[4] SAR: Secondary Attack Rate
[5] OR: Odd Ratio
[6] 95%CI: 95% Confidence Interval

Somekh et al. [33]	cohort study	Israel	hospital inpatients hospital outpatients	72 426<br><br>Alpha: 50 811<br><br>non-VOC: 21 615	n/r	<10 yo	n/r	Alpha	**Risk of infection (alpha vs. non-VOC)**<br>slope of weekly adjusted incidence: 84.4 (95%CI: 71.1- 97.7) vs 39.1 (95%CI:23.9-54.3)	n/a	**sequencing:** n/r
Somekh et al. [17]	cross-sectional study	Israel	households	Alpha: 77<br><br>non-VOC: 58	n/r	≤5 yo, 6-17 yo	n/r	Alpha	**Risk of infection (alpha vs. non-VOC)**<br>≤5 yo: 72% (18/25) vs. 11.1% (2/18) RR[7] 7.8 (95%CI: 2.1–29.0)<br><br>6-17 yo: 75% (39/52) vs. 32.5% (13/40) RR 2.5 (95%CI: 1.6–4.0)	n/a	**sequencing:** n/r
Waltenburg et al. [18]	cohort study	USA[8]	households	Alpha : 61<br><br>non-VOC : 39	n/r	≤11 yo, 12-17 yo	Median : 12 yo (IQR[9] 7-15)	Alpha	**Risk of infection (alpha vs. non-VOC)**<br>64% (39/61) (95%CI: 51-76) vs. 56% (22/39) (95%CI: 40-72); OR 1.08 (95% CI: 0.40- 2.98)<br><br>**(≤11 yo vs. 12-17yo)**<br>78% (21/27) (95%CI: 58-91) vs. 53% (18/34) (95%CI: 35-70); OR 1.37 (95%CI:0.61-3.04)	n/a	**vaccination status:** 97% unvaccinated (147/151), 3% partially vaccinated (4/151)<br>**sequencing:** majority of primary cases sequenced
Clifford et al. [58]	cohort study	United Kingdom	households	67	406	<18 yo, adults	n/r	Delta	n/a	**Risk of infection (children vs. adults)**<br>RR 0.84 (95%CI: 0.66-0.99)<br><br>(risk of being infected by non-elderly adults)	**vaccination status:** n/r<br>**sequencing:** 63.1% of cases sequenced, rest was assumed based on date of sampling and national prevalence

[7] RR: Risk Ratio
[8] USA: United States of America
[9] IQR: Interquartile Range

Ng et al. [50]	cohort study	Singapore	households	1 136	7223	≤11 yo, 12-17 yo, adults	median : 36 yo (IQR 26-51)	Delta	n/a	**Risk of infection SAR (≤11 yo vs. 18-29 yo)** 22.9% (173/757) vs. 12.0% (212/1765) ; aOR[10] 1.43 (95%CI: 1.07-1.93), p=0.017<br><br>**SAR (12-17 yo vs. 18-29 yo)** 12.9% (49/379) vs. 12.0% (212/1765) ; aOR 0.97 (95%CI: 0.66-1.42), p[11]=0.87	**vaccination status:** >12 yo partially vaccinated (results adjusted for vaccination status) **sequencing:** partially sequenced
Dougherty et al. [51]	cohort study	USA	camps, sport clubs	122	11	<12 yo, 12-19 yo, adults	median: 14 yo (IQR 5-58)	Delta	n/a	**Risk of infection AR (children vs. adults)** 18.9% (23/122) vs. 27.3% (3/11)	**vaccination status:** majority unvaccinated/partially vaccinated **sequencing:** 21 sequenced, rest was assumed based on date of sampling and national prevalence
Singana-yagam et al. [52]	cohort study	United Kingdom	community	30	198	5-18 yo, adults	median: 41 yo (IQR 28-49)	Delta	n/a	**Risk of infection SAR (children vs. adults)** 40% (12/30) vs. 20.1% (41/198)	**vaccination status:** 70% were fully vaccinated, 23.7% were partially vaccinated, 23% were not vaccinated **sequencing:** partially sequenced, rest was assumed based on date of sampling and national prevalence

[10] aOR: adjusted Odd Ratio
[11] p: p-value

Wiedenmann M, *et al. BMJ Open* 2023; 13:e072280. doi: 10.1136/bmjopen-2023-072280

Lorthe et al.<br>[59]	Cohort study	Switzerland	schools kindergartens crèches	59	10	3-7 yo, adults	n/r	Omicron		**Risk of infection AR (children vs. adults)**<br>44.1% (26/59) vs. 50% (5/10)<br><br>**AR (children by classes)**<br>1. 33.3% (4/12)<br>2. 15.4% (2/13)<br>3. 56.3% (9/16)<br>4. 61.1% (11/18)	**vaccination status:** n/r<br>**sequencing:** none

Supplementary table 12: Review question 4 - Risk of child/adolescent transmission when infected with a SARS-CoV-2 variant of concern (n=15)

Author	Study design	Country	Study setting	Number of index cases <20 yo[1]	Number of adult index cases	number of contacts of children/ adolescents	number of contacts of adults	Age groups	Mean/ Median age	VOC[2]	Risk of transmission compared to non-VOCs/other VOCs	Risk of transmission compared to adults	Comments
Lindstrom et al. [66]	cohort study	Norway	population	42<br><br>alpha: 14<br><br>non-VOC: 28	373<br><br>alpha: 131<br><br>non-VOC: 242	mean (SD[3]): 2.79 (1.63)	40-59yo: 1.72 (1.87)<br><br>20-39yo: 1.03 (1.36)<br><br>>60yo: 1.25 (1.65)	<20 yo, adults	n/r	Alpha	n/a	**Transmission risk SAR[4] (<20 yo vs. 40-59 yo)** ref. vs. 1.97 (1.08-3.87)<br><br>no significant difference to adults 20-39 yo, >60yo	**vaccination status:** n/r **sequencing:** primary cases sequenced, variants in contacts assumed based on date of sampling and national prevalence
Lyngse et al. [60]	cohort study	Denmark	households	145<br><br><10 yo: 54<br><br>10-19 yo: 91	663	383<br><br><10yo: 141<br><br>10-19yo: 242	1 336	≤10 yo, 10-20 yo, adults	n/r	Alpha	n/a	**Transmission risk AR[5] (≤20 yo vs. adults)** 32.4% (124/383) vs. 39.8% (532/1336)<br><br>**AR (≤10 yo vs. 10-19 yo vs. adults)** 45% (64/141) vs. 25% (60/242) vs. 39.8% (532/1336)	**vaccination status:** n/r **sequencing:** 75% sequenced, rest was assumed based on date of sampling and national prevalence

[1] yo: years old
[2] VOC: Variant Of Concern
[3] SD: Standard Deviation
[4] SAR: secondary attack rate
[5] AR: attack rate

Wiedenmann M, *et al. BMJ Open* 2023; 13:e072280. doi: 10.1136/bmjopen-2023-072280

Chudasama et al. [61]	cohort study	United Kingdom	households	6 011<br><br><10 yo: 1 289<br><br>10-19 yo: 4 722	57 382	n/r	n/r	<10 yo, 10-19 yo, adults	n/r	Alpha	n/a	**Transmission risk OR[6] (10-19 yo vs. <10 yo)** OR 0.83 (95%CI[7]: 0.69-0.99) (<10 yo: reference)<br><br>no difference in transmission risk compared to index cases aged 30-70 yo	**vaccination status:** n/r **sequencing:** all index cases sequenced, no information on contacts
Julin et al. [67]	cohort study	Norway	households	3<br><br>alpha: 2<br><br>non-VOC: 1	55<br><br>alpha: 16<br><br>non-VOC: 39	55<br><br>alpha: 16<br><br>non-VOC: 39	68<br><br>alpha: 20<br><br>non-VOC: 48	<20 yo, adults	median: 31 yo (IQR[8] 2-73)	Alpha	n/a	**Transmission risk (irrespective of VOC) Viral loads (mean, <20 yo vs. adults)** 2.09 log10 copies/ul RNA[9] vs. 2.98 log10 copies/ul RNA<br><br>**Length of testing positive (mean days, <20 yo vs. adults)** 11.3 (95%CI: 7.6–15.1) vs 16.4 (95%CI: 13.5–19.3), p[10] = 0.03	**vaccination status:** none vaccinated **sequencing:** all sequenced

[6] OR: Odd Ratio
[7] 95%CI: 95% Confidence Interval
[8] IQR: Interquartile Range
[9] RNA: Ribonucleic acid
[10] p: p-value

Wiedenmann M, et al. BMJ Open 2023; 13:e072280. doi: 10.1136/bmjopen-2023-072280

Loenenbach et al. [55]	cohort study	Germany	schools kindergartens crèches households	22	16	59	33	≤6 yo, adults	n/r	Alpha	n/a	**Transmission risk SAR[11] (≤6 yo vs. adults)** 39% (95%CI: 28-52) vs. 33% (95%CI: 20-50) RR[12] 1.17 (95%CI: 0.66-2.09)	**vaccination status:** n/r **sequencing:** n/r
Lorthe et al. [56]	cohort study	Switzerland	schools kindergartens crèches households	n/r	n/r	24	2	≤6 yo, adults	n/r	Alpha	n/a	**Transmission risk SAR (≤6 yo vs. adults)** 12.5% (3/24) vs. 50% (1/2)	**vaccination status:** n/r **sequencing:** all cases
Loss et al. [62]	case-control study	Germany	schools kindergartens crèches households	15<br><br>alpha: 9<br><br>non-VOC: 6	9<br><br>alpha: 4<br><br>non-VOC: 5	172<br><br>alpha: 114<br><br>non-VOC: 58	38	≤6 yo, adults	n/r	Alpha	**Transmission risk AR[13] (alpha vs. non-VOC)** 16.7% (19/114) vs. 8.6% (5/58)	**Transmission risk AR (≤6 yo vs. adults)** 16.7% (19/114) vs. 10.5% (4/38)	**vaccination status:** n/r **sequencing:** majority sequenced, rest was assumed based on date of sampling and national prevalence
Buchan et al. [63]	cohort study	Canada	households	715<br><br><10 yo alpha: 55 non-VOC: 195<br><br>10-19 yo alpha: 114 non-VOC: 351	4 902<br><br>alpha: 1 149 non-VOC: 3 753	n/r	n/r	<10 yo, 10-19 yo, adults	n/r	Alpha	**Transmission risk SAR (alpha vs. non-VOC)** aRR[14] (<10y o): 1.47 (95%CI: 0.81-2.65) aRR (10-19 yo): 1.30 (95%CI: 0.80-2.10)	n/a	**sequencing:** all cases either sequenced or screened positive for N501Y (marker for alpha variant) by PCR[15]

[11] SAR: Secondary Attack Rate
[12] RR: Risk Ratio
[13] AR: Attack Rate
[14] aRR: Adjusted Risk Ratio
[15] PCR: Polymerase Chain Reaction

Waltenburg et al. [18]	cohort study	USA[16]	households	36<br><br>alpha: 21<br><br>non-VOC: 10	n/a	111<br><br>alpha : 61<br><br>non-VOC : 39	n/a	<5 yo<br>5-11 yo<br>12-17 yo	median: 12 yo (IQR 7-15)	Alpha	**Transmission risk**<br>**SAR (alpha vs. non-VOC)**<br>55% (24/44) (95%CI: 39-70) vs. 46% (12/26) (95%CI: 27-67); OR 1.52 (95%CI: 0.51-4.53)<br><br>**SAR – alpha (≤11 yo vs. 12-17 yo)**<br>65% (11/17) (95%CI: 38-68) vs. 48% (13/27) (95%CI: 29-68); OR 1.63 (95%CI: 0.4-6.7)	n/a	**vaccination status:** 97% unvaccinated (147/151), 3% partially vaccinated (4/151) (2 primary cases and 2 secondary cases)<br><br>**sequencing:** majority of primary cases sequenced
Ratman et al. [68]	modeling study	USA	population	n/a	n/a	n/a	n/a	<10 yo, 10-19 yo	n/r	Alpha	**Transmission risk**<br>**R[17] (alpha vs. non-VOC)**<br><10yo: <1 vs. <<1<br>10-19 yo: ~1 vs. <<1	n/a	n/a
Somekh et al. [33]	cohort study	Israel	hospital inpatients hospital outpatients	72 426<br><br>alpha: 50 811<br><br>non-VOC: 21 615<br><br>(overall numbers and thus potential index cases)	n/r	alpha: 313 871<br><br>non-VOC: 156 521	n/r	<10 yo	n/r	Alpha	**Transmission risk**<br>**AR (alpha vs. non-VOC)**<br>15.7% (49257/313871) vs. 7.5% (11770/156521) RR: 2.24 (95%CI, 2.20-2.29), p < 0.001	n/a	**sequencing:** n/r

[16] USA: United States of America
[17] R: reproduction number

Allen et al. [64]	case-control study	United Kingdom	households	6 130<br><br>alpha: n/r<br><br>delta: n/r	17 928	n/r	n/r	<10 yo, 10-19 yo, adults	n/r	Alpha, Delta	n/a	**Trasmission risk aOR[18] (delta vs. alpha/ref. adults)**<br><10 yo: 0.98 (95%CI: 0.85-1.14)<br>10-19 yo: 0.71 (95%CI: 0.64-0.79)<br>(reference value 1.00 for 30-39 yo)	**vaccination status:** reported (but not in subanalysis comparing transmission risk in age groups)<br><br>**sequencing:** all index cases sequenced, nationwide 50% of all cases were sequenced at time of study
Trobajo-Sanmartin et al. [65]	cohort study	Spain	population	n/r	n/a	All contacts of <12 yo : 288<br><br>Alpha : 107<br><br>Delta : 181	n/a	<12yo, 12-17 yo, adults	n/r	Alpha, Delta	**Transmission risk SAR (alpha vs. delta)**<br><12 yo : 33% (35/107) vs. 23% (41/181); aRR 1 vs. 0.58 (95%CI: 0.28-1.23), p=0.155<br><br>higher risk of transmission for 12-39yo		**vaccination status:** none vaccinated<br><br>**sequencing:** none

[18] aOR: Adjusted Odd Ratio

Ng et al. [50]	cohort study	Singapore	households	n/r	n/r	472	18-29 yo : 1 467	≤11 yo, 12-17 yo, adults	median (contacts): 36 yo (IQR 26-51)	Delta	n/a	**Transmission risk SAR (≤11 yo vs. 18-29 yo)** 25.2% (88/349) vs. 9.4% (138/1457); aOR: 2.37 (95%CI:1.57-3.60), p<0.0001 **SAR (12-17 yo vs. 18-29 yo)** 17.9% (22/123) vs. 9.4% (138/1457) ; aOR 1.81 (95%CI: 0.91-3.6), p=0.089	**vaccination status:** >12 yo partially vaccinated (results adjusted for vaccination status) **sequencing:** partially sequenced
Lorthe et al. [59]	cohort study	Switzerland	schools kindergartens crèches, households	n/r	n/a	52	n/a	3-7 yo, adults	n/r	Omicron	**Transmission risk SAR** 48% (25/52)	n/a	**vaccination status:** n/r **sequencing:** none
