# Supplementary material for: SARS-CoV-2 variants of concern in children and adolescents with COVID-19: a systematic review
Source: BMJ Open. 2023 Oct 9;13(10):e072280. doi: 10.1136/bmjopen-2023-072280 (PMC10565293; doi:10.1136/bmjopen-2023-072280)
Supplement: Author's manuscript [file bmjopen-2023-072280.draft_revisions.pdf]

BMJ Open

BMJ Open is committed to open peer review. As part of this commitment we make the peer review history of every article we publish publicly available.

When an article is published we post the peer reviewers' comments and the authors' responses online. We also post the versions of the paper that were used during peer review. These are the versions that the peer review comments apply to.

The versions of the paper that follow are the versions that were submitted during the peer review process. They are not the versions of record or the final published versions. They should not be cited or distributed as the published version of this manuscript.

BMJ Open is an open access journal and the full, final, typeset and author-corrected version of record of the manuscript is available on our site with no access controls, subscription charges or pay-per-view fees (http://bmjopen.bmj.com).

If you have any questions on BMJ Open's open peer review process please email info.bmjopen@bmj.com

The role of SARS-CoV-2 variants of concern in children and adolescents with COVID-19: a systematic review

Journal:	*BMJ Open*
Manuscript ID	bmjopen-2023-072280
Article Type:	Original research
Date Submitted by the Author:	13-Feb-2023

Complete List of Authors:	Wiedenmann, Margarethe; Swiss Tropical and Public Health Institute; University of Basel<br>Ipekci, Aziz Mert; University of Bern, Institute of Social and Preventive Medicine<br>Araujo-Chaveron, Lucia; EHESP Rennes-Sorbonne Paris Cité; Institut Pasteur<br>Prajapati, Nirmala; Université Paris-Saclay; Institut national de la santé et de la recherche médicale<br>Lam, Yin Ting; University of Bern, Institute of Social and Preventive Medicine<br>Alam, Muhammad Irfanul ; EHESP Rennes-Sorbonne Paris Cité<br>L'Huillier, Arnaud ; HUG, Département de pédiatrie, gynécologie et obstétrique<br>Zhelyazkov, Ivan; The University of Sheffield<br>Heron, Leonie; University of Bern, Institute of Social and Preventive Medicine<br>Low, Nicola; University of Bern, Institute of Social and Preventive Medicine<br>Goutaki, Myrofora ; University of Bern, Institute of Social and Preventive Medicine

Keywords:	COVID-19, Public health < INFECTIOUS DISEASES, Systematic Review

SCHOLARONE™ Manuscripts

I, the Submitting Author has the right to grant and does grant on behalf of all authors of the Work (as defined in the below author licence), an exclusive licence and/or a non-exclusive licence for contributions from authors who are: i) UK Crown employees; ii) where BMJ has agreed a CC-BY licence shall apply, and/or iii) in accordance with the terms applicable for US Federal Government officers or employees acting as part of their official duties; on a worldwide, perpetual, irrevocable, royalty-free basis to BMJ Publishing Group Ltd ("BMJ") its licensees and where the relevant Journal is co-owned by BMJ to the co-owners of the Journal, to publish the Work in this journal and any other BMJ products and to exploit all rights, as set out in our licence.

The Submitting Author accepts and understands that any supply made under these terms is made by BMJ to the Submitting Author unless you are acting as an employee on behalf of your employer or a postgraduate student of an affiliated institution which is paying any applicable article publishing charge ("APC") for Open Access articles. Where the Submitting Author wishes to make the Work available on an Open Access basis (and intends to pay the relevant APC), the terms of reuse of such Open Access shall be governed by a Creative Commons licence – details of these licences and which Creative Commons licence will apply to this Work are set out in our licence referred to above.

Other than as permitted in any relevant BMJ Author's Self Archiving Policies, I confirm this Work has not been accepted for publication elsewhere, is not being considered for publication elsewhere and does not duplicate material already published. I confirm all authors consent to publication of this Work and authorise the granting of this licence.

For peer review only

The role of SARS-CoV-2 variants of concern in children and adolescents with COVID-19: a systematic review

Margarethe Wiedenmann[1,2], Aziz Mert Ipekci[3], Lucia Araujo Chaveron[4,5], Nirmala Prajapati[6,7], Yin Ting Lam[3], Muhammad Irfanul Alam[4], Arnaud G. L'Huillier[8], Ivan Zhelyazkov[9], Leonie Heron[3], Nicola Low[3*], Myrofora Goutaki[3*]

[1] Swiss Tropical and Public Health Institute, Basel, Switzerland
[2] University of Basel, Basel, Switzerland
[3] Institute of Social and Preventive Medicine (ISPM), University of Bern, Bern, Switzerland
[4] EHESP French School of Public Health, Paris/Rennes, France
[5] Institute Pasteur, Paris, France
[6] Université Paris-Saclay, Paris, France
[7] Institut national de la santé et de la recherche médicale (INSERM), Paris, France
[8] Geneva University Hospitals and Faculty of Medicine, Geneva, Switzerland
[9] University of Sheffield, Sheffield, United Kingdom
[*] These authors have contributed equally to this work and share last authorship

Corresponding author: margarethe.wiedenmann@swisstph.ch

For peer review only - http://bmjopen.bmj.com/site/about/guidelines.xhtml

Abstract

Objectives: Infections by SARS-CoV-2 variants of concern (VOCs) might affect children and adolescents differently than earlier viral lineages. We aimed to address five questions about SARS-CoV-2 VOC infections in children and adolescents: i) symptoms and severity, ii) risk factors for severe disease, iii) the risk of becoming infected, iv) the risk of transmission and v) long-term consequences following a VOC infection.

Methods: We carried out a systematic review. We searched the COVID-19 Open Access Project database up to 1 March 2022 and PubMed up to 9 May 2022 for observational epidemiological studies about alpha, beta, gamma, delta and omicron VOCs among 0 to 18 year olds. We synthesised data for each question descriptively and assessed the risks of bias at the outcome level.

Results: We included 53 articles, of which 47% were from high-income countries and none were from low-income countries, according to World Bank categories. Most children with any VOC infection presented with mild disease, with more severe disease being described with the delta or the gamma VOC. Diabetes and obesity were reported as risk factors for severe disease during the whole pandemic period. The risk of becoming infected with a SARS-CoV-2 VOC seemed to increase with age, while in daycare settings the risk of onward transmission of VOCs was higher for younger than older children or at least partially vaccinated adults. Long-term symptoms or signs following an infection with a VOC were described in <5% of children and adolescents.

Conclusion: Overall patterns of SARS-CoV-2 VOC infections in children and adolescents are similar to those of earlier lineages. Comparisons between different pandemic periods, countries and age groups should be improved with complete reporting of relevant contextual factors, including VOCs, vaccination status of study participants and the risk of exposure of the population to SARS-CoV-2.

PROSPERO registration number: CRD42022295207

For peer review only - http://bmjopen.bmj.com/site/about/guidelines.xhtml

Key messages

What is already known on this topic

SARS-CoV-2 variants of concern (VOCs) might affect children and adolescents differently from earlier viral lineages.

What this study adds

Children and adolescents are susceptible to SARS-CoV-2 VOC infection, though they mostly experience mild disease, and can transmit the VOCs. More severe disease was described with the delta or the gamma VOC but comparison within paediatric age groups as well as to adults is hindered by the lack of reporting of contextual factors such as the vaccination status of these groups.

How this study might affect research practice or policy

The applicability of our findings about clinical presentations, susceptibility and transmissibility of SARS-CoV-2 VOCs is limited by an absence of research from low- and middle-income settings. As new VOCs continue to emerge, new studies are needed globally, with methods and results reported in ways that allow comparison between different VOCs and age groups.

For peer review only

For peer review only - http://bmjopen.bmj.com/site/about/guidelines.xhtml

Introduction

The role of children and adolescents in the pandemic of severe acute respiratory syndrome coronavirus 2 (SARS-CoV-2), the cause of coronavirus disease 2019 (COVID-19), remains an important topic of discussion. SARS-CoV-2 variants of concern (VOCs) have emerged since late 2020, with the alpha variant (previously referred to as B.1.1.7) being the first. These lineages possess one or more mutations, which are associated with different phenotypic properties, such as changes in the severity of disease, increased transmissibility, or the ability to evade infection- or vaccine-induced immunity.[1] The circulation of alpha, beta, gamma, delta, and omicron variants has led to large waves of COVID-19 in different countries.[2] At around the same time as the first VOCs emerged and spread, vaccines that gave protection against infection and severe disease caused by the original SARS-CoV-2 lineage were introduced in adults.[3,4] Vaccines were first licensed for 16-18 year-olds in spring 2021 and later for children <16 years old, but vaccination availability and uptake varied by country.[5] Also, preventive interventions introduced at the population level have been relaxed in many countries. The number of children and adolescents testing positive for SARS-CoV-2 is much higher than in the pre-VOC period, so a re-evaluation of the role of children and adolescents in the SARS-CoV-2 pandemic is warranted.

The objectives of this study were to address five research topics about SARS-CoV-2 VOC infections in children and adolescents: 1. What are the symptoms caused by SARS-CoV-2 VOCs in children and adolescents, and do they differ from those of SARS-CoV-2 wild type infections? 2. What are the risk factors for severe COVID-19 in children and adolescents infected with a SARS-CoV-2 VOC? 3. Does the risk of infection with a SARS-CoV-2 VOC differ between children, adolescents and adults who have not received a COVID-19 vaccine? 4. Does the risk of transmission of a SARS-CoV-2 VOC differ between children, adolescents and adults who have not received a COVID-19 vaccine? 5. Does the risk of developing long-term effects (long-COVID) after infection with a SARS-CoV-2 VOC differ between children, adolescents and adults who have not received a COVID-19 vaccine?

For peer review only - http://bmjopen.bmj.com/site/about/guidelines.xhtml

Methods

Design

We registered a protocol for this review in the International Prospective Register of Systematic Reviews (PROSPERO) (registration ID: CRD42022295207).[6] We have published an earlier version of this review, covering the period before the circulation of VOCs.[7] We report the study according to the Preferred Reporting Items for Systematic Review and Meta-Analyses guidelines (supplementary table 1).[8]

Search strategy

We searched: 1) the COVID-19 Open Access Project (COAP) database, which aggregates COVID-19 research from PubMed, EMBASE, PsycINFO, bioRxiv and medRxiv from 1 January 2020 to 1 March 2022 and 2) PubMed using MeSH terms from 1 August 2020 to 30 January 2022. [9] Owing to large numbers of records that did not fulfil the inclusion criteria, we revised the PubMed search strategy, including each VOC, identified by their Greek letter and Pangolin lineage terminology, and conducted two new searches between 31 January and 7 March and between 8 March and 9 May 2022. We also searched reference lists of systematic reviews and included studies, and we included studies suggested by experts in the field, but not identified by our search. Full search terms are included in the supplementary note 1.

Eligibility and screening

Observational epidemiological studies (case series with more than one case, cross-sectional studies, case-control studies and cohort studies) and modelling studies were eligible for inclusion. We did not apply any geographical or language restrictions to the search, but we only read the full text of papers written in languages spoken fluently by the review team (English, German, French, Greek, Italian, Spanish or Turkish). Identified titles and abstracts were screened by one member from an international team of ten reviewers and a second member verified the selection. We followed the same approach for full-text screening. A third reviewer resolved disagreements.

Data extraction

We developed, piloted and revised a data extraction template in Microsoft Excel at the beginning of the study. One reviewer extracted data from each included study

For peer review only - http://bmjopen.bmj.com/site/about/guidelines.xhtml

and a second reviewer verified the extraction. Extracted data included information on the publication characteristics, study design, study setting, study population overall and those younger than 20 years old, age groups, prevalent VOC(s) at the time of study, and outcomes for each relevant review question.

Risk of bias assessment

We assessed the risk of bias at the outcome level using published tools for cohort and case control studies.[10,11] The credibility and relevance of modelling studies was evaluated using a published checklist.[12] To help with interpretation of the findings, we also recorded whether the authors of included studies reported on the stage of the SARS-CoV-2 pandemic, on any existing control measures, or on the vaccination status of the study participants at the time of the study.

Data synthesis

We synthesised the information from the included studies descriptively for each question. We tabulated summaries of the data extracted, including the number of studies identified for each VOC, the age groups studied and the outcomes reported.

The presence of a VOC was determined by genetic sequencing or by assumptions based on authors' reporting, the dominant lineage at the time of testing, and the study location. Definitions of disease severity differed between studies. To allow comparisons, we applied the following categorisation: mild or asymptomatic disease (patients were not hospitalised), moderate disease (patients were hospitalised, but did not require intensive care), severe disease (patients were hospitalised and intensive care was necessary). If we did not have sufficient information to apply these categories, we used the authors' definitions and reported these in the text. Where possible, we presented the results separately for different age groups: neonates and infants 0-1 years old; young children 1-4 years old; older children 5-9 years old; younger and older adolescents 10-20 years old. If the intended age groups were not reported, we used the groups reported by study authors. We categorised the country in which the study was conducted according to World Bank income groups as a simplified proxy for the level of government resources spent on health services and public health surveillance activities.[13] We report our findings descriptively. There were too few studies that reported quantitative findings in a comparable way to conduct meta-analysis.

For peer review only - http://bmjopen.bmj.com/site/about/guidelines.xhtml

Patient and public involvement

This research was done without patient or public involvement.

Results

We screened 2407 articles published up to 09 May 2022, 53 of which were included (figure 1 and supplementary figures 1-4). According to World Bank data, forty-seven (89%) articles were from 14 high-income countries, six (11%) were from five upper middle-income countries and none was from a lower middle-income or low-income country (table 1 and figure 2).

Of the 53 studies included in the review, 44 (83%) were cohort studies, 4 (7.5%) were cross-sectional studies, 3 (5.7%) were case-control studies, 1 (1.9%) was a modelling study and 1 (1.9%) was a case-series (table 1). Some included studies compared different variants and others different age groups, infected with the same variant.

Most studies were judged to be at moderate risk of bias in most domains. There were high risks of bias in a few domains especially regarding matching of exposed and unexposed cohorts as well as the assessment of prognostic factors (irrespective of the research question they addressed) as most studies did not control for possible confounding factors (supplementary tables 2-6).

Table 1: Characteristics of included studies

Characteristics	total	alpha	beta	gamma	delta	omicron
Number of studies, n*	53	27	2	2	27	12
Review question addressed, n*	35	13	2	2	21	11
- Review question no. 1	2	1	1	1	2	2
- review question no. 2	15	10	-	-	4	1
- review question no. 3	15	13	-	-	3	1
- review question no. 4	-	2	1	1	2	-
- review question no. 5						
Study design						
- cohort	44	21	2	2	24	9
- case-control	3	3	-	-	-	-
- cross-sectional	4	2	-	-	3	2
- modelling	1	1	-	-	-	-
- case series	1	-	-	-	-	1
Income category according to world bank categories, n						

For peer review only - http://bmjopen.bmj.com/site/about/guidelines.xhtml

- high income	47	26	1	1	23	11
- upper middle income	6	1	1	1	4	1
- lower middle income	-	-	-	-	-	-
- low income	-	-	-	-	-	-

* The total number of studies included is 53. The added number for each VOC is more than 53 as some studies reported on more than just one VOC.

Review question 1: Symptoms and severity of disease among children and adolescents infected with VOCs

Symptoms: Symptoms experienced by children and adolescents infected with a VOC were presented in 18 studies involving the alpha (n=7), delta (n=6), omicron (n=4) or gamma variant (n=1) (supplementary tables 7-10). Children and adolescents experienced a wide range of symptoms. We did not identify any symptoms consistently associated with a specific VOC or whether symptom frequencies with VOC infections differed from the pre-VOC period. One study reported on croup in children and adolescents infected with the omicron variant and found an increase in croup cases in hospital inpatients and outpatients compared with the pre-omicron period (median weekly croup cases (omicron vs. pre-omicron): 11 [IQR 2–17] vs. 0 (IQR 0-0), $p < 0.001$) (supplementary table 9).[37] We did not find any studies investigating symptoms in children infected with the beta variant. A detailed comparison between studies was not possible owing to differences in study populations (community or health care setting), symptoms recorded and age groups reported.[14-18,20,22-24,27,31-33,35,36,38-40]

Severity: Children and adolescents mostly experienced mild disease when infected with a SARS-CoV-2 VOC. More severe infections were described with the gamma variant compared to pre-VOC variants. On the other hand, infections with the omicron variant were less severe compared to delta variant infections

Paediatric disease severity associated with SARS-CoV-2 VOC infections was described in 23 studies (supplementary tables 7-10). The alpha variant did not appear to result in more severe COVID-19 disease than disease caused by non-VOCs in children and adolescents in five studies.[17-21] In two studies reporting on paediatric inpatients and outpatients one from Brazil and one from the USA, the overall hospitalisation rate as well as the ICU admission rate of children infected with the gamma variant was higher than for non-VOCs (Gamma vs. non-VOC: 28.3% vs. 24.9%.[47], Gamma vs. non-VOC: 5.2% vs. 1.2%[48]).[47,48] Severity of delta variant

For peer review only - http://bmjopen.bmj.com/site/about/guidelines.xhtml

infection was compared with the pre-delta period (including periods where previous VOCs where predominant) in eight studies but the reported data were inconsistent, and differences in disease severity could not be assessed.[26,28-30,32-35] Studies of omicron variant infection described less severe disease than infection with the delta variant in children and adolescents <18 years old, based on data from nine studies from South Africa, Qatar and the USA.[36,39-46]

One study from Denmark analysed the symptoms and severity of disease experienced by children and adolescents younger than 17 years old, hospitalised for multi-inflammatory syndrome in children (MIS-C) during the delta and non-VOC predominant periods. The range of symptoms was similar in both periods, as was the proportion of children admitted to ICU (delta, 55% (28/51) vs. non-VOC 52% (12/23)).[25]

We did not find any studies showing an association between the SARS-CoV-2 omicron lineages and the global outbreak of paediatric hepatitis of unknown origin, which has been attributed to adenoviruses in spring 2022.[49]

No studies reported on how symptoms or severity differed between different paediatric age groups or compared children and adolescents infected with a VOC to adults infected with the same variant.

Review question 2: Risk factors for severe COVID-19 in children and adolescents infected with a SARS-CoV-2 VOC

We found one article that used data from the National COVID-19 database in Qatar and compared risk factors for severe COVID-19 in children and adolescents infected with the omicron and delta variants. The study showed that children and adolescents <18 years old with ≥1 comorbidity (mostly chronic lung disease) were more likely to have moderate or severe disease than those without comorbidities if they were infected with the omicron lineage (aOR 95%CI 3.2, 1.1-9.0), but not if they were infected with the delta lineage.[39] One paper reported on risk factors for severe COVID-19 disease in children 5-11 years old during the whole pandemic period, including the time when the omicron variant became predominant. The study assessed several possible risk factors and found evidence of an increased risk of severe disease in children with diabetes or obesity.[45]

For peer review only - http://bmjopen.bmj.com/site/about/guidelines.xhtml

Review question 3: Risk of becoming infected with a SARS-CoV-2 VOC

The risk of becoming infected with a SARS-CoV-2 VOC was analysed in time periods when the alpha, delta or omicron variants were predominant in 15 studies (supplementary table 11). Immunity due to previous infection or vaccination appeared to be a relevant factor in determining the risk of infection but these factors were poorly reported in the included studies. Some studies (4/15) summarised the vaccination status of the entire study sample, which ranged from an undefined proportion of partially vaccinated individuals to 70% fully vaccinated, without analysing it at the individual level for adults and children and adolescents.[17,56-58]

Six studies evaluated data from alpha variant outbreaks in daycare centres and compared the risk of infection between children and adults.[14,18,50- 53] They generally showed a lower attack rate for infection with the alpha variant ranging from 0% to 27% for young children (mostly ≤6 years old) than for adults (0%-71%). However, there were large variations in attack rates between different facilities or classes at the same facility. The study participants' vaccination status was not reported in any of these studies.

One study reported the attack rates of children by age group. The attack rates for young children (≤4 years old) decreased with increasing age from 44% to 4%, with the highest rate for infants <1 year old.[14] The attack rates of older children (10-19 years old) and adults (30-39 years old) were similar in one study from Qatar, but the vaccination status was not reported.[54]

In one study analysing household transmission of the alpha variant in the USA, the risk of infection was high in both younger children ≤11 years old (78%, 95%CI: 51-76%) and older children and adolescents 12-17 years old (56%, 95%CI: 40-72%) with overlapping CIs.[17] Compared with the pre-VOC period, a similar or lower risk of infection was reported in three household or school studies for children and adolescents of all age groups when the alpha variant was predominant.[17,18,50] However, in two studies from Israel, including a large hospital study, a higher risk of infection for the alpha variant than for non-VOCs was described for all paediatric age groups.[16,19] Data on the risk of infection during the delta predominant time period analysed in four studies comparing children and adolescents to adults were inconclusive.[55-58] Two of these four studies, one cohort study from Singapore and

For peer review only - http://bmjopen.bmj.com/site/about/guidelines.xhtml

one from the UK found the risk of infection to be higher for younger children ≤11years old/school-aged children 5-18 years old than for adults who were either completely or partially vaccinated.[57,58] . One study analysed the risk of infection in daycare centres during the Omicron predominant time period and found it to be slightly lower for young children 3-7 years old (44%) than for adults (50%). As described, during the alpha-predominant period large variations in attack rates (15.4%-61.1%) were observed between different school classes.[59]

Review question 4: Risk of SARS-CoV-2 transmission from children and adolescent infected with a VOC

The risk of SARS-CoV-2 transmission was evaluated in time periods when alpha, delta or omicron variants were predominant in 15 studies (supplementary table 12).

Overall, transmission risk with a VOC was reported to be higher for younger children in most studies. A higher risk of onward SARS-CoV-2 alpha variant transmission in household settings from younger children <10 years old than from older children 10-19 years old and adults was found in four studies.[52,61,62,64] These studies did not report on whether the participating adults or older children were vaccinated. One household study from the USA found comparable transmission of alpha variant infections from children ≤11 years old compared with adolescents 12-17 years old (97% of whom were unvaccinated) (OR 1.37, 95% CI 0.61, 3.04).[17] The risk of transmission from children <10 years old with an alpha variant infection was found to be higher than from children with a pre-VOC or non-VOC infection in three studies.[19,64,65] One study found the transmission risk from 10-19 year olds with an alpha variant infection to be similar to that of pre-VOC infection.[65] Comparing the risk of onward transmission between age groups for participants infected with either the alpha or delta variant, the risk was similar for younger children <12 years old and lower for older children and adolescent 10-19 years old, compared with 30-39 year old adults.[67,68] One household transmission study from Singapore found the transmission risk of younger children ≤11 years old infected with the delta variant to be higher (25%) than the transmission risk of young adults 18-29 years old who were at least partially vaccinated (9%).[56] One study analysed the risk of onward transmission of young children ≤6 years old with an omicron variant infection in a daycare setting and found it to be lower (3/24, 12.5%) than the risk of onward transmission from adults (1/2, 50%).[59]

For peer review only - http://bmjopen.bmj.com/site/about/guidelines.xhtml

Review question 5: Risk of long-term symptoms or signs following an infection with a SARS-CoV-2 VOC

Few children reported long-term symptoms or signs of infection. The risk for children and adolescents experiencing symptoms longer than 28 days was similar for children infected with the alpha (12/694, 1.73%) and the delta lineage (15/706, 2.12%) in the UK, and was similar for younger (5-11 years old) compared to older (12-17 years old) children infected with the same variant.[32] For those children with longer illness duration, no new symptoms developed after day 28. A study comparing Danish children who tested positive for SARS-CoV-2 with a reference cohort of children who tested negative during the overall pandemic period (alpha and delta variant predominant times included) found that 0.12% (95%CI: 0.09%-0.15%) of children experienced symptoms for more than 28 days (58 of 48948 children).[33]

Discussion

Children and adolescents (<20 years old) with a SARS-CoV-2 VOC infection mostly experienced mild disease and the symptoms experienced did not vary widely between VOCs. Compared to non-VOC and other VOC induced infections more severe infection was observed with either the delta or the gamma SARS-CoV-2 variant. Compared to otherwise healthy peers, children and adolescents with comorbidities appeared to have an increased risk of infection by omicron, but not delta, and diabetes and obesity were reported as risk factors for severe infection during the whole pandemic period. The studies reviewed suggest that the risk of SARS-CoV-2 infection in daycare centres was lower overall for younger children than for adults, while the risk compared to adults decreased with increasing age of the children. The transmission risk of SARS-CoV-2 from younger children infected with a VOC seemed to have been higher than that of older children and adults as well. The proportion of children and adolescents reporting symptoms more than 28 days after a SARS-CoV-2 VOC infection was low. Interpretation and generalisation of the evidence was limited by inconsistent reporting of age groups and by inadequate reporting of vaccination and other control measures in included studies.

Strength and limitations

We used a systematic approach to search different databases, covering the period of VOC circulation that includes the first omicron variants (BA.1 and BA.2) in 2022. We

For peer review only - http://bmjopen.bmj.com/site/about/guidelines.xhtml

included a broad selection of study designs in various languages and screened reference lists of included studies. Our search strategy might have missed relevant studies, although we only excluded three records for language reasons. A limitation of the review is that misclassification of the variant could have occurred as only a minority of study samples from all cases was sequenced in several studies and we relied on dates during which a variant was dominant. Non-differential misclassification would reduce the magnitude of associations with VOC infection status.

Interpretation and implications of findings

The severity of SARS-CoV-2 infections, the risk of becoming infected and the risk of onward transmission depend on both intrinsic factors associated with the person (e.g. host immunity) and the pathogen (e.g. variant transmissibility, virulence, immune evasion) and extrinsic factors (e.g. study setting, COVID-19 at the population level, vaccination status of study participants, preventive measures in place). Differentiation between the contributions of intrinsic and extrinsic factors in studies included in this review was challenging because frequent deficiencies in reporting of extrinsic factors hindered comparisons and interpretation of outcomes from different regions or different pandemic periods, even from the same country. All studies were conducted in high or upper-middle income countries, which might affect the generalisability of the review findings to children in settings with less-resourced health systems, lower vaccine coverage and different patterns of comorbidity and co-infection. We assessed the risk of bias using published tools for cohort and case-control studies but found that the selection bias was hard to assess, especially for studies addressing the symptoms and severity of disease in hospitalised populations, since reporting of indications for hospitalisation (especially for children) varied between countries and between different stages of the pandemic.[10,11]

Risk of exposure to, and transmission of, SARS-CoV-2

The risk of exposure to SARS-CoV-2 has a direct effect on the risk of becoming infected, which influences the risk of onward transmission. Younger children are more dependent on adult care takers, who were most likely to be the index case, than older children and adolescents. This increased risk of exposure to infected

For peer review only - http://bmjopen.bmj.com/site/about/guidelines.xhtml

adults might explain their higher risk of becoming infected and for onward transmission compared to older children and adolescents. The risk of exposure to SARS-CoV-2 also influences the interpretation of the data on disease severity in children and adolescents with chronic diseases. We found an increased risk of severe disease for children with diabetes or obesity, but not for children and adolescents with respiratory conditions, except if they were infected with omicron.[45] As high-risk populations have been known to take additional measures to stay safe since the beginning of the pandemic, these findings could, however, also be partly explained by more intense shielding.[69]

Vaccination status

Vaccines protecting people from severe COVID-19 were first licensed for adults at the end of 2020 at around the same time as the alpha VOC became widespread. These vaccines also reduced the risk of becoming infected with SARS-COV-2 as well as the risk of transmitting it.[4,70,71] At more recent stages of the pandemic, such as when the omicron lineage first emerged, the original COVID-19 vaccine from Pfizer (Comirnaty) had become licensed for children ≥5 years old and offered protection from severe disease and at least partial protection from becoming infected and potentially from onward transmission.[72-74] Vaccine uptake, however, remained low for children in most countries. More importantly, a majority of countries with less financial resources and restricted access to health services have not received a sufficient number of vaccine doses to ensure high vaccination coverage even for healthcare workers and vulnerable elderly populations let alone for children and adolescents.[75] Irrespective of the prevalent SARS-CoV-2 lineage this low vaccine uptake and accessibility increased the risk of children and adolescents not only becoming infected themselves but of transmitting the disease to other children and adults. Vaccines for children <5 years old have only recently been licensed in countries with sufficient financial resources.[76,77] In addition to the increased care needs of younger children and thus their higher risk of potential exposure to COVID-19 infected adults, their higher risk of infection and onward transmission compared to older children and adolescents might also be explained by a difference in vaccination status.

For peer review only - http://bmjopen.bmj.com/site/about/guidelines.xhtml

Recommendations for hospitalisation

Recommendations for hospitalisation (as a sign of at least moderate disease) and treatment guidelines for children and adolescents infected with COVID-19 were not standardised, either at the country level or internationally, have changed over the course of the pandemic and might differ between different hospitals within and between countries. These variations made it impossible to compare the prevalence of moderate and severe COVID-19 disease both between countries or between different pandemic periods in the same country, irrespective of the prevalent SARS-CoV-2 lineage.

Conclusion

Children are susceptible to infection with a SARS-CoV-2 VOC and can transmit them. Overall patterns of SARS-CoV-2 caused by VOCs among children and adolescents appear similar to those observed in the pre-VOC period. Identification of characteristics associated with specific VOCs or VOC infections in certain age groups is challenged by the frequent lack of data reporting of contextual factors, including the vaccination status of included participants and levels of prevalent SARS-CoV-2. Further studies assessing age-related differences in the risk of infection, the risk of transmission as well as risk factors for severe disease and long-term symptoms or signs after SARS-CoV-2 VOC infection are needed globally. These studies should report the vaccination status of participating adults and children as well as any measures affecting transmission (exposure type and intensity) and hospitalisation (local guidelines) during the study period. The available data are from studies in higher income settings, where the population is not only assumed to have easier access to health services but where resources for surveillance and research are also more readily available than in low- and middle-income settings. Owing to a lack of access to vaccines and generally low vaccine coverage, children account for a largely unvaccinated part of the population, especially in low- and middle-income countries. Local data acquisition and dissemination about SARS-CoV-2 in children and adolescents remain important both for affected countries and worldwide as SARS-CoV-2 continues to mutate and spread.

For peer review only - http://bmjopen.bmj.com/site/about/guidelines.xhtml

For peer review only

Acknowledgements

We would like to thank all members of WHO's Maternal, Newborn, Child and Adolescent Health and Ageing department, who contributed to the development of this review: Kathleen Strong, Theresa Diaz, Anshu Banerjee, Maria van Kerkhove, Abdi Mahamud, Valentina Baltag, Judith Ann Mandelbaum-Schmid.

Contributorship statement

MW, NL and MG conceived and designed the review. MW, LH and MG performed the literature search. MW, ML, MI, LAC, NP, YTL, MIA, ALH, IZ, LH and MG screened the articles for relevance and extracted the data. MW, MG and NL adjudicated as third reviewers in case of disagreements during the screening and extraction. MW, MI, LAC and MG synthesised the data. MW, MI, LAC, LH, NP, YTL, MIA, ALH, NL and MG all discussed the interpretation of the findings. MW and MG wrote the first draft of the manuscript. MI, LAC, NP, YTL, MIA, ALH, LH and NL all reviewed and revised the manuscript and approved the final version.

Competing interests

MW, MI, LAC, NP, YTL, MIA, ALH, IZ, LH, MG and NL declare no conflict of interest.

Funding

MW received funding from the World Health Organization (purchase order: 202807927, award grand number: n/a). MIA received the Erasmus Mundus Excellence Scholarship for a European Master of Public Health (2018-2020, award grand number: n/a). He also received subsistence allowance from WHO HQ during an internship and is now receiving a salary from WHO Bangladesh. NL received funds from the Swiss National Science Foundation (project number 176233) and European Union Horizon 2020 research and innovation programme - project EpiPose (grant agreement number 101003688). MG received grants from the Swiss National Science Foundation Ambizione fellowship (PZ00P3_185923) as well as from the Fondation Johanna Dürmüller-Bol (award grand number: n/a).

Data availability and data sharing statement

No datasets were generated. Data sharing is thus not applicable.

Ethics approval statement

This study does neither involve human participants nor animal subjects. Ethics approval by an ethics committee/institutional board was consequently not necessary.

For peer review only - http://bmjopen.bmj.com/site/about/guidelines.xhtml

References

1. World Health Organization. Tracking SARS-CoV-2 variants 2022 [updated May 25 2022. Available from: https://www.who.int/activities/tracking-SARS-CoV-2-variants.

2. Thakur V, Bhola S, Thakur P, Patel SKS, Kulshrestha S, Ratho RK, et al. Waves and variants of SARS-CoV-2: understanding the causes and effect of the COVID-19 catastrophe. Infection. 2022;50(2):309-25.

3. Ssentongo P, Ssentongo AE, Voleti N, Groff D, Sun A, Ba DM, et al. SARS-CoV-2 vaccine effectiveness against infection, symptomatic and severe COVID-19: a systematic review and meta-analysis. BMC Infect Dis. 2022;22(1):439. doi: 10.1186/s12879-022-07418-y.

4. Lauring AS, Tenforde MW, Chappell JD, Gaglani M, Ginde AA, McNeal T, et al. Clinical severity of, and effectiveness of mRNA vaccines against, covid-19 from omicron, delta, and alpha SARS-CoV-2 variants in the United States: prospective observational study. BMJ. 2022;376:e069761. doi: 10.1136/bmj-2021-069761.

5. Hunter DJ, Abdool Karim SS, Baden LR, Farrar JJ, Hamel MB, Longo DL, et al. Addressing Vaccine Inequity - Covid-19 Vaccines as a Global Public Good. N Engl J Med. 2022;386(12):1176-9.

6. Wiedenmann M, Goutaki M, Low N, Stringhini S, l'Huillier A. The role of children/adolescents in the SARS-CoV-2 pandemic with regards to variants of concern: a rapid review PROSPERO 2022 CRD42022295207 2022 [Available from: https://www.crd.york.ac.uk/prospero/display_record.php?RecordID=295207.

7. Wiedenmann M, Goutaki M, Keiser O, Stringhini S, Tanner M, Low N. The role of children and adolescents in the SARS-CoV-2 pandemic: a rapid review. Swiss Med Wkly. 2021;151:w30058.

8. PRISMA, Transparent Reporting of Systematic Reviews and Meta-Analyses. PRISMA Checklist 2021 [Available from: https://prisma-statement.org/PRISMAStatement/Checklist.]

9. COVID-19 Open Access Project. (2021). *Living Evidence on COVID-19*. Retrieved from https://ispmbern.github.io/covid-19/living-review/

10. CLARITY Group at McMaster University. Tool to Assess Risk of Bias in Cohort Studies [Available from: https://www.evidencepartners.com/wp-content/uploads/2021/03/Tool-to-Assess-Risk-of-Bias-in-Cohort-Studies-DistillerSR.pdf.

11. CLARITY Group at McMaster University. Tool to Assess Risk of Bias in Case Control Studies [Available from: https://www.evidencepartners.com/wp-content/uploads/2021/03/Tool-to-Assess-Risk-of-Bias-in-Case-Control-Studies-DistillerSR.pdf.

12. Jaime Caro J, Eddy DM, Kan H, Kaltz C, Patel B, Eldessouki R, et al. Questionnaire to assess relevance and credibility of modeling studies for informing health care decision making: an ISPOR-AMCP-NPC Good Practice Task Force report. Value Health. 2014;17(2):174-82. doi: 10.1016/j.jval.2014.01.003.

For peer review only

For peer review only - http://bmjopen.bmj.com/site/about/guidelines.xhtml

13. The World Bank. Data for High income, Low income, Lower middle income, Upper middle income [Available from: https://data.worldbank.org/?locations=XD-XM-XN-XT.

14. Roberts RJ, Brooker A, Lakshman R. Outbreak of SARS-CoV-2 in a Children's Nursery in the United Kingdom. Pediatr Infect Dis J. 2021;40(12):e455-e8. doi: 10.1097/INF.0000000000003362.

15. Ulyte A, Radtke T, Abela IA, Haile SR, Ammann P, Berger C, et al. Evolution of SARS-CoV-2 seroprevalence and clusters in school children from June 2020 to April 2021: prospective cohort study Ciao Corona. Swiss Med Wkly. 2021;151:w30092. doi: 10.4414/smw.2021.w30092.

16. Somekh I, Sharabi A, Dory Y, Simoes EAF, Somekh E. Intrafamilial Spread and Altered Symptomatology of SARS-CoV-2, During Predominant Circulation of Lineage B.1.1.7 Variant in Israel. Pediatr Infect Dis J. 2021;40(8):e310-e1. doi: 10.1097/INF.0000000000003167.

17. Waltenburg MA, Whaley MJ, Chancey RJ, Donnelly MAP, Chuey MR, Soto R, et al. Household Transmission and Symptomology of Severe Acute Respiratory Syndrome Coronavirus 2 Alpha Variant Among Children-California and Colorado, 2021. J Pediatr. 2022.

18. Nakel J, Robitaille A, Gunther T, Rosenau L, Czech-Sioli M, Plenge-Bonig A, et al. Comparing susceptibility and contagiousness in concurrent outbreaks with a non-VOC and the VOC SARS-CoV-2 variant B.1.1.7 in daycare centers in Hamburg, Germany. Int J Hyg Environ Health. 2022;240:113928. doi: 10.1016/j.ijheh.2022.113928.

19. Somekh I, Stein M, Karakis I, Simoes EAF, Somekh E. Characteristics of SARS-CoV-2 Infections in Israeli Children During the Circulation of Different SARS-CoV-2 Variants. JAMA Netw Open. 2021;4(9):e2124343. doi: 10.1001/jamanetworkopen.2021.24343.

20. Swann OV, Pollock L, Holden KA, Munro APS, Bennett A, Williams TC, et al. Comparison of UK paediatric SARS-CoV-2 admissions across the first and second pandemic waves. Pediatr Res. 2022. doi: 10.1038/s41390-022-02052-5.

21. Brookman S, Cook J, Zucherman M, Broughton S, Harman K, Gupta A. Effect of the new SARS-CoV-2 variant B.1.1.7 on children and young people. Lancet Child Adolesc Health. 2021;5(4):e9-e10. doi: 10.1016/S2352-4642(21)00030-4.

22. Stewart DJ, Mudalige NL, Johnson M, Shroff R, du Pre P, Stojanovic J. Acute kidney injury in paediatric inflammatory multisystem syndrome temporally associated with SARS-CoV-2 (PIMS-TS) is not associated with progression to chronic kidney disease. Arch Dis Child. 2022;107(3):e21. doi: 10.1136/archdischild-2021-322866.

23. Li H, Lin H, Chen X, Li H, Li H, Lin S, et al. Unvaccinated Children Are an Important Link in the Transmission of SARS-CoV-2 Delta Variant (B1.617.2): Comparative Clinical Evidence From a Recent Community Surge. Front Cell Infect Microbiol. 2022;12:814782. doi: 10.3389/fcimb.2022.814782.

24. Hao J, Hu XC, Fan MX, Chen J, Cheng QR, Li Z, et al. Analysis of clinical characteristics of 66 pediatric patients with B.1.617.2 (Delta) variant of COVID-19. World J Pediatr. 2022;18(5):343-9.

For peer review only - http://bmjopen.bmj.com/site/about/guidelines.xhtml

25. Nygaard U, Holm M, Hartling UB, Glenthoj J, Schmidt LS, Nordly SB, et al. Incidence and clinical phenotype of multisystem inflammatory syndrome in children after infection with the SARS-CoV-2 delta variant by vaccination status: a Danish nationwide prospective cohort study. Lancet Child Adolesc Health. 2022;6(7):459-65.

26. Murillo-Zamora E, Trujillo X, Huerta M, Rios-Silva M, Baltazar-Rodriguez LM, Guzman-Esquivel J, et al. Decreased risk of COVID-19 pneumonia in children and adolescents during the Delta variant emergence. Public Health. 2022;204:9-11. doi: 10.1016/j.puhe.2021.12.017.

27. Ryu BH, Hong SI, Lim SJ, Cho Y, Hong KW, Bae IG, et al. Features of COVID-19 Among Children and Adolescents Without Risk Factors Before and After the Delta Variant Outbreak in South Korea. Pediatr Infect Dis J. 2022;41(1):e34-e5. doi: 10.1097/INF.0000000000003394.

28. Fisman DN, Tuite AR. Age-Specific Changes in Virulence Associated with SARS-CoV-2 Variants of Concern. Clin Infect Dis. 2022. doi: 10.1093/cid/ciac174.

29. Shoji K, Akiyama T, Tsuzuki S, Matsunaga N, Asai Y, Suzuki S, et al. Comparison of the clinical characteristics and outcomes of COVID-19 in children before and after the emergence of Delta variant of concern in Japan. J Infect Chemother. 2022;28(4):591-4. doi: 10.1016/j.jiac.2022.01.009.

30. Martin B, DeWitt PE, Russell S, Anand A, Bradwell KR, Bremer C, et al. Characteristics, Outcomes, and Severity Risk Factors Associated With SARS-CoV-2 Infection Among Children in the US National COVID Cohort Collaborative. JAMA Netw Open. 2022;5(2):e2143151. doi: 10.1001/jamanetworkopen.2021.43151.

31. Butt AA, Dargham SR, Chemaitelly H, Al Khal A, Tang P, Hasan MR, et al. Severity of Illness in Persons Infected With the SARS-CoV-2 Delta Variant vs Beta Variant in Qatar. JAMA Intern Med. 2022;182(2):197-205. doi: 10.1001/jamainternmed.2021.7949.

32. Molteni E, Sudre CH, Canas LS, Bhopal SS, Hughes RC, Chen L, et al. Illness characteristics of COVID-19 in children infected with the SARS-CoV-2 Delta variant. MedRxiv. 2021. doi: 10.1101/2021.10.06.21264467.

33. Kildegaard H, Lund LC, Hojlund M, Stensballe LG, Pottegard A. Risk of adverse events after covid-19 in Danish children and adolescents and effectiveness of BNT162b2 in adolescents: cohort study. BMJ. 2022;377:e068898.

34. Yilmaz Celebi M, Kiymet E, Boncuoglu E, Sahinkaya S, Cem E, Duzgol M, et al. Evaluation of childhood hospitalization rates and degree of severity of SARS-CoV-2 variants, including B.1.1.7 (Alpha), B.1.315/P.1 (Beta/Gamma), and B.1.617.2 (Delta). J Med Virol. 2022;94(5):2050-4. doi: 10.1002/jmv.27587.

35. Edward PR, Lorenzo-Redondo R, Reyna ME, Simons LM, Hultquist JF, Patel AB, et al. Severity of Illness Caused by Severe Acute Respiratory Syndrome Coronavirus 2 Variants of Concern in Children: A Single-Center Retrospective Cohort Study. medRxiv. 2021. doi: 10.1101/2021.10.23.21265402.

36. Cloete J, Kruger A, Masha M, du Plessis NM, Mawela D, Tshukudu M, et al. Paediatric hospitalisations due to COVID-19 during the first SARS-CoV-2 omicron (B.1.1.529) variant wave in South Africa: a multicentre observational study. Lancet Child Adolesc Health. 2022. doi: 10.1016/S2352-4642(22)00027-X.

For peer review only - http://bmjopen.bmj.com/site/about/guidelines.xhtml

37. Brewster RC, Parsons C, Laird-Gion J, Hilker S, Irwin M, Sommerschield A, et al. COVID-19-Associated Croup in Children. Pediatrics. 2022;149(6).

38. Ludvigsson JF. Convulsions in children with COVID-19 during the Omicron wave. Acta Paediatr. 2022. doi: 10.1111/apa.16276.

39. Butt AA, Dargham SR, Loka S, Shaik RM, Chemaitelly H, Tang P, et al. COVID-19 Disease Severity in Children Infected with the Omicron Variant. Clin Infect Dis. 2022.

40. Fowlkes AL, Yoon SK, Lutrick K, Gwynn L, Burns J, Grant L, et al. Effectiveness of 2-Dose BNT162b2 (Pfizer BioNTech) mRNA Vaccine in Preventing SARS-CoV-2 Infection Among Children Aged 5-11 Years and Adolescents Aged 12-15 Years - PROTECT Cohort, July 2021-February 2022. MMWR Morb Mortal Wkly Rep. 2022;71(11):422-8.

41. Wang L, Berger NA, Kaelber DC, Davis PB, Volkow ND, Xu R. COVID infection severity in children under 5 years old before and after Omicron emergence in the US. medRxiv. 2022. doi: 10.1101/2022.01.12.22269179.

42. Wang L, Berger NA, Kaelber DC, Davis PB, Volkow ND, Xu R. COVID infection rates, clinical outcomes, and racial/ethnic and gender disparities before and after Omicron emerged in the US. medRxiv. 2022. doi: 10.1101/2022.02.21.22271300.

43. Marks KJ, Whitaker M, Anglin O, Milucky J, Patel K, Pham H, et al. Hospitalizations of Children and Adolescents with Laboratory-Confirmed COVID-19 - COVID-NET, 14 States, July 2021-January 2022. MMWR Morb Mortal Wkly Rep. 2022;71(7):271-8. doi: 10.15585/mmwr.mm7107e4.

44. Iuliano AD, Brunkard JM, Boehmer TK, Peterson E, Adjei S, Binder AM, et al. Trends in Disease Severity and Health Care Utilization During the Early Omicron Variant Period Compared with Previous SARS-CoV-2 High Transmission Periods - United States, December 2020-January 2022. MMWR Morb Mortal Wkly Rep. 2022;71(4):146-52. doi: 10.15585/mmwr.mm7104e4.

45. Shi DS, Whitaker M, Marks KJ, Anglin O, Milucky J, Patel K, et al. Hospitalizations of Children Aged 5-11 Years with Laboratory-Confirmed COVID-19 - COVID-NET, 14 States, March 2020-February 2022. MMWR Morb Mortal Wkly Rep. 2022;71(16):574-81. doi: 10.15585/mmwr.mm7116e1.

46. Marks KJ, Whitaker M, Agathis NT, Anglin O, Milucky J, Patel K, et al. Hospitalization of Infants and Children Aged 0-4 Years with Laboratory-Confirmed COVID-19 - COVID-NET, 14 States, March 2020-February 2022. MMWR Morb Mortal Wkly Rep. 2022;71(11):429-36.

47. Oliveira EA, Simoes ESAC, Oliveira MCL, Colosimo EA, Mak RH, Vasconcelos MA, et al. Comparison of the First and Second Waves of the Coronavirus Disease 2019 (COVID-19) Pandemic in Children and Adolescents in a Middle-Income Country: Clinical Impact Associated with Severe Acute Respiratory Syndrome Coronavirus 2 (SARS-CoV-2) Gamma Lineage. J Pediatr. 2022. doi: 10.1016/j.jpeds.2022.01.001.

48. Edward PR, Lorenzo-Redondo R, Reyna ME, Simons LM, Hultquist JF, Patel AB, et al. Severity of Illness Caused by Severe Acute Respiratory Syndrome Coronavirus 2 Variants of Concern in Children: A Single-Center Retrospective Cohort Study. medRxiv. 2021. doi: 10.1101/2021.10.23.21265402.

For peer review only - http://bmjopen.bmj.com/site/about/guidelines.xhtml

49. Baker JM, Buchfellner M, Britt W, Sanchez V, Potter JL, Ingram LA, et al. Acute Hepatitis and Adenovirus Infection Among Children - Alabama, October 2021-February 2022. MMWR Morb Mortal Wkly Rep. 2022;71(18):638-40.

50. Schenk B, Hoehl S, Rudych O, Menger D, Farmand S, Wrobel F, et al. Longitudinal testing for SARS-CoV-2 RNA in day care centers in Hesse, Germany, during increased local incidence and with VOC Alpha as dominant variant: Results of the SAFE KiDS 2 and SAFE KiDS 3 study. MedRxiv. 2021. doi: 10.1101/2021.06.29.21259633.

51. Neuberger F, Grgic M, Diefenbacher S, Spensberger F, Lehfeld A-S, Buchholz U, et al. COVID-19 infections in day care centres in Germany: Social and organizational determinants of infections in children and staff in the second and third wave of the pandemic. MedRxiv. 2021. doi: 10.1101/2021.06.07.21257958.

52. Loenenbach A, Markus I, Lehfeld AS, An der Heiden M, Haas W, Kiegele M, et al. SARS-CoV-2 variant B.1.1.7 susceptibility and infectiousness of children and adults deduced from investigations of childcare centre outbreaks, Germany, 2021. Euro Surveill. 2021;26(21). doi: 10.2807/1560-7917.ES.2021.26.21.2100433.

53. Lorthe E, Bellon M, Michielin G, Berthelot J, Zaballa M-E, Pennacchio F, et al. Epidemiological, virological and serological investigation into a SARS-CoV-2 outbreak (Alpha variant) in a primary school: a prospective longitudinal study. medRxiv. 2021. doi: 10.1101/2021.10.26.21265509.

54. Abu-Raddad LJ, Chemaitelly H, Ayoub HH, Coyle P, Malek JA, Ahmed AA, et al. Introduction and expansion of the SARS-CoV-2 B.1.1.7 variant and reinfections in Qatar: A nationally representative cohort study. PLoS Med. 2021;18(12):e1003879. doi: 10.1371/journal.pmed.1003879.

55. Clifford S, Waight P, Hackman J, Hué S, Gower CM, Kirsebom FCM, et al. Effectiveness of BNT162b2 and ChAdOx1 against SARS-CoV-2 household transmission - a prospective cohort study in England. MedRxiv. 2021. doi: 10.1101/2021.11.24.21266401.

56. Ng OT, Koh V, Chiew CJ, Marimuthu K, Thevasagayam NM, Mak TM, et al. Impact of SARS-CoV-2 Vaccination and Paediatric Age on Delta Variant Household Transmission. Clin Infect Dis. 2022.

57. Dougherty K, Mannell M, Naqvi O, Matson D, Stone J. SARS-CoV-2 B.1.617.2 (Delta) Variant COVID-19 Outbreak Associated with a Gymnastics Facility - Oklahoma, April-May 2021. MMWR Morb Mortal Wkly Rep. 2021;70(28):1004-7. doi: 10.15585/mmwr.mm7028e2.

58. Singanayagam A, Hakki S, Dunning J, Madon KJ, Crone MA, Koycheva A, et al. Community transmission and viral load kinetics of the SARS-CoV-2 delta (B.1.617.2) variant in vaccinated and unvaccinated individuals in the UK: a prospective, longitudinal, cohort study. Lancet Infect Dis. 2022;22(2):183-95. doi: 10.1016/S1473-3099(21)00648-4.

59. Lorthe E, Bellon M, Berthelot J, Michielin G, L'Huillier AG, Posfay-Barbe KM, et al. A SARS-CoV-2 omicron (B.1.1.529) variant outbreak in a primary school in Geneva, Switzerland. Lancet Infect Dis. 2022;22(6):767-8.

For peer review only

For peer review only - http://bmjopen.bmj.com/site/about/guidelines.xhtml

60. Lindstrom JC, Engebretsen S, Kristoffersen AB, Ro GOI, Palomares AD, Engo-Monsen K, et al. Increased transmissibility of the alpha SARS-CoV-2 variant: evidence from contact tracing data in Oslo, January to February 2021. Infectious diseases (London, England). 2022;54(1):72-7. doi: 10.1080/23744235.2021.1977382.

61. Lyngse FP, Molbak K, Skov RL, Christiansen LE, Mortensen LH, Albertsen M, et al. Increased transmissibility of SARS-CoV-2 lineage B.1.1.7 by age and viral load. Nat Commun. 2021;12(1):7251. doi: 10.1038/s41467-021-27202-x.

62. Chudasama DY, Flannagan J, Collin SM, Charlett A, Twohig KA, Lamagni T, et al. Household clustering of SARS-CoV-2 variant of concern B.1.1.7 (VOC-202012-01) in England. J Infect. 2021;83(1):e26-e8. doi: 10.1016/j.jinf.2021.04.029.

63. Julin CH, Robertson AH, Hungnes O, Tunheim G, Bekkevold T, Laake I, et al. Household Transmission of SARS-CoV-2: A Prospective Longitudinal Study Showing Higher Viral Load and Increased Transmissibility of the Alpha Variant Compared to Previous Strains. Microorganisms. 2021;9(11). doi: 10.3390/microorganisms9112371.

64. Loss J, Wurm J, Varnaccia G, Schienkiewitz A, Iwanowski H, Loer A-KM, et al. Transmission of SARS-CoV-2 among children and staff in German daycare centers: results from the COALA study. MedRxiv. 2021. doi: 10.1101/2021.12.21.21268157.

65. Buchan SA, Tibebu S, Daneman N, Whelan M, Vanniyasingam T, Murti M, et al. Increased Household Secondary Attacks Rates With Variant of Concern Severe Acute Respiratory Syndrome Coronavirus 2 Index Cases. Clin Infect Dis. 2022;74(4):703-6. doi: 10.1093/cid/ciab496.

66. Ratmann O, Bhatt S, Flaxman S. Implications of a highly transmissible variant of SARS-CoV-2 for children. Arch Dis Child. 2021;106(9):e37. doi: 10.1136/archdischild-2021-321903.

67. Allen H, Vusirikala A, Flannagan J, Twohig KA, Zaidi A, Chudasama D, et al. Household transmission of COVID-19 cases associated with SARS-CoV-2 delta variant (B.1.617.2): national case-control study. Lancet Reg Health Eur. 2022;12:100252. doi: 10.1016/j.lanepe.2021.100252.

68. Trobajo-Sanmartin C, Martinez-Baz I, Miqueleiz A, Fernandez-Huerta M, Burgui C, Casado I, et al. Differences in Transmission between SARS-CoV-2 Alpha (B.1.1.7) and Delta (B.1.617.2) Variants. Microbiol Spectr. 2022;10(2):e0000822.

69. Pedersen ESL, Collaud ENR, Mozun R, Dexter K, Kruljac C, Silberschmidt H, et al. Facemask Usage Among People With Primary Ciliary Dyskinesia During the COVID-19 Pandemic: A Participatory Project. Int J Public Health. 2021;66:1604277.

70. Lin DY, Gu Y, Wheeler B, Young H, Holloway S, Sunny SK, et al. Effectiveness of Covid-19 Vaccines over a 9-Month Period in North Carolina. N Engl J Med. 2022;386(10):933-41.

71. Eyre DW, Taylor D, Purver M, Chapman D, Fowler T, Pouwels KB, et al. Effect of Covid-19 Vaccination on Transmission of Alpha and Delta Variants. N Engl J Med. 2022;386(8):744-56.

72. Price AM, Olson SM, Newhams MM, Halasa NB, Boom JA, Sahni LC, et al. BNT162b2 Protection against the Omicron Variant in Children and Adolescents. N Engl J Med. 2022;386(20):1899-909.

For peer review only - http://bmjopen.bmj.com/site/about/guidelines.xhtml

73. Tan SHX, Cook AR, Heng D, Ong B, Lye DC, Tan KB. Effectiveness of BNT162b2 Vaccine against Omicron in Children 5 to 11 Years of Age. N Engl J Med. 2022;387(6):525-32.

74. Sacco C, Del Manso M, Mateo-Urdiales A, Rota MC, Petrone D, Riccardo F, et al. Effectiveness of BNT162b2 vaccine against SARS-CoV-2 infection and severe COVID-19 in children aged 5-11 years in Italy: a retrospective analysis of January-April, 2022. Lancet. 2022;400(10346):97-103.

75. Hunter DJ, Abdool Karim SS, Baden LR, Farrar JJ, Hamel MB, Longo DL, et al. Addressing Vaccine Inequity - Covid-19 Vaccines as a Global Public Good. N Engl J Med. 2022;386(12):1176-9.

76. Food and Drug Administration. Coronavirus (COVID-19) Update: FDA Authorizes Moderna and Pfizer-BioNTech COVID-19 Vaccines for Children Down to 6 Months of Age 2022 [Available from: https://www.fda.gov/news-events/press-announcements/coronavirus-covid-19-update-fda-authorizes-moderna-and-pfizer-biontech-covid-19-vaccines-children.]

77. GOV.UK. Pfizer/BioNTech COVID-19 vaccine authorised for use in infants and children aged 6 months to 4 years 2022 [Available from: https://www.gov.uk/government/news/pfizerbiontech-covid-19-vaccine-authorised-for-use-in-infants-and-children-aged-6-months-to-4-years.]

For peer review only

For peer review only - http://bmjopen.bmj.com/site/about/guidelines.xhtml

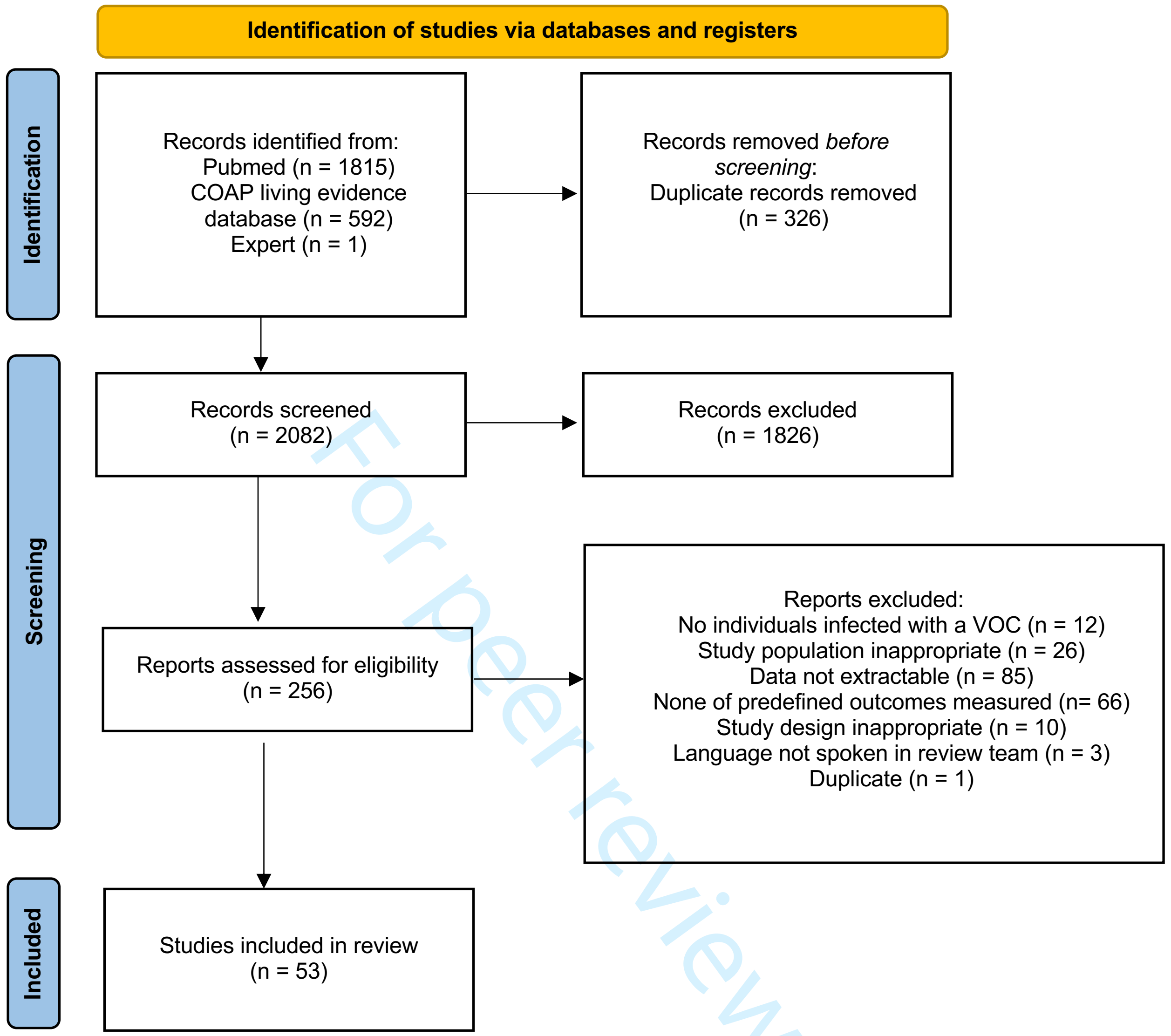

For peer review only

Figure 1: Flow chart
Of the 2407 studies found via database searches 53 were found to be eligible for this systematic review (incl. 1 study recommended to us by experts).

From: Page MJ, McKenzie JE, Bossuyt PM, Boutron I, Hoffmann TC, Mulrow CD, et al. The PRISMA 2020 statement: an updated guideline for reporting systematic reviews. BMJ 2021;372:n71. doi: 10.1136/bmj.n71

For more information, visit: http://www.prisma-statement.org/

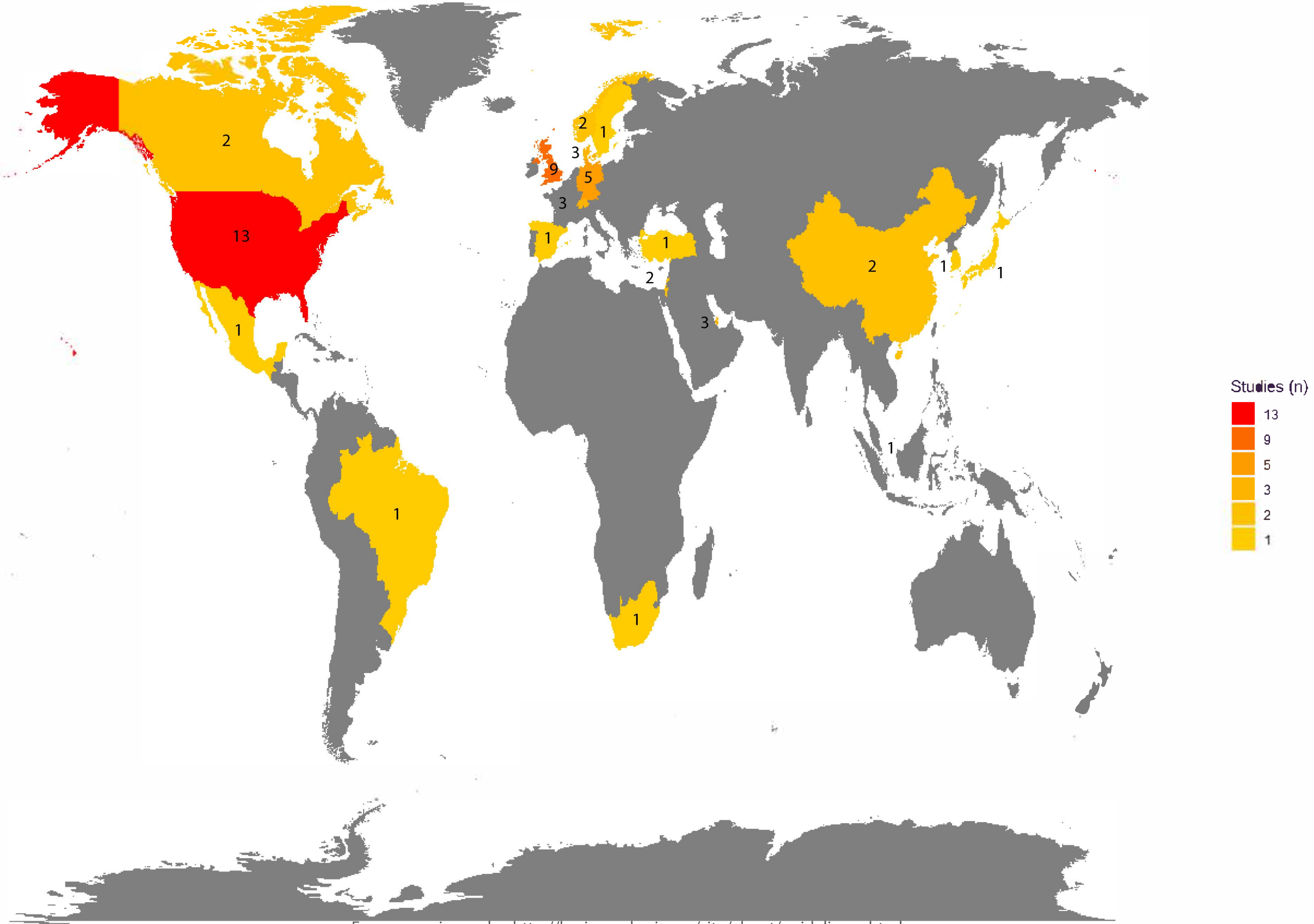

For peer review only - http://bmjopen.bmj.com/site/about/guidelines.xhtml

Figure 2. Map of the number of studies conducted in each country

Most studies came from high-income countries (n = 47, 89%). 6 studies (11%) were from upper-middle income countries.

URL to search strategy

a) COAP database search terms: (child* AND variant*) in title or abstract
b) PubMed search child*"[Title/Abstract] OR "neonat*"[Title/Abstract] OR "adolesc*"[Title/Abstract] OR "infan*"[Title/Abstract] OR "teen*"[Title/Abstract] OR "pediatr*"[Title/Abstract] OR "paediatr*"[Title/Abstract] OR ("educational status"[MeSH Terms] OR ("educational"[Title/Abstract] AND "status"[Title/Abstract]) OR "educational status"[Title/Abstract] OR "schooling"[Title/Abstract] OR "education"[MeSH Terms] OR "education"[Title/Abstract] OR "school s"[Title/Abstract] OR "schooled"[Title/Abstract] OR "schools"[MeSH Terms] OR "schools"[Title/Abstract] OR "school"[Title/Abstract]) OR ("nurseries, infant"[MeSH Terms] OR ("nurseries"[Title/Abstract] AND "infant"[Title/Abstract]) OR "infant nurseries"[Title/Abstract] OR "nurseries"[Title/Abstract] OR "nursery"[Title/Abstract]) OR ("infant, newborn"[MeSH Terms] OR ("infant"[Title/Abstract] AND "newborn"[Title/Abstract]) OR "newborn infant"[Title/Abstract] OR "newborn"[Title/Abstract] OR "newborns"[Title/Abstract] OR "newborn s"[Title/Abstract]) OR "toddler*"[Title/Abstract] OR ("minority groups"[MeSH Terms] OR ("minority"[Title/Abstract] AND "groups"[Title/Abstract]) OR "minority groups"[Title/Abstract] OR "minorities"[Title/Abstract] OR "minority"[Title/Abstract] OR "minority s"[Title/Abstract] OR "minors"[MeSH Terms] OR "minors"[Title/Abstract] OR "minor"[Title/Abstract])
AND
"COVID-19"[tiab] OR 2019-novel-cov*[tiab] OR coronavirus[tiab] OR coronavirus-disease-19*[tiab] OR corona-virus-disease-19*[tiab] OR covid-19*[tiab] OR covid19*[tiab] OR new-coronavirus[tiab] OR new-corona-virus[tiab] OR novel-coronavirus[tiab] OR novel-corona-virus[tiab] OR sars-2*[tiab] OR sars2*[tiab] OR sars-cov-19*[tiab] OR sarscov19*[tiab] OR sars-cov-2*[tiab] OR sars-cov2*[tiab] OR sarscov2*[tiab] OR sarscov-2*[tiab]
AND
"VOC"[All Fields] OR "variant of concern"[All Fields] OR "alpha variant"[All Fields] OR "beta variant"[All Fields] OR "gamma variant"[All Fields] OR "delta variant"[All Fields] OR "omicron variant"[All Fields] OR "variant of interest"[All Fields]

For peer review only

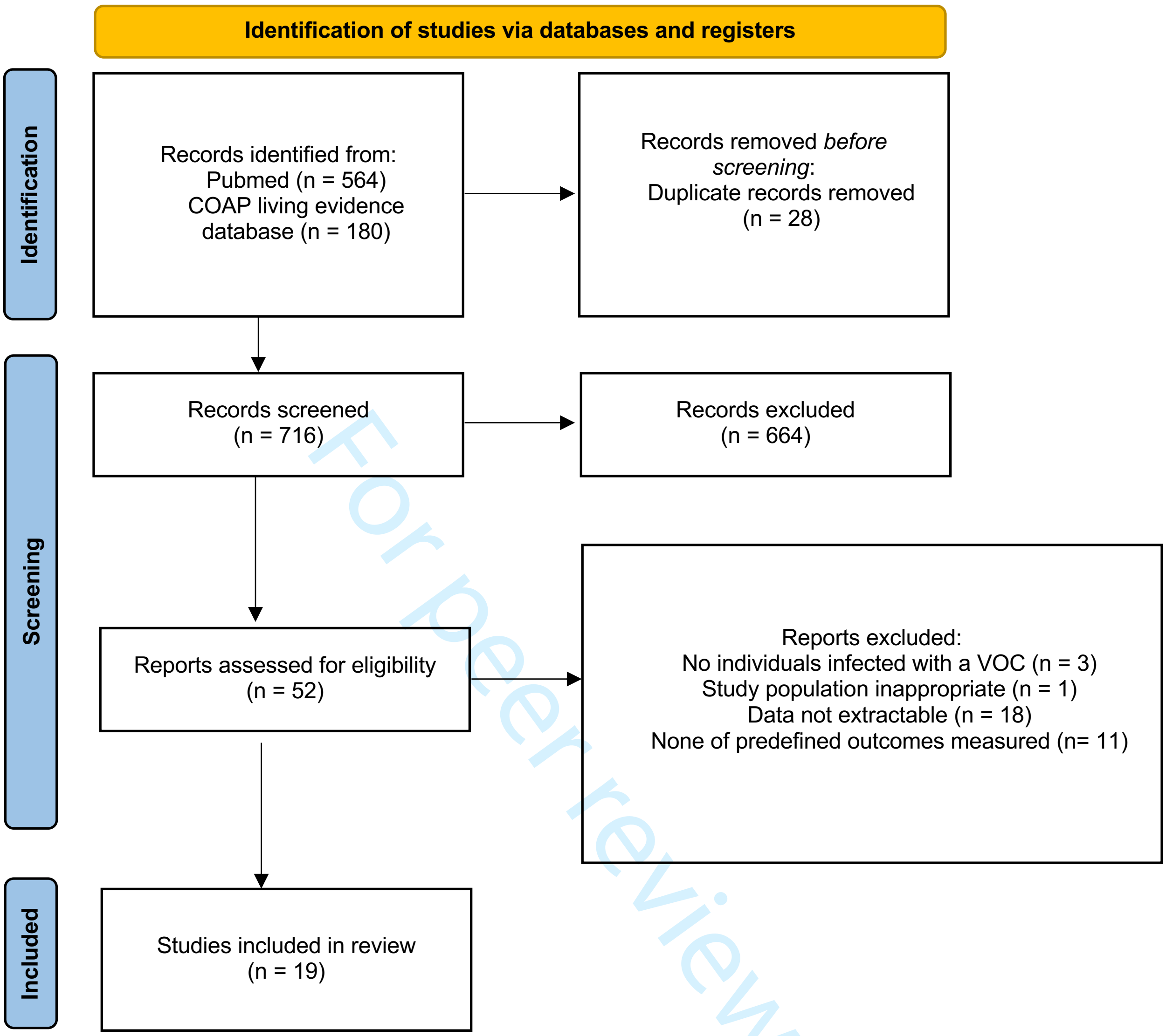

Supplementary figure 1: Flow chart – first search (until 15.10.2021)
Of the 744 studies found via database searches 19 were found to be eligible for this systematic review.

From: Page MJ, McKenzie JE, Bossuyt PM, Boutron I, Hoffmann TC, Mulrow CD, et al. The PRISMA 2020 statement: an updated guideline for reporting systematic reviews. BMJ 2021;372:n71. doi: 10.1136/bmj.n71

For peer review only

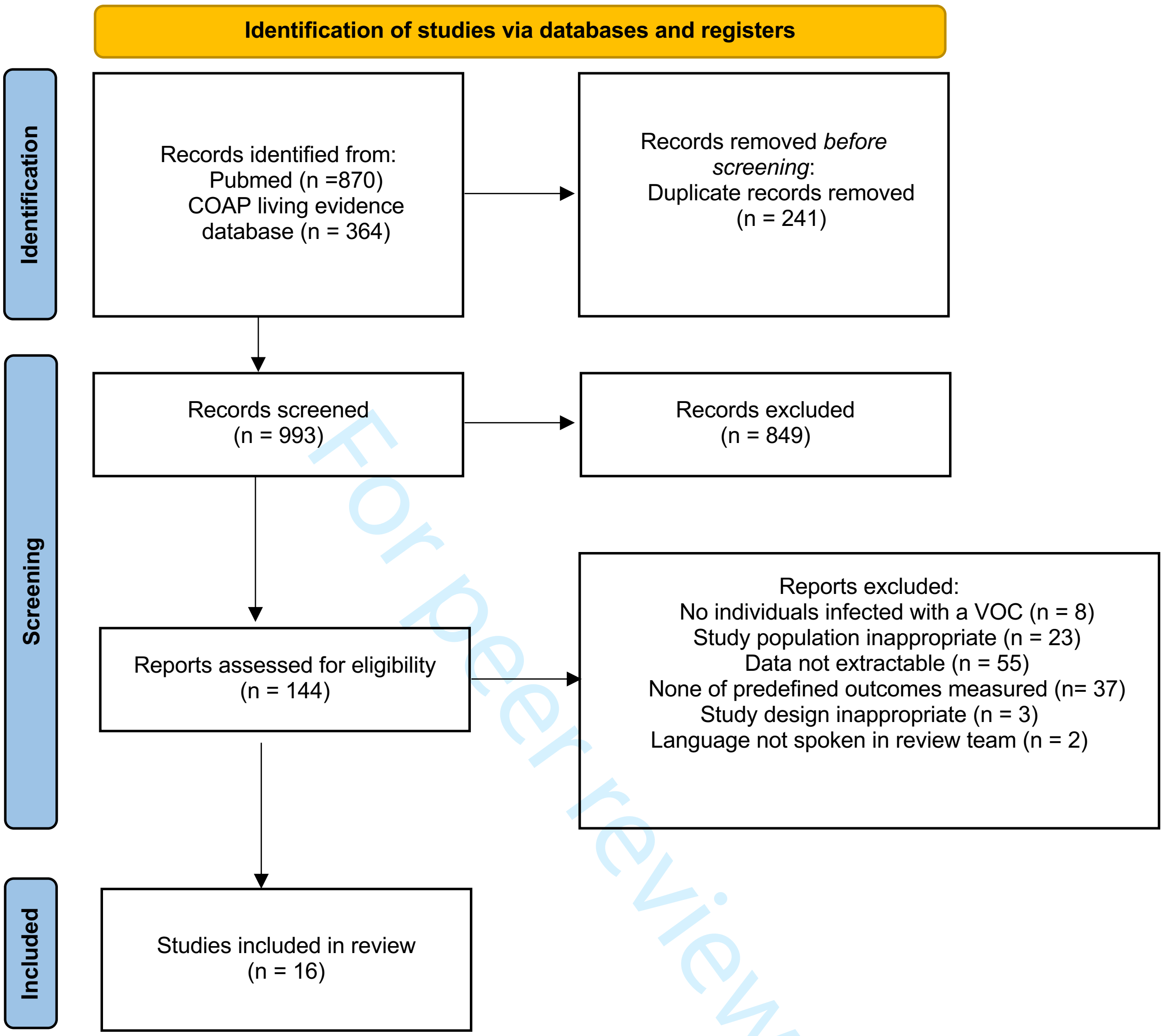

Supplementary figure 2: Flow chart – second search (15.10.2021-31.01.2022)
Of the 1234 studies found via database searches 16 were found to be eligible for this systematic review.

From: Page MJ, McKenzie JE, Bossuyt PM, Boutron I, Hoffmann TC, Mulrow CD, et al. The PRISMA 2020 statement: an updated guideline for reporting systematic reviews. BMJ 2021;372:n71. doi: 10.1136/bmj.n71

For peer review only

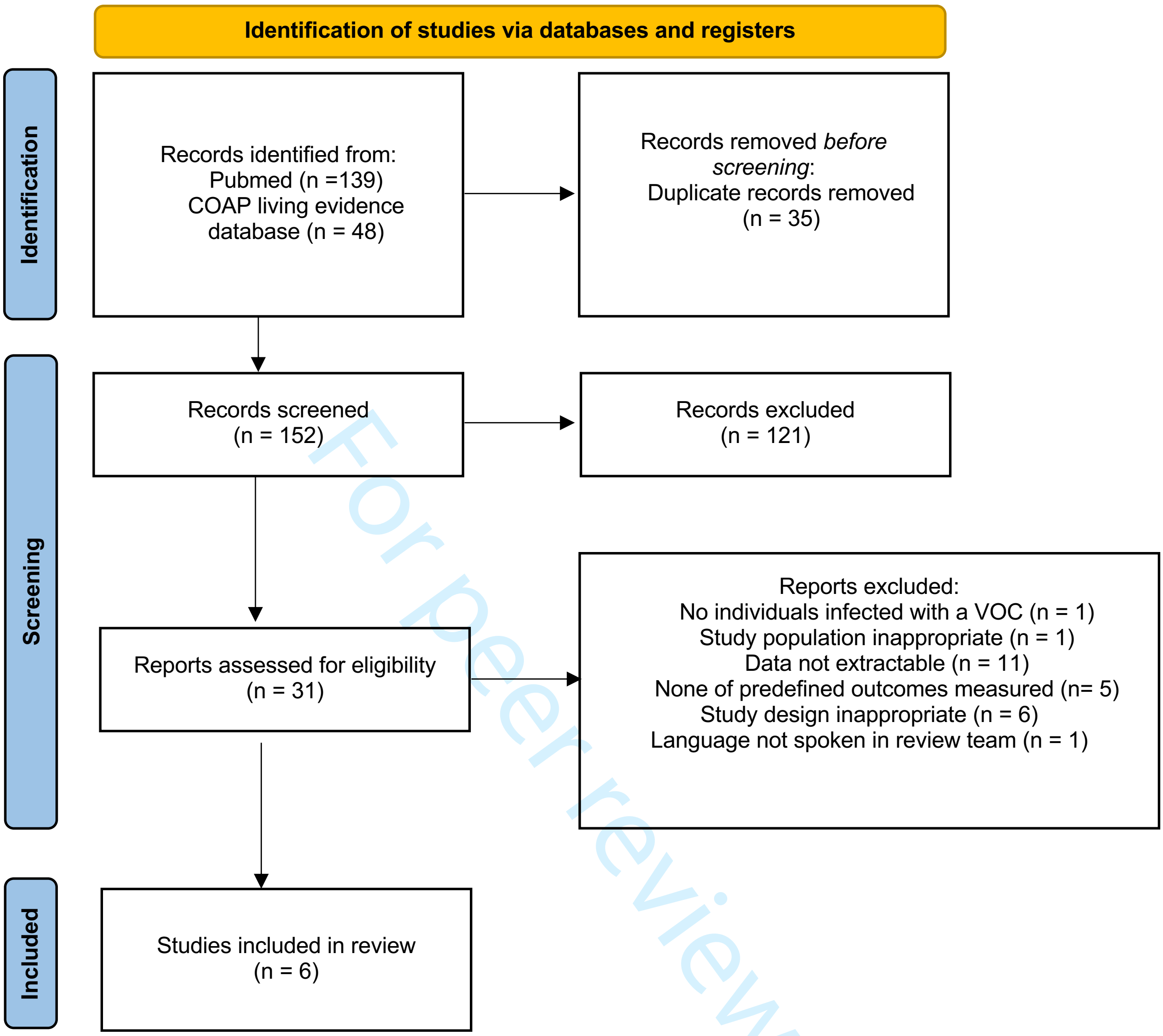

Supplementary figure 3: Flow chart – third search (31.01.2022-07.03.2022)
Of the 187 studies found via database searches 6 were found to be eligible for this systematic review.

From: Page MJ, McKenzie JE, Bossuyt PM, Boutron I, Hoffmann TC, Mulrow CD, et al. The PRISMA 2020 statement: an updated guideline for reporting systematic reviews. BMJ 2021;372:n71. doi: 10.1136/bmj.n71

For peer review only

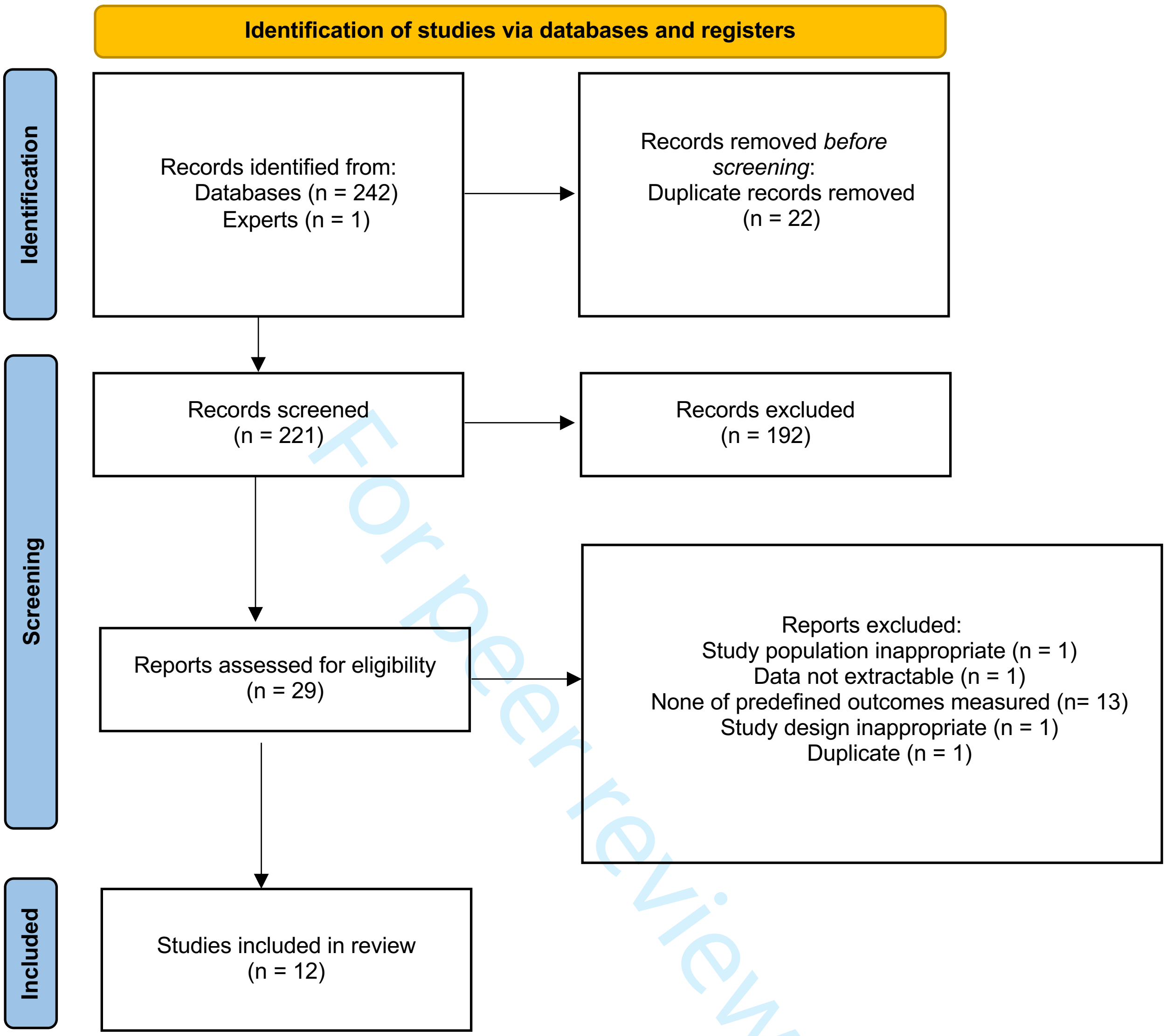

Supplementary figure 4: Flow chart – fourth search (07.03.2022-09.05.2022)
Of the 243 studies found via the database search (incl. 1 study recommended by experts) 12 studies were found to be eligible.

From: Page MJ, McKenzie JE, Bossuyt PM, Boutron I, Hoffmann TC, Mulrow CD, et al. The PRISMA 2020 statement: an updated guideline for reporting systematic reviews. BMJ 2021;372:n71. doi: 10.1136/bmj.n71

For more information, visit: http://www.prisma-statement.org/

For peer review only

PRISMA 2020 Checklist

Section and Topic	Item #	Checklist item	Location where item is reported
**TITLE**			
Title	1	Identify the report as a systematic review.	Page 1
**ABSTRACT**			
Abstract	2	See the PRISMA 2020 for Abstracts checklist.	Page 2
**INTRODUCTION**			
Rationale	3	Describe the rationale for the review in the context of existing knowledge.	Page 4
Objectives	4	Provide an explicit statement of the objective(s) or question(s) the review addresses.	Page 4
**METHODS**			
Eligibility criteria	5	Specify the inclusion and exclusion criteria for the review and how studies were grouped for the syntheses.	Page 5
Information sources	6	Specify all databases, registers, websites, organisations, reference lists and other sources searched or consulted to identify studies. Specify the date when each source was last searched or consulted.	Page 5
Search strategy	7	Present the full search strategies for all databases, registers and websites, including any filters and limits used.	Supplementary note 1
Selection process	8	Specify the methods used to decide whether a study met the inclusion criteria of the review, including how many reviewers screened each record and each report retrieved, whether they worked independently, and if applicable, details of automation tools used in the process.	Page 5
Data collection process	9	Specify the methods used to collect data from reports, including how many reviewers collected data from each report, whether they worked independently, any processes for obtaining or confirming data from study investigators, and if applicable, details of automation tools used in the process.	Page 5
Data items	10a	List and define all outcomes for which data were sought. Specify whether all results that were compatible with each outcome domain in each study were sought (e.g. for all measures, time points, analyses), and if not, the methods used to decide which results to collect.	Page 5-6
	10b	List and define all other variables for which data were sought (e.g. participant and intervention characteristics, funding sources). Describe any assumptions made about any missing or unclear information.	Page 5-6
Study risk of bias assessment	11	Specify the methods used to assess risk of bias in the included studies, including details of the tool(s) used, how many reviewers assessed each study and whether they worked independently, and if applicable, details of automation tools used in the process.	Page 6
Effect measures	12	Specify for each outcome the effect measure(s) (e.g. risk ratio, mean difference) used in the synthesis or presentation of results.	n/a
Synthesis methods	13a	Describe the processes used to decide which studies were eligible for each synthesis (e.g. tabulating the study intervention characteristics and comparing against the planned groups for each synthesis (item #5)).	Page 6
	13b	Describe any methods required to prepare the data for presentation or synthesis, such as handling of missing summary statistics, or data conversions.	Page 6
	13c	Describe any methods used to tabulate or visually display results of individual studies and syntheses.	Page 6
	13d	Describe any methods used to synthesize results and provide a rationale for the choice(s). If meta-analysis was performed, describe the model(s), method(s) to identify the presence and extent of statistical heterogeneity, and software package(s) used.	Page 6
	13e	Describe any methods used to explore possible causes of heterogeneity among study results (e.g. subgroup analysis, meta-regression).	n/a
	13f	Describe any sensitivity analyses conducted to assess robustness of the synthesized results.	n/a
Reporting bias assessment	14	Describe any methods used to assess risk of bias due to missing results in a synthesis (arising from reporting biases).	Page 6
Certainty	15	Describe any methods used to assess certainty (or confidence) in the body of evidence for an outcome.	Page 6

For peer review only - http://bmjopen.bmj.com/site/about/guidelines.xhtml

PRISMA 2020 Checklist

Section and Topic	Item #	Checklist item	Location where item is reported
assessment			
**RESULTS**			
Study selection	16a	Describe the results of the search and selection process, from the number of records identified in the search to the number of studies included in the review, ideally using a flow diagram.	Page 7, figure 1, supplementary figures 1-4
	16b	Cite studies that might appear to meet the inclusion criteria, but which were excluded, and explain why they were excluded.	Figure 1, supplementary figures 1-4
Study characteristics	17	Cite each included study and present its characteristics.	Supplementary tables 7-12
Risk of bias in studies	18	Present assessments of risk of bias for each included study.	Supplementary tables 2-6
Results of individual studies	19	For all outcomes, present, for each study: (a) summary statistics for each group (where appropriate) and (b) an effect estimate and its precision (e.g. confidence/credible interval), ideally using structured tables or plots.	Supplementary tables 7-12
Results of syntheses	20a	For each synthesis, briefly summarise the characteristics and risk of bias among contributing studies.	Page 13
	20b	Present results of all statistical syntheses conducted. If meta-analysis was done, present for each the summary estimate and its precision (e.g. confidence/credible interval) and measures of statistical heterogeneity. If comparing groups, describe the direction of the effect.	n/a
	20c	Present results of all investigations of possible causes of heterogeneity among study results.	Page 7-12
	20d	Present results of all sensitivity analyses conducted to assess the robustness of the synthesized results.	n/a
Reporting biases	21	Present assessments of risk of bias due to missing results (arising from reporting biases) for each synthesis assessed.	n/a
Certainty of evidence	22	Present assessments of certainty (or confidence) in the body of evidence for each outcome assessed.	Page 7
**DISCUSSION**			
Discussion	23a	Provide a general interpretation of the results in the context of other evidence.	Page 12-15
	23b	Discuss any limitations of the evidence included in the review.	Page 12-13
	23c	Discuss any limitations of the review processes used.	Page 12-13
	23d	Discuss implications of the results for practice, policy, and future research.	Page 12-15
**OTHER INFORMATION**			
Registration and protocol	24a	Provide registration information for the review, including register name and registration number, or state that the review was not registered.	Page 2,5
	24b	Indicate where the review protocol can be accessed, or state that a protocol was not prepared.	Page 5
	24c	Describe and explain any amendments to information provided at registration or in the protocol.	n/a
Support	25	Describe sources of financial or non-financial support for the review, and the role of the funders or sponsors in the review.	Page 16
Competing interests	26	Declare any competing interests of review authors.	Page 16
Availability of data, code and	27	Report which of the following are publicly available and where they can be found: template data collection forms; data extracted from included	extracted and used data:

For peer review only

PRISMA 2020 Checklist

Section and Topic	Item #	Checklist item	Location where item is reported
other materials		studies; data used for all analyses; analytic code; any other materials used in the review.	supplementary tables 2-12

From: Page MJ, McKenzie JE, Bossuyt PM, Boutron I, Hoffmann TC, Mulrow CD, et al. The PRISMA 2020 statement: an updated guideline for reporting systematic reviews. BMJ 2021;372:n71. doi: 10.1136/bmj.n71

For more information, visit: http://www.prisma-statement.org/

For peer review only

Supplementary table 2: Risk of bias assessment - Child/adolescent symptoms/severity of disease when infected with a VOC

Cohort studies[1]	Exposed and non-exposed cohorts from the same population	Confidence in exposure assessment	Confidence that outcome of interest was not present at start of study	Matching of exposed and unexposed for all variables associated with outcome of interest or adjustment of prognostic variables by statistical analysis	Confidence in assessment of presence/absence of prognostic factors	Confidence in outcome assessment	Adequate follow-up of cohorts	Similar co-interventions between groups
Ulyte et al. [15]	definitely yes	probably no	definitely no	probably no	probably yes	probably no	definitely no	definitely yes
Nakel et al. [18]	probably yes	definitely yes	probably yes	definitely no	definitely no	probably no	definitely no	probably yes
Somekh et al. [19]	probably yes	definitely yes	probably yes	probably no	probably no	definitely yes	probably yes	probably no
Swann et al. [20]	definitely yes	definitely yes	probably no	probably yes	definitely yes	definitely yes	probably yes	probably yes
Edward et al. [35]	probably yes	definitely yes	definitely yes	probably yes	probably no	definitely yes	probably yes	probably no
Celebi et al. [34]	probably yes	probably yes	definitely yes	definitely no	definitely no	probably yes	probably no	definitely no
Stewart et al. [22]	probably yes	definitely yes	probably yes	probably no	probably no	definitely yes	definitely yes	probably no
Brookman et al. [21]	probably yes	definitely yes	definitely yes	definitely no	definitely no	probably yes	probably no	probably yes
Li et al. [23]	definitely yes	definitely yes	probably yes	definitely yes	probably no	probably yes	definitely yes	definitely yes
Shoji et al. [29]	definitely yes	definitely yes	definitely yes	probably no	probably yes	definitely yes	probably yes	probably no
Fisman et al. [28]	probably yes	definitely yes	definitely yes	probably yes	probably yes	definitely yes	probably yes	probably yes
Ryu et al. [27]	probably yes	definitely yes	probably yes	definitely no	definitely no	probably yes	probably no	probably no
Martin et al. [30]	probably yes	probably yes	definitely yes	probably no	probably no	definitely yes	probably no	definitely no
Molteni et al. [32]	definitely yes	probably yes	probably yes	probably yes	probably no	probably yes	probably yes	probably no
Murillo-Zamora et al. [26]	probably yes	definitely yes	definitely yes	definitely no	probably no	definitely yes	probably no	probably no
Butt et al. [31]	probably yes	probably yes	probably yes	probably yes	probably no	definitely yes	probably no	probably no
Oliveira et al. [47]	probably yes	definitely yes	definitely yes	definitely no	definitely no	definitely yes	probably no	probably no
Cloete et al. [36]	probably yes	probably yes	probably yes	probably yes	probably yes	probably yes	definitely yes	definitely yes
Wang et al. [41]	probably yes	probably yes	probably yes	probably yes	probably yes	definitely yes	probably no	probably no
Wang et al. [42]	probably yes	probably yes	probably yes	probably no	probably no	definitely yes	probably no	probably no

[1] CLARITY Group at McMaster University. Tool to Assess Risk of Bias in Cohort Studies [Available from: https://www.evidencepartners.com/wp-content/uploads/2021/03/Tool-to-Assess-Risk-of-Bias-in-Cohort-Studies-DistillerSR.pdf.

For peer review only

Marks et al. [43]	definitely yes	definitely yes	definitely yes	definitely no	definitely no	probably no	definitely yes	probably no
Butt et al. [39]	probably yes	definitely yes	probably yes	probably yes	probably yes	definitely yes	definitely no	probably yes
Kildegaard et al. [33]	probably yes	probably yes	probably yes	definitely no	probably no	definitely yes	definitely yes	probably yes
Fowlkes et al. [40]	definitely yes	probably yes	probably yes	probably no	definitely no	probably yes	probably yes	probably yes
Hao et al. [24]	probably yes	probably yes	probably yes	probably no	definitely no	probably yes	probably yes	probably yes
Brewster et al. [37]	definitely yes	definitely yes	definitely yes	definitely no	definitely yes	definitely yes	probably yes	probably no
Waltenburg et al. [17]	probably yes	probably yes	probably yes	probably no	probably no	probably yes	definitely no	probably yes
Marks et al. [46]	probably yes	probably no	probably yes	probably no	probably no	definitely yes	probably yes	probably yes
Nygaard et al. [25]	probably yes	probably yes	definitely yes	probably no	definitely no	definitely yes	definitely yes	probably yes

**Case control studies**[2]	**Confidence in exposure assessment**	**Confidence in cases but not controls acquiring outcome of interest**	**Proper selection of cases (those that were exposed and developed outcome of interest)**	**Proper selection of controls (those that were exposed and did not develop outcome of interest)**	**Matching of cases and controls according to prognostic variables or statistical adjustment for these variables**
Roberts et al. [14]	probably yes	probably yes	definitely yes	probably yes	definitely no

[2] CLARITY Group at McMaster University. Tool to Assess Risk of Bias in Case Control Studies [Available from: https://www.evidencepartners.com/wp-content/uploads/2021/03/Tool-to-Assess-Risk-of-Bias-in-Case-Control-Studies-DistillerSR.pdf.

For peer review only

Cross sectional studies	Exposed and non-exposed groups from the same population	Confidence in exposure assessment	Confidence that outcome of interest was not present at start of study	Matching of exposed and unexposed for all variables associated with outcome of interest or adjustment of prognostic variables by statistical analysis	Confidence in assessment of presence/absence of prognostic factors	Confidence in outcome assessment	Similar co-interventions between groups
Iuliano et al. [44]	probably yes	probably yes	definitely yes	definitely no	definitely no	definitely yes	probably no
Shi et al. [45]	definitely yes	definitely yes	probably yes	definitely no	definitely yes	definitely yes	probably yes
Somekh et al. [16]	probably yes	definitely yes	probably no	definitely no	probably no	definitely yes	probably yes

For peer review only

Supplementary table 3: Risk of bias assessment - Child/Adolescent risk of severe disease when infected with a VOC

**Cohort studies[1]**	**Exposed and non-exposed cohorts from the same population**	**Confidence in exposure assessment**	**Confidence that outcome of interest was not present at start of study**	**Matching of exposed and unexposed for all variables associated with outcome of interest or adjustment of prognostic variables by statistical analysis**	**Confidence in assessment of presence/absence of prognostic factors**	**Confidence in outcome assessment**	**Adequate follow-up of cohorts**	**Similar co-interventions between groups**
Butt et al. [39]	probably yes	definitely yes	probably yes	probably yes	probably yes	definitely yes	definitely no	probably yes

**Cross sectional studies**	**Exposed and non-exposed groups from the same population**	**Confidence in exposure assessment**	**Confidence that outcome of interest was not present at start of study**	**Matching of exposed and unexposed for all variables associated with outcome of interest or adjustment of prognostic variables by statistical analysis**	**Confidence in assessment of presence/absence of prognostic factors**	**Confidence in outcome assessment**	**Similar co-interventions between groups**
Shi et al. [45]	definitely yes	definitely yes	probably yes	definitely no	definitely yes	definitely yes	probably yes

For peer review only

[1] CLARITY Group at McMaster University. Tool to Assess Risk of Bias in Cohort Studies [Available from: https://www.evidencepartners.com/wp-content/uploads/2021/03/Tool-to-Assess-Risk-of-Bias-in-Cohort-Studies-DistillerSR.pdf.

Supplementary table 4: Risk of bias assessment - Child/Adolescent risk of becoming infected with a VOC

Cohort studies[1]	Exposed and non-exposed cohorts from the same population	Confidence in exposure assessment	Confidence that outcome of interest was not present at start of study	Matching of exposed and unexposed for all variables associated with outcome of interest or adjustment of prognostic variables by statistical analysis	Confidence in assessment of presence/absence of prognostic factors	Confidence in outcome assessment	Adequate follow-up of cohorts	Similar co-interventions between groups
Nakel et al. [18]	probably yes	definitely yes	probably yes	definitely no	definitely no	probably no	definitely no	probably yes
Somekh et al. [19]	probably yes	definitely yes	probably yes	probably no	probably no	definitely yes	probably yes	probably no
Abu Raddad et al. [54]	definitely yes	definitely yes	probably no	definitely no	probably no	probably yes	definitely yes	probably no
Loenenbach et al. [52]	probably yes	probably yes	probably no	definitely no	definitely no	definitely yes	probably no	probably no
Lorthe et al. [53]	definitely yes	definitely yes	probably yes	definitely no	probably yes	probably yes	definitely yes	definitely yes
Clifford et al. [55]	probably yes	probably yes	definitely yes	definitely no	definitely no	definitely yes	probably no	probably yes
Dougherty et al. [57]	probably yes	definitely yes	definitely yes	probably no	probably yes	definitely yes	probably yes	probably no
Singanayagam et al. [58]	probably yes	probably yes	probably yes	probably no	probably no	definitely yes	probably yes	probably no
Schenk et al. [50]	probably yes	probably yes	probably no	probably no	probably no	probably yes	probably no	probably yes
Lorthe et al. [59]	probably yes	definitely yes	definitely no	definitely no	definitely no	definitely yes	probably no	definitely no
Waltenburg et al. [17]	probably yes	probably yes	probably yes	probably no	probably no	probably yes	definitely no	probably yes
Ng et al. [56]	probably yes	probably yes	probably yes	probably no	definitely no	probably yes	probably yes	probably yes

[1] CLARITY Group at McMaster University. Tool to Assess Risk of Bias in Cohort Studies [Available from: https://www.evidencepartners.com/wp-content/uploads/2021/03/Tool-to-Assess-Risk-of-Bias-in-Cohort-Studies-DistillerSR.pdf.

For peer review only

Case control studies[2]	Confidence in exposure assessment	Confidence in cases but not controls acquiring outcome of interest	Proper selection of cases (those that were exposed and developed outcome of interest)	Proper selection of controls (those that were exposed and did not develop outcome of interest)	Matching of cases and controls according to prognostic variables or statistical adjustment for these variables
Roberts et al. [14]	probably yes	probably yes	definitely yes	probably yes	definitely no

Cross sectional studies	Exposed and non-exposed groups from the same population	Confidence in exposure assessment	Confidence that outcome of interest was not present at start of study	Matching of exposed and unexposed for all variables associated with outcome of interest or adjustment of prognostic variables by statistical analysis	Confidence in assessment of presence/absence of prognostic factors	Confidence in outcome assessment	Similar co-interventions between groups
Somekh et al. [16]	probably yes	definitely yes	probably no	definitely no	probably no	definitely yes	probably yes
Neuberger et al. [51]	probably yes	probably yes	probably yes	probably no	probably no	probably yes	probably yes

[2] CLARITY Group at McMaster University. Tool to Assess Risk of Bias in Case Control Studies [Available from: https://www.evidencepartners.com/wp-content/uploads/2021/03/Tool-to-Assess-Risk-of-Bias-in-Case-Control-Studies-DistillerSR.pdf.

For peer review only

Supplementary table 5: Risk of bias assessment - Child/adolescent risk of transmission when infected with a VOC

Cohort studies[1]	Exposed and non-exposed cohorts from the same population	Confidence in exposure assessment	Confidence that outcome of interest was not present at start of study	Matching of exposed and unexposed for all variables associated with outcome of interest or adjustment of prognostic variables by statistical analysis	Confidence in assessment of presence/absence of prognostic factors	Confidence in outcome assessment	Adequate follow-up of cohorts	Similar co-interventions between groups
Somekh et al. [19]	probably yes	definitely yes	probably yes	probably no	probably no	definitely yes	probably yes	probably no
Buchan et al. [65]	definitely yes	probably yes	probably no	definitely no	definitely no	probably yes	probably no	probably no
Lindstrom et al. [60]	definitely yes	definitely yes	definitely no	probably no	definitely no	probably yes	probably no	probably yes
Loenenbach et al. [52]	probably yes	probably yes	probably no	definitely no	definitely no	definitely yes	probably no	probably no
Lorthe et al. [53]	definitely yes	definitely yes	probably yes	definitely no	probably yes	probably yes	definitely yes	definitely yes
Julin et al. [63]	probably yes	definitely yes	definitely yes	probably no	definitely no	definitely yes	definitely yes	probably yes
Lyngse et al. [61]	definitely yes	definitely yes	definitely yes	probably yes	probably yes	probably yes	probably yes	probably no
Chudasama et al. [62]	probably yes	probably no	probably no	definitely no	definitely no	probably yes	probably no	definitely no
Lorthe et al. [59]	probably yes	definitely yes	definitely no	definitely no	definitely no	definitely yes	probably no	definitely no
Trobajo-Sanmartin et al. [68|	probably yes	definitely yes	probably yes	probably yes	probably yes	definitely yes	probably no	probably yes
Waltenburg et al. [17]	probably yes	probably yes	probably yes	probably no	probably no	probably yes	definitely no	probably yes
Ng et al. [56]	probably yes	probably yes	probably yes	probably no	definitely no	probably yes	probably yes	probably yes

[1] CLARITY Group at McMaster University. Tool to Assess Risk of Bias in Cohort Studies [Available from: https://www.evidencepartners.com/wp-content/uploads/2021/03/Tool-to-Assess-Risk-of-Bias-in-Cohort-Studies-DistillerSR.pdf.

For peer review only

Case control studies[2]	Confidence in exposure assessment	Confidence in cases but not controls acquiring outcome of interest	Proper selection of cases (those that were exposed and developed outcome of interest)	Proper selection of controls (those that were exposed and did not develop outcome of interest)	Matching of cases and controls according to prognostic variables or statistical adjustment for these variables
Loss et al. [64]	probably yes	definitely yes	definitely yes	definitely yes	probably no
Allen et al. [67]	definitely yes	definitely yes	definitely yes	definitely yes	probably yes

Modelling studies[3]	Is the population relevant?	Are any critical interven-tions missing?	Are any relevant out-comes missing?	Is the context (settings and circum-stances) applicable?	Is external validation of the model sufficient to make its results credible for your decision?	Is internal verification of the model sufficient to make its results credible for your decision?	Does the model have sufficient face validity to make its results credible for your decision?	Is the design of the model adequate for your decision problem?	Are the data used in populating the model suitable for your decision problem?	Were the analyses performed using the model adequate to inform your decision problem?	Was there an adequate assessment of the effects of uncertainty?	Was the reporting of the model adequate to inform your decision problem?	Was the interpre-tation of results fair and balanced?	Were there any potential conflicts of interest?	If there were potential conflicts of interest, were steps taken to address these?
Ratman et al. [66]	definitely yes	probably yes	probably no	probably yes	definitely no	definitely no	probably no	probably yes	probably yes	probably yes	definitely no	probably yes	probably yes	definitely no	probably no

[2] CLARITY Group at McMaster University. Tool to Assess Risk of Bias in Case Control Studies [Available from: https://www.evidencepartners.com/wp-content/uploads/2021/03/Tool-to-Assess-Risk-of-Bias-in-Case-Control-Studies-DistillerSR.pdf.
[3] Jaime Caro J, Eddy DM, Kan H, Kaltz C, Patel B, Eldessouki R, et al. Questionnaire to assess relevance and credibility of modeling studies for informing health care decision making: an ISPOR-AMCP-NPC Good Practice Task Force report. Value Health. 2014;17(2):174-82. doi: 10.1016/j.jval.2014.01.003.

For peer review only

Supplementary table 6: Risk of bias assessment - Risk of long-term effects when infected with a VOC

Cohort studies[7]	Exposed and non-exposed cohorts from the same population	Confidence in exposure assessment	Confidence that outcome of interest was not present at start of study	Matching of exposed and unexposed for all variables associated with outcome of interest or adjustment of prognostic variables by statistical analysis	Confidence in assessment of presence/absence of prognostic factors	Confidence in outcome assessment	Adequate follow-up of cohorts	Similar co-interventions between groups
Molteni et al. [28]	definitely yes	probably yes	probably yes	probably yes	probably no	probably yes	probably yes	probably no
Kildegaard et al. [33]	probably yes	probably yes	probably yes	definitely no	probably no	definitely yes	definitely yes	probably yes

[7]CLARITY Group at McMaster University. Tool to Assess Risk of Bias in Cohort Studies [Available from: https://www.evidencepartners.com/wp-content/uploads/2021/03/Tool-to-Assess-Risk-of-Bias-in-Cohort-Studies-DistillerSR.pdf.

For peer review only

Supplementary table 7: Review question 1 - Child/adolescent symptoms/severity of disease when infected with the alpha variant (n=9)

Author	Study design	Country	Study setting	Number of study participants <20 yo[1]	Number of adult study participants	Age groups	Mean/ Median age	VOC[2]	Symptoms/Severity	Comments
Roberts et al. [14]	case-control study	United Kingdom	schools kindergartens crèches	overall: 83<br><br>infected: 21	34	≤4yo, adults	n/r	Alpha	**Symptoms**<br>24% fever, 19% cough<br>(52% asymptomatic)	**sequencing:** 1 case was sequenced, rest was assumed based on date of sampling and national prevalence
Ulyte et al. [15]	cohort study	Switzerland	schools kindergartens crèches	overall: 2 487<br><br>newly infected: 182	n/a	7-17 yo	median: 12 yo	Alpha	**Symptoms**<br>17% fatigue (30/182), 17% sore throat (30/182), 15% headache (27/182), 14% fever (26/182)	**sequencing:** n/r
Somekh et al. [16]	cross-sectional study	Israel	households	alpha infection: 31<br><br>non-VOC infection: 20	n/a	11-17 yo	n/r	Alpha	**Symptoms (alpha vs. non-VOC)**<br>sensory impairment: 6.5% (2/31) vs. 40% (8/20)<br>(RR[3]: 0.16; 95%CI[4]: 0.04–0.6)	**sequencing:** n/r

[1] yo: years old
[2] VOC: Variants Of Concern
[3] RR: Risk Ratio
[4] 95%CI: 95% Confidence Interval

For peer review only

Waltenburg et al. [17]	cohort study	USA[5]	households	n/r	n/r	<5 yo, 5-11 yo, 12-17 yo	median: 12 yo (IQR[6] 7-15)	Alpha	**Symptoms (alpha vs. non-VOC)**<br>constitutional (chills, fever, myalgia): OR[7] 5.5 (95%CI: 1.55-19.20)<br>lower respiratory (e.g. cough): OR 3.52 (95%CI: 1.26-9.86)<br>gastrointestinal (e.g. diarrhoea, pain): OR 3.55 (95%CI: 1.16-10.89)<br>**Severity (alpha vs. non-VOC):**<br>Symptom duration (median, days): 3 vs. 2, p[8] = 0.64	**vaccination status:** 97% unvaccinated (147/151), 3% partially vaccinated (4/151)<br>**sequencing:** majority of primary cases sequenced
Nakel et al. [18]	cohort study	Germany	schools kindergartens crèches	infected: 40<br>alpha infection: 18<br>non-VOC infection: 22	n/a	≤6 yo	n/r	Alpha	**Symptoms (alpha vs. non-VOC)**<br>fever: 38.9% (7/18) vs. 50% (11/22)<br>cough: 44.4% (8/18) vs. 36.4% (8/22)<br>shortness of breath: 11.1% (2/18) vs. 0% (0/22)<br>**Severity (alpha vs. non-VOC)**<br>ICU[9] admission: 0 vs. 0	**sequencing:** partially
Somekh et al. [19]	cohort study	Israel	hospital inpatients hospital outpatients	alpha infection: 72 796<br>non-VOC infection: 26 689	n/a	≤9 yo	n/r	Alpha	**Severity (alpha vs. non-VOC)**<br>Hospitalisation: 0.52% (379/72796) vs. 0.98% (261/26689)<br>RR 0.53 (95% CI: 0.46-0.63), p < 0.001<br>Severe disease/death: 6.9% (26/379) vs. 6.5% (17/261)<br>RR 0.99 (95% CI: 0.96-1.04)	**sequencing:** n/r
Swann et al. [20]	cohort study	United Kingdom	hospital inpatients	infected: 1 617<br>alpha infection: 952<br>non-VOC infection: 665	n/a	≤19 yo	median (alpha): 6.5 yo (IQR 0.3-14.9)<br>median (non-VOC): 4.0 yo (IQR 0.4-13.6)	Alpha	**Symptoms (alpha vs. non-VOC)**<br>fever: 57.1% (544/952) vs. 73.8% (491/665), p<0.001<br>**Severity (alpha vs. non-VOC)**<br>Length of hospitalisation: 2 days (IQR 1-4) vs. 2 days (IQR 1-4), p=0.079<br>critical care: 12.0% (109/910) vs. 12.7 (78/616), p=0.989	**sequencing:** n/r

[5] USA: United States of America
[6] IQR: Interquartile Range
[7] OR: Odd Ratio
[8] p: p-value
[9] ICU: Intensive Care Unit

For peer review only

Brookman et al. [21]	cohort study	United Kingdom	hospital outpatients	infected: 80<br><br>alpha infection: 60<br><br>non-VOC infection: 20	n/a	≤18 yo	n/r	Alpha	**Severity (alpha vs. non-VOC)**<br>Critical disease: 3% (2/60) vs. 20% (4/20)<br>Oxygen: 8% (5/60) vs. 35% (7/20)<br>non-invasive ventilation: 3% (2/60) vs. 15% (3/20)<br>invasive ventilation: 2% (1/60) vs. 20% (4/20)	**sequencing:** n/r
Stewart et al. [22]	cohort study	United Kingdom	hospital inpatients	infected: 110<br><br>alpha infection: 67<br>non-VOC infection: 43	n/a	<18 yo	median overall: 10.2 yo (IQR 7.6-12.6)	Alpha	**Symptoms (alpha vs. non-VOC)**<br>PIMS-TS[10]: 67 vs. 43 cases<br>acute kidney injury in PIMS-TS:<br>28.4% (19/67) vs. 33% (14/43), p=0.85	**sequencing:** n/r

[10] [6]PIMS-TS/MIS-C: Paediatric Inflammatory Multisystem Syndrome/Multisystem Inflammatory Syndrome in Children

Supplementary table 8: Review question 1 - Child/adolescent symptoms/severity of disease when infected with the delta variant (n=13)

Author	Study design	Country	Study setting	Number of study participants <20 yo[1]	Number of adult study partici-pants	Age groups	Mean/ Median age	VOC[2]	Symptoms/Severity	Comments
Li et al. [23]	cohort study	China	hospital inpatients	infected: 77	n/a	<12 yo	median: 9 yo	Delta	**Symptoms**<br>75.3% fever (58/77), 18.2% cough (14/77), 7.8% expectoration (6/77)	**sequencing:** all everyone was hospitalised for isolation
Hao et al. [24]	cohort study	China	hospital inpatients	infected: 66	n/a	≤18 yo	mean (±SD[3]): 10.35±5.86 yo	Delta	**Symptoms**<br>47.0% fever (31/66), 47.0% cough (31/66), 34.8% nasal congestion (23/66), 25.8% sore throat (17/66), 10.6% fatigue (7/66), 4.5% decreased sense of smell (3/66)	**Sequencing:** n/r
Nygaard et al. [25]	cohort study	Denmark	hospital inpatients	infected: 74<br><br>delta infection : 51<br><br>non-VOC infection: 23	n/a	≤17 yo	n/r	Delta	**MIS-C[4] symptoms (delta vs. non-VOC)**<br>hypotension/shock: 51% (26/51) vs. 57% (13/23)<br>cardiac involvement: 90% (46/51) vs. 100% (23/23)<br>gastrointestinal involvement: 98% (50/51) vs. 100% (23/23)<br>respiratory involvement: 12% (6/51) vs. 26% (6/23)<br><br>**MIS-C severity (delta vs. non-VOC)**<br>ICU[5] admission: 55% (28/51) vs. 52% (12/23)<br>vasoactive support: 16% (8/51) vs. 22% (5/23)<br>no need for mechanical ventilation or ECMO[6], no deaths for both delta and non-VOC infection<br><br>(risk of MIS-C comparable with delta and non-VOC infection)	**vaccination status:** none vaccinated<br><br>**sequencing:** majority sequenced

[1] yo: years old
[2] VOC: Variants Of Concern
[3] SD: Standrad Deviation
[4] MIS-C: Multisystem Inflammatory Syndrome in Children
[5] ICU: Intensive Care Unit
[6] ECMO: Extracorporeal Membrane Oxygenation

For peer review only

Murillo-Zamora et al. [26]	cohort study	Mexico	hospital inpatients	infected: 26 961<br><br>person-days: 297 099	n/r	≤19 yo	mean (±SD): 14.4±5.8 yo	Delta	**Severity (delta vs. pre-delta)**<br>pneumonia: incidence rate 30.3/10 000 person-days vs. 9.4/10 000 person-days<br>RR[7] 0.98 (95%CI: 0.97-0.99), p[8]<0.001	**sequencing:** n/r<br>no information on pre-delta strains
Ryu et al. [27]	cohort study	South Korea	population	infected: 235<br><br>delta infection: 92<br><br>pre-delta infection: 143	n/r	<18 yo	mean (delta): 10.2 yo<br><br>mean (pre-delta): 13.8 yo<br>p<0.001	Delta	**Symptoms (delta vs. pre-delta)**<br>rhinorrhoea: 10.5% (15/143) vs. 25% (23/92), p=0.003<br>nasal stuffiness: 15.4% (22/143) vs. 34.8% (32/92), p=0.001<br>sore throat: 12.6% (18/143) vs. 23.9% (22/143), p=0.02<br><br>no difference in prevalence of other COVID symptoms (e.g. fever, cough, pneumonia)	**sequencing:** n/r<br>no information on pre-delta strains
Fisman et al. [28]	cohort study	Canada	population	infected: 20 434<br><br>delta infection: 12 387<br><br>non-VOC infection: 8 047	n/a	≤19 yo	n/r	Delta	**Severity (delta vs. non-VOC)**<br>hospitalization (<10 yo): OR[9] 2.51 (95%CI: 1.23-5.11)<br>hospitalization (10-19 yo): OR 0.78 (95%CI: 0.38-1.91)<br><br>ICU admission (10-19 yo): OR 1.37 (95%CI: 0.11-16.46)	**sequencing:** all sequenced
Shoji et al. [29]	cohort study	Japan	hospital inpatients	infected: 1 299<br><br>delta infection: 349<br><br>pre-delta infection: 950	n/a	<18 yo	median (delta): 7.0 yo (IQR[10]: 2-13)<br><br>median (pre-delta): 10.0 yo (IQR: 4-15)<br>p<0.001	Delta	**Severity (delta vs. pre-delta)**<br>ICU admission: 1.4% (5/349) vs. 0.1% (1/950), p=0.006<br>length of hospitalisation (median, days): 7.0 (IQR 5.0-9.0) vs. 8.0 (IQR 5.0-9.0), p=0.031<br>mechanical ventilation: 0% vs. 0%	**sequencing:** n/r<br>no information on pre-delta strains

[7] RR: Risk Ratio
[8] p: p-value
[9] OR: Odd Ratio
[10] IQR: Interquartile Range

For peer review only

Martin et al. [30]	cohort study	USA[11]	hospital inpatients hospital outpatients	infected: 167 262<br><br>delta infection: 29 191<br><br>pre-delta infection: 138 071	n/a	<19 yo	median (delta): 10.3 yo (IQR 5.3-14.9)<br><br>median (pre-delta): 12.3 yo (IQR 6.2-16.4)	Delta	**Severity (delta vs. pre-delta)**<br>hospitalisation: 6.0% (1738/29191) vs. 6.2% (8507/138071), p=0.18<br>severe disease: 10.3% (179/1738) vs. 14.6% (1242/8507), (OR 0.67, 95%CI: 0.57-0.79), p<0.001<br>severe disease (children classified as non-black, non-white): OR 1.69 (95%CI: 1.02-2.82, p=0.04	**sequencing:** n/r<br>no information on pre-delta strains
Butt et al. [31]	cohort study	Qatar	population	infected: 246<br><br>delta infection: 125<br><br>beta infection: 121	n/a	≤19 yo	n/a	Beta, Delta	**Symptoms (delta vs. beta)**<br>moderate disease: aOR[12] 0.17 (0.06-0.48) vs. 0.35 (0.09-1.39), p=0.54<br>severe disease: aOR 0.08 (0.01-0.48) vs. NA, p>0.99	**sequencing:** all sequenced
Molteni et al. [32]	cohort study	United Kingdom	population	infected: 1 400<br><br>delta infection: 706<br>5-11 yo: 227<br>12-17 yo: 479<br><br>alpha infection: 694<br>5-11 yo: 276<br>12-17 yo: 418	n/r	5-17 yo	median (alpha): 12 yo (IQR 9-15)<br><br>median (delta): 14 yo (IQR 13-16)	Alpha, Delta	**Symptoms (delta vs. alpha)**<br>5-11yo<br>headache: 60.8% (138/227) vs. 39.9% (110/276), OR 2.31 (95%CI: 1.6-3.33), p<0.001<br>fever: 46.3% (105/227) vs. 31.9% (88/276), OR 1.89 (95%CI: 1.31-2.73) (p=0.001)<br>12-17yo<br>headache: 73.7% (353/479) vs. 61.5% (257/418), OR 1.81 (95%CI: 1.36-2.42), p<0.001<br>fever: 43.2% (207/479) vs. 27.0% (113/418), OR 2.05 (95%CI: 1.55-2.72), p<0.001<br>rhinorrhoea: 57.6% (276/479) vs. 47.4% (198/418), OR 1.52 (95%CI: 1.17-1.98), p=0.002<br>sore throat: 60.5% (290/479) vs. 45.7% (191/418), OR 1.87 (95%CI: 1.43-2.45), p<0.001<br><br>**Severity (delta vs. alpha)**<br>symptom burden (5-11 yo, median/days): 4 (IQR 2-7) vs 3 (IQR 2-5)<br>symptom burden (12-17 yo, median/days): 6 (IQR 3-9) vs 5 (IQR 3-8)	**sequencing:** n/r

[11] USA: United States of America
[12] aOR: Adjusted Odd Ratio

For peer review only

Kildegaard et al.<br>[33]	cohort study	Denmark	Hospital inpatients and outpatients	infected: 74 611<br><br>alpha infection: n/r<br><br>delta infection: n/r	n/a	<18 yo	Median (infected): 8 yo (IQR 1-14)	Alpha, Delta	**Severity (alpha vs. delta)**<br>hospitalization: RR[13] 0.49% (98/20102) (95%CI[14]: 0.4-0.59) vs. 0.48% (18/16694) (95%CI: 0.38-0.6)<br>MIS-C: RR 0.04% (9/20164) (95%CI: 0.02-0.08) vs. 0.04% (5/12778) (95%CI: 0.01-0.09)	**vaccination status:** partially vaccinated<br><br>**sequencing:** n/r
Çelebi et al.<br>[34]	cohort study	Turkey	hospital inpatients hospital outpatients	infected: 680<br><br>alpha infection: 329<br><br>beta/gamma infection: 17<br><br>delta infection: 165<br><br>non-VOC infection: 169	n/a	≤17 yo	median: 9.25 yo	Alpha, Beta, Delta	**Severity (alpha vs. delta vs. beta/gamma vs. non-VOC)**<br>hospitalization: 9.4% (31/329) vs. 19.4% (32/165) vs. 18% (3/17) vs. 10.1% (17/169)<br><br>**Severity (alpha vs. delta)**<br>hospitalization: 9.4% (31/329) vs. 19.4% (32/165), p=0.016<br><br>**Severity (delta vs. non-VOC)**<br>hospitalization: 19.4% (32/165) vs. 10.1% (17/169), p=0.002	**sequencing:** all sequenced, but no differentiation between gamma and beta

For peer review only

[13] RR: Risk Ratio
[14] 95%CI: 95% Confidence Interval

For peer review only - http://bmjopen.bmj.com/site/about/guidelines.xhtml

Edward et al. [35]	cohort study	USA	hospital outpatients	infected: 499<br><br>alpha infection: 96<br><br>gamma infection: 38<br><br>delta infection: 119<br><br>non-VOC infection: 243	n/a	≤18 yo	median (alpha): 9 yo (IQR 2-14)<br><br>median (gamma): 7 yo (IQR 1-14)<br><br>median (delta): 4 yo (IQR 1-11)<br><br>median (non-VOC): 7 yo (IQR 1-12)	Alpha, Delta, Gamma	**Symptoms (alpha vs. gamma vs. delta vs. non-VOC)**<br>symptomatic: 91.6% (88/96) vs. 89.4% (34/38) vs. 89.9% (107/119) vs. 91.7% (223/243), p=0.92<br><br>**Severity - Hospitalization**<br>non-VOC: 3.2% (8/243) reference<br>alpha: 7.2% (7/96): OR 1.9 (95%CI: 0.59-6.3)<br>gamma: 15.7% (6/38): OR 5.9 (95%: 1.6-21.5), p=0.007<br>delta: 6.7% (8/119): OR 2.2 (95%CI: 0.69-7.7)<br><br>**ICU admission**<br>non-VOC: 1.2% (3/243): reference<br>alpha: 1.0% (1/96) :OR 0.47 (95%CI: 0.02-4.0)<br>gamma: 5.2% (2/38): OR 2.6 (95%CI: 0.31-17.5)<br>delta: 2.5% (3/119): OR 1.4 (95%CI: 0.24-9.0)<br><br>**Respiratory support**<br>non-VOC: 1.2% (3/243): reference<br>alpha: 0% : OR 0<br>gamma: 10.5% (4/38) : OR 8.3 (95%CI: 1.5-56.3), p=0.02<br>delta: 2.5% (3/119) : OR 2.2 (95%CI: 0.34-16.9)	**sequencing:** all sequenced

For peer review only

Supplementary table 9: Review question 1 - Child/adolescent symptoms/severity of disease when infected with the omicron variant (n=11)

Author	Study design	Country	Study setting	Number of study participants <20 yo[1]	Number of adult study partici-pants	Age groups	Mean/ Median age	VOC[2]	Symptoms/severity	Comments
Cloete et al. [36]	cohort study	South Africa	hospital inpatients	infected: 139	n/a	≤13 yo	n/r	Omicron	**Symptoms**<br>47% fever, 40% cough, 24% vomiting, 23% difficulty breathing, 20% convulsions<br><br>**Severity**<br>hospitalization: 92% standard ward care, 25% oxygen therapy, 2% high flow oxygen, 6% ventilation	**sequencing:** partially sequenced
Brewster et al. [37]	cohort study	USA[3]	hospital inpatients, hospital outpatients	infected: 75<br><br>omicron: 61<br><br>pre-omicron: 14	n/a	n/r	median (omicron):<br>ED[4]: 2.4 yo (0.8–2.6)<br>hospitalized: 1.6 yo (0.7–1.1)<br><br>median (pre-omicron):<br>ED: 2.6 yo (1.6–3.4)<br>hospitalized: 1.3 yo (1.1–1.5)	Omicron	**Symptoms (omicron vs. pre-omicron):**<br>Croup: 81% (61/75) vs. 19% (14/75)<br><br>Median weekly croup cases (omicron vs. pre-omicron):<br>11 [IQR[5] 2–17] vs. 0 (IQR 0-0), p[6] < 0.001<br><br>none required invasive ventilation or died (irrespective of variant)	**sequencing:** n/r
Ludvigsson et al. [38]	case series (>1 case)	Sweden	hospital inpatients	infected: 3	n/r	<15 yo	n/r	Omicron	**Symptoms**<br>**Case 1:** repeated convulsions over several hours, hospitalisation: 4 days<br>**Case 2:** status epilepticus, hospitalisation: 2 days<br>**Case 3:** one convulsion (30-60sec), short behavioural change, hospitalisation: 2 days	**sequencing:** n/r<br>14yo: clinical diagnosis

[1] yo: years old
[2] VOC: Variants Of Concern
[3] USA: United States of America
[4] ED: Emergency Department
[5] IQR: Interquartile Range
[6] p: p-value

For peer review only

Butt et al. [39]	cohort study	Qatar	population	Infected: 985<br><br>(both the omicron and delta cohort)	n/r	<18 yo	median (delta): 7 yo<br>(IQR 3-9)<br><br>median (omicron): 6 yo<br>(IQR 3-10)	Delta, Omicron	**Severity (omicron vs. delta)**<br>mild/not hospitalised: 97.8% (963/985) vs. 84.2% (829/985), p<0.001<br>moderate disease: 2.2% (22/985) vs. 15.7% (155/985)<br>critical disease: 0% (0/985) vs. 0.1% (1/985)<br><br>moderate+critical disease: 2.2% (22.985) vs. 15.8% (156/985), p<0.001,<br>aOR[7] 0.12 (95%CI: 0.07-0.18)	**vaccination status:** none vaccinated<br><br>**sequencing:** n/r
Fowlkes et al. [40]	cohort study	USA	population	infected: 252<br><br>omicron infection: 150<br><br>delta infection: 102	n/r	5-11 yo, 12-15 yo	n/r	Delta, Omicron	**Symptoms (omicron vs. delta)**<br>COVID[8]- symptoms: 48.7% (73/150) vs. 65.7% (67/102); OR[9] 2.0 (95%CI[10]: 1.2-3.45), p=0.008'<br><br>**Severity (omicron vs. delta)**<br>total days of symptoms: 5.4% vs. 8.0%; OR -3.4 (95%CI: (-)5.7 - (-)1.0), p=0.006<br>hours of missed school: 21.8% vs. 24.1%; OR -10.6 (95%CI: (-)18.6 - (-)2.7), p=0.01<br><br>(no differences in febrile symptoms, received medical care, days spent sick in bed)	**vaccination status:**<br>5-11 yo: 65% (682/1052) double vaccinated, 7% (69/1052) 1 dose, and 29% (301/1052) unvaccinated.<br>12–15 yo: 68% (212/312) double vaccinated, 5% (15/312) 1 dose, 27% (85/312) unvaccinated<br><br>**sequencing:** n/r
Wang et al. [41]	cohort study	USA	hospital inpatients hospital outpatients	infected: 7 198<br><br>(both the omicron and the delta cohort)	n/r	<5 yo	mean (omicron): 1.49±1.42<br><br>mean (delta): 1.48±1.42	Delta, Omicron	**Severity (omicron vs. delta)**<br>ED visits: 18.83 %(1355/7198) vs. 26.67 (1920/7198); RR[11] 0.71 (95% CI: 0.66-0.75)<br>hospitalisation: 1.04% (75/7198) vs. 3.14 (226/7198); RR 0.33 (95%CI: 0.26-0.43)<br>ICU[12] admission: 0.14% (10/7198) vs. 0.43% (31/7198); RR 0.32 (95%CI: 0.16-0.66)<br>mech. ventilation: 0.33% (24/7198) vs. 1.15% (83/7198); RR 0.29 (95%CI: 0.18-0.46)	**sequencing:** n/r

For peer review only

[7] aOR: Adjusted Odd Ratio
[8] COVID: Coronavirus Disease
[9] OR: Odd Ratio
[10] 95%CI: 95% Confidence Interval
[11] RR: Risk Ratios
[12] ICU: Intensive Care Units

Wang et al. [42]	cohort study	USA	hospital inpatients hospital outpatients	infected ≤4 yo: 11 556 (both the omicron and the delta cohort)<br><br>infected 5-17 yo: 18 715 (both the omicron and the delta cohort)	n/a	≤4 yo, 5-17yo	n/a	Delta, Omicron	**Severity (omicron vs. delta)**<br>≤ 4yo<br>ED visits: 20.41% (2359/11556) vs. 23.96% (2796/1156); RR 0.85 (95%CI: 0.81-0.89)<br>hospitalization: 1.55% (179/11556) vs. 3.0% (347/11556); RR 0.52 (95%CI: 0.43-0.62)<br>ICU admissions: 0.1% (12/11556) vs. 0.42% (49/11556); RR 0.25 (95%CI: 0.13-0.46)<br>mech. ventilation: 0.09% (10/11556) vs. 0.2% (23/11556); RR 0.44 (95%CI: 0.21-0.91)<br><br>5-17yo<br>ED visits: 10.09% (1889/18715) vs. 13.07% (2446/18715); RR 0.77 (95%CI: 0.73-0.82)<br>hospitalization: 0.77% (144/18715) vs. 1.37% (144/18715);<br>RR 0.56 (95%CI: 0.46-0.69)<br>ICU admissions: 0.23% (43/18715) vs. 0.38% (71/18715); RR 0.61 (95%CI: 0.42-0.88)<br>mech. ventilation: 0.05% (10/18715) vs. 0.06 (11/18715); RR 0.91 (95%CI: 0.39-2.14)	**sequencing:** n/r<br>**vaccination status:** n/r
Marks et al. [43]	cohort study	USA	hospital inpatients	infected: 2 100<br><br>omicron infection: 266<br><br>delta infection: 1 834	n/r	≤4 yo, 5-11 yo, 12-17yo	median: 7 yo (IQR 1-14)	Delta, Omicron	**Severity (omicron vs. delta)**<br>ICU admission (≤17 yo): 20.2% (52/266) vs. 27.8% (510/1834)<br>mech. ventilation (≤17 yo): 2.3% (6/266) vs. 6.3% (112/1834)<br><br>hospitalization (≤4yo): 54.2% (142/266) vs. 42.5% (778/1834); RR 5.4 (95%CI: 4.0-7.2)<br>hospitalization (5-11 yo): 16.9% (43/266) vs. 22.5% (417/1834); RR 2.3 (95%CI: 1.5-3.6)<br>hospitalization (12–17 yo): 28.9% (81/266) vs. 34.9% (639/1834); RR 3.5 (95%CI: 2.5-5.0)	**sequencing:** n/r<br>**vaccination status:** n/r
Iuliano et al. [44]	cross-sectional study	USA	hospital inpatients	infected: 824<br><br>omicron infection: 405<br><br>delta infection: 272<br><br>non-VOC infection: 147	n/a	≤17 yo	n/r	Delta, Omicron	**Severity (omicron vs. delta)**<br>ICU admission: 10.4% (42/405) vs. 18.4% (50/272)<br>(ICU admission non-VOC: 17.0% (25/147))	**sequencing:** n/r<br>**vaccination status:** n/r

For peer review only

Shi<br>et al.<br>[45]	cross-sectional study	USA	hospital inpatients	infected:<br>1 475<br><br>omicron infection:<br>397<br><br>delta infection:<br>482<br><br>pre-Delta infection:<br>596	n/a	5-11 yo	median:<br>8 yo<br>(IQR 6-10)	Delta, Omicron	**Severity (omicron vs. delta)**<br>ICU admission: 18.9% vs. 26.1%, p=0.05<br>mech. ventilation: 4.6% vs. 6.8%, p=0.28<br>death: 0% vs. 0%<br><br>**Severity (omicron vs. pre-delta)**<br>ICU admission pre-Delta: 18.9% vs. 32.6%, p<0.01<br>mech. ventilation: 4.6% vs. 6.7%, p=0.28<br>death: 0%. vs. 0.7%<br><br>**underlying diseases (omicron vs. delta):**<br>neurologic disorders: 33% vs. 21%, p<0.01<br>obesity: 33% vs. 21%, p = 0.01	**sequencing:** n/r<br>**vaccination status:** n/r
Marks<br>et al.<br>[46]	cohort study	USA	Hospital inpatients	infected:<br>2 562<br><br>omicron infection:<br>572<br><br>delta infection:<br>790<br><br>pre-delta infection:<br>1 200	n/a	≤4 yo	median:<br>0.6yo<br>(IQR 0.1–1.0)	Delta, Omicron	**Severity (omicron vs. delta vs. pre-delta):**<br>length of hospitalisation (days, median, IQR): 1.5 (0.5-2.5) vs. 2 (1-3.5) vs. 1.5 (1-3.5),<br>p (omicron vs. pre-delta)= 0.001,<br>p (omicron vs. delta)=0.002<br>ICU admission: 21.0 % vs. 26.7% vs. 24%,<br>p (omicron vs. pre-delta)= 0.19,<br>p (omicron vs. delta)=0.02<br>BiPAP/CPAP[13]: 5.1% vs. 9.1% vs. 5.9%,<br>p (omicron vs. pre-delta)= 0.53,<br>p (omicron vs. delta)=0.008<br>high flow nasal canula: 13.4% vs. 20.4% vs. 8.3%, p (omicron vs. pre-delta)= 0.002,<br>p (omicron vs. delta)=0.002<br><br>No difference in invasive mechanical ventilation and in hospital deaths	**vaccination status:** none vaccinated<br><br>**sequencing:** n/r

[13] BiPAP/CPAP: Bilevel Positive Airway Pressure/ Continuous Positive Airway Pressure

For peer review only

Supplementary table 10: Review question 1 - Child/adolescent symptoms/severity of disease when infected with the gamma variant (n=2)

Author	Study design	Country	Study setting	Number of study participants <20 yo[1]	Number of adult study participants	Age groups	Mean/Median age	VOC[2]	Symptoms/Severity	Comments
Oliveira et al. [47]	cohort study	Brazil	hospital inpatients	infected: 21 591<br><br>gamma infection: 10 017<br><br>non-VOC infection: 11 574	n/r	<20 yo	mean (gamma): 4.0 yo (IQR[3] 0.6 - 14.7)<br><br>mean (non-VOC): 5.2 yo (IQR 1.0 - 14.4)	Gamma	**Severity (gamma vs. non-VOC)**<br>hypoxemia: 52.5% vs. 41.1%, p[4]<0.0001<br>ICU[5] admissions: 28.3% (2498/8830) vs. 24.9% (2745/11037), p<0.0001<br>invasive vent. support: 10.1% (974/9659) vs. 10.8% (1156/10733) p<0.0001	**sequencing:** n/r

[1] yo: years old
[2] VOC: Variant Of Concern
[3] IQR: Interquartile Range
[4] p: p-value
[5] ICU: Intensive Care Unit

For peer review only - http://bmjopen.bmj.com/site/about/guidelines.xhtml

Edward et al. [48]	cohort study	USA[6]	hospital outpatients	infected: 499<br><br>alpha infection: 96<br><br>gamma infection: 38<br><br>delta infection: 119<br><br>non-VOC infection: 243	n/a	≤ 18 yo	median (alpha): 9 yo (IQR 2-14)<br><br>median (gamma): 7 yo (IQR 1-14)<br><br>median (delta): 4 yo (IQR 1-11)<br><br>median (non-VOC): 7 yo (IQR 1-12)	Alpha, Delta, Gamma	**Symptoms (alpha vs. gamma vs. delta vs. non-VOC)**<br>symptomatic: 91.6% (88/96) vs. 89.4% (34/38) vs. 89.9% (107/119) vs. 91.7% (223/243), p=0.92<br><br>**Severity - Hospitalization**<br>non-VOC: 3.2% (8/243) reference<br>alpha: 7.2% (7/96): OR[7] 1.9 (95%CI[8]: 0.59-6.3)<br>gamma: 15.7% (6/38): OR 5.9 (95%: 1.6-21.5), p=0.007<br>delta: 6.7% (8/119): OR 2.2 (95%CI: 0.69-7.7)<br><br>**ICU admission**<br>non-VOC: 1.2% (3/243): reference<br>alpha: 1.0% (1/96): OR 0.47 (95%CI: 0.02-4.0)<br>gamma: 5.2% (2/38): OR 2.6 (95%CI: 0.31-17.5)<br>delta: 2.5% (3/119): OR 1.4 (95%CI: 0.24-9.0)<br><br>**Respiratory support**<br>non-VOC: 1.2% (3/243): reference<br>alpha: 0%: OR 0<br>gamma: 10.5% (4/38): OR 8.3 (95%CI: 1.5-56.3), p=0.02<br>delta: 2.5% (3/119) OR 2.2 (95%CI: 0.34-16.9)	**sequencing:** all sequenced

[6] USA: United States of America
[7] OR: Odds Ratio
[8] 95%CI: 95% Confidence Interval

For peer review only

Supplementary table 11: Review question 3 - Child/Adolescent risk of becoming infected with a SARS-CoV-2 variant of concern (n=15)

Author	Study design	Country	Study setting	Number of contacts <20 yo[1]	Number of adult contacts	Age groups	Mean/ Median age	VOC[2]	Risk of infection compared to non-VOCs/other VOCs	Risk of infection compared to adults	Comments
Schenk et al. [50]	cohort study	Germany	schools kindergartens crèches	non-VOC: 577<br><br>alpha: 756	non-VOC: 334 alpha: 226	≤7 yo, adults	median: 4yo	Alpha	**Risk of infection (alpha vs. non-VOC)** 0% (0/756) vs. 1.2% (7/577)	**Risk of infection (children vs. adults)** 0% (0/756) vs. 0% (0/226)	**vaccination status:** n/r **sequencing:** n/r
Neuberger et al. [51]	cross-sectional study	Germany	schools kindergartens crèches	n/r 8500 early childhood education and care centres	n/r	≤2 yo, 4-6 yo, ≥7 yo, adults	n/r	Alpha	n/a	**Risk of infection (children vs. adults)** higher absolute case number, but overall rate of infection still higher in adults	**vaccination status:** n/r **sequencing:** n/r
Nakel et al. [18]	cohort study	Germany	schools kindergartens crèches	113	31	≤6 yo	n/r	Alpha	**Risk of infection (alpha vs. non-VOC)** 15.9% (18/113) vs. 64.7% (22/34)	**Risk of infection (children vs. adults)** 15.9% (18/113) vs. 35.5% (11/31)	**vaccination status:** n/r **Sequencing:** few cases sequenced, presence of variant assumed for the rest based on date of sampling and national prevalence
Roberts et al. [14]	case-control study	United Kingdom	schools kindergartens crèches	83	34	≤4 yo, adults	n/r	Alpha	n/a	**Risk of infection AR[3] (children vs. adults)** 25.3% (21/83) vs. 70.6% (24/34)<br><br>**AR (children):**<br>- 0-1yo: 44%<br>- 1-2 yo: 31%<br>- 2-3yo: 31%<br>- 3-4 yo: 4%	**vaccination status:** n/r **sequencing:** 1 case sequenced, presence of variant assumed for the rest based on date of sampling and national prevalence

[1] yo: years old
[2] VOC: Variant Of Concern
[3] AR : Attack Rate

For peer review only

Loenenbach et al. [52]	cohort study	Germany	schools kindergartens crèches	133	60	≤6 yo, adults	n/r	Alpha	n/a	**Risk of infection SAR[4] overall (children vs. adults)** 21.1% (28/133) vs. 31.7% (19/60)<br><br>**SAR per daycare centre (children vs. adults)** 1. 31% (15/49) vs. 53% (10/19) 2. 27% (7/26) vs. 28% (5/18) 3. 17% (6/36) vs. 17% (4/23)	**vaccination status:** n/r **sequencing:** n/r
Lorthe et al. [53]	cohort study	Switzerland	schools kindergartens crèches	70	9	4-6 yo, adults	n/r	Alpha	n/a	**Risk of infection AR (children vs. adults)** 27.4% (22/70) vs. 22.2% (2/9)<br><br>**AR (children by classes)** 1. 62.9% (13/21) 2. 10.5% (2/19) 3. 16.7% (3/18) 4. 13.3% (2/15)	**vaccination status:** n/r **sequencing:** all cases
Abu-Raddad et al. [54]	cohort study	Qatar	population	55 638<br><br><10 yo: 28 791<br><br>10-19 yo: 26 847	30-39yo: 81 760	<10 yo, 10-19 yo, adults	n/r	Alpha	n/a	**Risk of infection (children <10 yo vs. adults)** 1.7% (502/28791) vs. 3.6% (2966/81760) OR[5] 0.35 (95%CI[6]: 0.32–0.39)<br><br>**Risk of infection (adolescents 10-19 yo vs. adults)** 3.6% (965/26847) vs. 3.6% (2966/81760)	**vaccination status:** n/r **sequencing:** 85% of all cases sequenced

[4] SAR: Secondary Attack Rate
[5] OR: Odd Ratio
[6] 95%CI: 95% Confidence Interval

For peer review only

Somekh et al. [19]	cohort study	Israel	hospital inpatients hospital outpatients	72 426<br><br>Alpha: 50 811<br><br>non-VOC: 21 615	n/r	<10 yo	n/r	Alpha	**Risk of infection (alpha vs. non-VOC)** slope of weekly adjusted incidence: 84.4 (95%CI: 71.1- 97.7) vs 39.1 (95%CI:23.9-54.3)	n/a	**sequencing:** n/r
Somekh et al. [16]	cross-sectional study	Israel	households	Alpha: 77<br><br>non-VOC: 58	n/r	≤5 yo, 6-17 yo	n/r	Alpha	**Risk of infection (alpha vs. non-VOC)** ≤5 yo: 72% (18/25) vs. 11.1% (2/18) RR[7] 7.8 (95%CI: 2.1–29.0)<br><br>6-17 yo: 75% (39/52) vs. 32.5% (13/40) RR 2.5 (95%CI: 1.6–4.0)	n/a	**sequencing:** n/r
Waltenburg et al. [17]	cohort study	USA[8]	households	Alpha : 61<br><br>non-VOC : 39	n/r	≤11 yo, 12-17 yo	Median : 12 yo (IQR[9] 7-15)	Alpha	**Risk of infection (alpha vs. non-VOC)** 64% (39/61) (95%CI: 51-76) vs. 56% (22/39) (95%CI: 40-72); OR 1.08 (95% CI: 0.40- 2.98)<br><br>**(≤11 yo vs. 12-17yo)** 78% (21/27) (95%CI: 58-91) vs. 53% (18/34) (95%CI: 35-70); OR 1.37 (95%CI:0.61-3.04)	n/a	**vaccination status:** 97% unvaccinated (147/151), 3% partially vaccinated (4/151) **sequencing:** majority of primary cases sequenced
Clifford et al. [55]	cohort study	United Kingdom	households	67	406	<18 yo, adults	n/r	Delta	n/a	**Risk of infection (children vs. adults)** RR 0.84 (95%CI: 0.66-0.99)<br><br>(risk of being infected by non-elderly adults)	**vaccination status:** n/r **sequencing:** 63.1% of cases sequenced, rest was assumed based on date of sampling and national prevalence

[7] RR: Risk Ratio
[8] USA: United States of America
[9] IQR: Interquartile Range

For peer review only

Ng et al. [56]	cohort study	Singapore	households	1 136	7223	≤11 yo, 12-17 yo, adults	median : 36 yo (IQR 26-51)	Delta	n/a	**Risk of infection SAR (≤11 yo vs. 18-29 yo)** 22.9% (173/757) vs. 12.0% (212/1765) ; aOR[10] 1.43 (95%CI: 1.07-1.93), p=0.017 **SAR (12-17 yo vs. 18-29 yo)** 12.9% (49/379) vs. 12.0% (212/1765) ; aOR 0.97 (95%CI: 0.66-1.42), p[11]=0.87	**vaccination status:** >12 yo partially vaccinated (results adjusted for vaccination status) **sequencing:** partially sequenced
Dougherty et al. [57]	cohort study	USA	camps, sport clubs	122	11	<12 yo, 12-19 yo, adults	median: 14 yo (IQR 5-58)	Delta	n/a	**Risk of infection AR (children vs. adults)** 18.9% (23/122) vs. 27.3% (3/11)	**vaccination status:** majority unvaccinated/partially vaccinated **sequencing:** 21 sequenced, rest was assumed based on date of sampling and national prevalence
Singana-yagam et al. [58]	cohort study	United Kingdom	community	30	198	5-18 yo, adults	median: 41 yo (IQR 28-49)	Delta	n/a	**Risk of infection SAR (children vs. adults)** 40% (12/30) vs. 20.1% (41/198)	**vaccination status:** 70% were fully vaccinated, 23.7% were partially vaccinated, 23% were not vaccinated **sequencing:** partially sequenced, rest was assumed based on date of sampling and national prevalence

[10] aOR: adjusted Odd Ratio
[11] p: p-value

For peer review only

For peer review only - http://bmjopen.bmj.com/site/about/guidelines.xhtml

Lorthe et al. [59]	Cohort study	Switzerland	schools kindergartens crèches	59	10	3-7 yo, adults	n/r	Omicron		**Risk of infection AR (children vs. adults)** 44.1% (26/59) vs. 50% (5/10)<br><br>**AR (children by classes)**<br>1. 33.3% (4/12)<br>2. 15.4% (2/13)<br>3. 56.3% (9/16)<br>4. 61.1% (11/18)	**vaccination status:** n/r<br>**sequencing:** none

For peer review only

Supplementary table 12: Review question 4 - Risk of child/adolescent transmission when infected with a SARS-CoV-2 variant of concern (n=15)

Author	Study design	Country	Study setting	Number of index cases <20 yo[1]	Number of adult index cases	number of contacts of children/ adolescents	number of contacts of adults	Age groups	Mean/ Median age	VOC[2]	Risk of transmission compared to non-VOCs/other VOCs	Risk of transmission compared to adults	Comments
Lindstrom et al. [60]	cohort study	Norway	population	42<br><br>alpha: 14<br><br>non-VOC: 28	373<br><br>alpha: 131<br><br>non-VOC: 242	mean (SD[3]): 2.79 (1.63)	40-59yo: 1.72 (1.87)<br><br>20-39yo: 1.03 (1.36)<br><br>>60yo: 1.25 (1.65)	<20 yo, adults	n/r	Alpha	n/a	**Transmission risk SAR[4] (<20 yo vs. 40-59 yo)** ref. vs. 1.97 (1.08-3.87)<br><br>no significant difference to adults 20-39 yo, >60yo	**vaccination status:** n/r **sequencing:** primary cases sequenced, variants in contacts assumed based on date of sampling and national prevalence
Lyngse et al. [61]	cohort study	Denmark	households	145<br><br><10 yo: 54<br><br>10-19 yo: 91	663	383<br><br><10yo: 141<br><br>10-19yo: 242	1 336	≤10 yo, 10-20 yo, adults	n/r	Alpha	n/a	**Transmission risk AR[5] (≤20 yo vs. adults)** 32.4% (124/383) vs. 39.8% (532/1336)<br><br>**AR (≤10 yo vs. 10-19 yo vs. adults)** 45% (64/141) vs. 25% (60/242) vs. 39.8% (532/1336)	**vaccination status:** n/r **sequencing:** 75% sequenced, rest was assumed based on date of sampling and national prevalence

For peer review only

[1] yo: years old
[2] VOC: Variant Of Concern
[3] SD: Standard Deviation
[4] SAR: secondary attack rate
[5] AR: attack rate

Chudasama et al. [62]	cohort study	United Kingdom	households	6 011<br><br><10 yo: 1 289<br><br>10-19 yo: 4 722	57 382	n/r	n/r	<10 yo, 10-19 yo, adults	n/r	Alpha	n/a	**Transmission risk OR**[6] **(10-19 yo vs. <10 yo)** OR 0.83 (95%CI[7]: 0.69-0.99) (<10 yo: reference)<br><br>no difference in transmission risk compared to index cases aged 30-70 yo	**vaccination status:** n/r **sequencing:** all index cases sequenced, no information on contacts
Julin et al. [63]	cohort study	Norway	households	3<br><br>alpha: 2<br><br>non-VOC: 1	55<br><br>alpha: 16<br><br>non-VOC: 39	55<br><br>alpha: 16<br><br>non-VOC: 39	68<br><br>alpha: 20<br><br>non-VOC: 48	<20 yo, adults	median: 31 yo (IQR[8] 2-73)	Alpha	n/a	**Transmission risk (irrespective of VOC)** **Viral loads (mean, <20 yo vs. adults)** 2.09 log10 copies/ul RNA[9] vs. 2.98 log10 copies/ul RNA<br><br>**Length of testing positive (mean days, <20 yo vs. adults)** 11.3 (95%CI: 7.6–15.1) vs 16.4 (95%CI: 13.5–19.3), p[10] = 0.03	**vaccination status:** none vaccinated **sequencing:** all sequenced

[6] OR: Odd Ratio
[7] 95%CI: 95% Confidence Interval
[8] IQR: Interquartile Range
[9] RNA: Ribonucleic acid
[10] p: p-value

For peer review only - http://bmjopen.bmj.com/site/about/guidelines.xhtml

Loenenbach et al. [52]	cohort study	Germany	schools<br>kindergartens<br>crèches<br>households	22	16	59	33	≤6 yo, adults	n/r	Alpha	n/a	**Transmission risk**<br>**SAR[11]**<br>**(≤6 yo vs. adults)**<br>39% (95%CI: 28-52) vs. 33% (95%CI: 20-50)<br>RR[12] 1.17 (95%CI: 0.66-2.09)	**vaccination status:** n/r<br>**sequencing:** n/r
Lorthe et al. [53]	cohort study	Switzerland	schools<br>kindergartens<br>crèches<br>households	n/r	n/r	24	2	≤6 yo, adults	n/r	Alpha	n/a	**Transmission risk**<br>**SAR**<br>**(≤6 yo vs. adults)**<br>12.5% (3/24) vs. 50% (1/2)	**vaccination status:** n/r<br>**sequencing:** all cases
Loss et al. [64]	case-control study	Germany	schools<br>kindergartens<br>crèches<br>households	15<br><br>alpha: 9<br><br>non-VOC: 6	9<br><br>alpha: 4<br><br>non-VOC: 5	172<br><br>alpha: 114<br><br>non-VOC: 58	38	≤6 yo, adults	n/r	Alpha	**Transmission risk**<br>**AR[13]**<br>**(alpha vs. non-VOC)**<br>16.7% (19/114) vs. 8.6% (5/58)	**Transmission risk**<br>**AR**<br>**(≤6 yo vs. adults)**<br>16.7% (19/114) vs. 10.5% (4/38)	**vaccination status:** n/r<br>**sequencing:** majority sequenced, rest was assumed based on date of sampling and national prevalence
Buchan et al. [65]	cohort study	Canada	households	715<br><br><10 yo<br>alpha: 55<br>non-VOC: 195<br><br>10-19 yo<br>alpha: 114<br>non-VOC: 351	4 902<br><br>alpha: 1 149<br>non-VOC: 3 753	n/r	n/r	<10 yo, 10-19 yo, adults	n/r	Alpha	**Transmission risk**<br>**SAR**<br>**(alpha vs. non-VOC)**<br>aRR[14] (<10y o): 1.47 (95%CI: 0.81-2.65)<br>aRR (10-19 yo): 1.30 (95%CI: 0.80-2.10)	n/a	**sequencing:** all cases either sequenced or screened positive for N501Y (marker for alpha variant) by PCR[15]

[11] SAR: Secondary Attack Rate
[12] RR: Risk Ratio
[13] AR: Attack Rate
[14] aRR: Adjusted Risk Ratio
[15] PCR: Polymerase Chain Reaction

For peer review only

Waltenburg et al. [17]	cohort study	USA[16]	households	36<br><br>alpha: 21<br><br>non-VOC: 10	n/a	111<br><br>alpha : 61<br><br>non-VOC : 39	n/a	<5 yo<br>5-11 yo<br>12-17 yo	median: 12 yo (IQR 7-15)	Alpha	**Transmission risk**<br>**SAR (alpha vs. non-VOC)**<br>55% (24/44) (95%CI: 39-70) vs. 46% (12/26) (95%CI: 27-67); OR 1.52 (95%CI: 0.51-4.53)<br><br>**SAR – alpha (≤11 yo vs. 12-17 yo)**<br>65% (11/17) (95%CI: 38-68) vs. 48% (13/27) (95%CI: 29-68); OR 1.63 (95%CI: 0.4-6.7)	n/a	**vaccination status:** 97% unvaccinated (147/151), 3% partially vaccinated (4/151) (2 primary cases and 2 secondary cases)<br><br>**sequencing:** majority of primary cases sequenced
Ratman et al. [66]	modeling study	USA	population	n/a	n/a	n/a	n/a	<10 yo, 10-19 yo	n/r	Alpha	**Transmission risk**<br>**R[17] (alpha vs. non-VOC)**<br><10yo: <1 vs. <<1<br>10-19 yo: ~1 vs. <<1	n/a	n/a
Somekh et al. [19]	cohort study	Israel	hospital inpatients hospital outpatients	72 426<br><br>alpha: 50 811<br><br>non-VOC: 21 615<br><br>(overall numbers and thus potential index cases)	n/r	alpha: 313 871<br><br>non-VOC: 156 521	n/r	<10 yo	n/r	Alpha	**Transmission risk**<br>**AR (alpha vs. non-VOC)**<br>15.7% (49257/313871) vs. 7.5% (11770/156521)<br>RR: 2.24 (95%CI, 2.20-2.29), p < 0.001	n/a	**sequencing:** n/r

[16] USA: United States of America
[17] R: reproduction number

For peer review only

Allen et al. [67]	case-control study	United Kingdom	households	6 130<br><br>alpha: n/r<br><br>delta: n/r	17 928	n/r	n/r	<10 yo, 10-19 yo, adults	n/r	Alpha, Delta	n/a	**Trasmission risk aOR[18] (delta vs. alpha/ref. adults)**<br><10 yo: 0.98 (95%CI: 0.85-1.14)<br>10-19 yo: 0.71 (95%CI: 0.64-0.79)<br>(reference value 1.00 for 30-39 yo)	**vaccination status:** reported (but not in subanalysis comparing transmission risk in age groups)<br><br>**sequencing:** all index cases sequenced, nationwide 50% of all cases were sequenced at time of study
Trobajo-Sanmartin et al. [68]	cohort study	Spain	population	n/r	n/a	All contacts of <12 yo : 288<br><br>Alpha : 107<br><br>Delta : 181	n/a	<12yo, 12-17 yo, adults	n/r	Alpha, Delta	**Transmission risk SAR (alpha vs. delta)**<br><12 yo : 33% (35/107) vs. 23% (41/181); aRR 1 vs. 0.58 (95%CI: 0.28-1.23), p=0.155<br><br>higher risk of transmission for 12-39yo		**vaccination status:** none vaccinated<br><br>**sequencing:** none

For peer review only

[18] aOR: Adjusted Odd Ratio

Ng et al. [56]	cohort study	Singapore	households	n/r	n/r	472	18-29 yo : 1 467	≤11 yo, 12-17 yo, adults	median (contacts): 36 yo (IQR 26-51)	Delta	n/a	**Transmission risk** **SAR** **(≤11 yo vs. 18-29 yo)** 25.2% (88/349) vs. 9.4% (138/1457); aOR: 2.37 (95%CI:1.57-3.60), p<0.0001 **SAR** **(12-17 yo vs. 18-29 yo)** 17.9% (22/123) vs. 9.4% (138/1457) ; aOR 1.81 (95%CI: 0.91-3.6), p=0.089	**vaccination status:** >12 yo partially vaccinated (results adjusted for vaccination status) **sequencing:** partially sequenced
Lorthe et al. [59]	cohort study	Switzerland	schools kindergartens crèches, households	n/r	n/a	52	n/a	3-7 yo, adults	n/r	Omicron	**Transmission risk** **SAR** 48% (25/52)	n/a	**vaccination status:** n/r **sequencing:** none

For peer review only

SARS-CoV-2 variants of concern in children and adolescents with COVID-19: a systematic review

Journal:	*BMJ Open*
Manuscript ID	bmjopen-2023-072280.R1
Article Type:	Original research
Date Submitted by the Author:	09-Aug-2023
Complete List of Authors:	Wiedenmann, Margarethe; Swiss Tropical and Public Health Institute; University of Basel<br>Ipekci, Aziz Mert; University of Bern, Institute of Social and Preventive Medicine<br>Araujo-Chaveron, Lucia; EHESP Rennes-Sorbonne Paris Cité; Institut Pasteur<br>Prajapati, Nirmala; Université Paris-Saclay; Institut national de la santé et de la recherche médicale<br>Lam, Yin Ting; University of Bern, Institute of Social and Preventive Medicine<br>Alam, Muhammad Irfanul ; EHESP Rennes-Sorbonne Paris Cité<br>L'Huillier, Arnaud ; HUG, Département de pédiatrie, gynécologie et obstétrique<br>Zhelyazkov, Ivan; The University of Sheffield<br>Heron, Leonie; University of Bern, Institute of Social and Preventive Medicine<br>Low, Nicola; University of Bern, Institute of Social and Preventive Medicine<br>Goutaki, Myrofora ; University of Bern, Institute of Social and Preventive Medicine
**Primary Subject Heading**:	Epidemiology
Secondary Subject Heading:	Infectious diseases, Paediatrics, Public health
Keywords:	COVID-19, Public health < INFECTIOUS DISEASES, Systematic Review

SCHOLARONE™
Manuscripts

I, the Submitting Author has the right to grant and does grant on behalf of all authors of the Work (as defined in the below author licence), an exclusive licence and/or a non-exclusive licence for contributions from authors who are: i) UK Crown employees; ii) where BMJ has agreed a CC-BY licence shall apply, and/or iii) in accordance with the terms applicable for US Federal Government officers or employees acting as part of their official duties; on a worldwide, perpetual, irrevocable, royalty-free basis to BMJ Publishing Group Ltd ("BMJ") its licensees and where the relevant Journal is co-owned by BMJ to the co-owners of the Journal, to publish the Work in this journal and any other BMJ products and to exploit all rights, as set out in our licence.

The Submitting Author accepts and understands that any supply made under these terms is made by BMJ to the Submitting Author unless you are acting as an employee on behalf of your employer or a postgraduate student of an affiliated institution which is paying any applicable article publishing charge ("APC") for Open Access articles. Where the Submitting Author wishes to make the Work available on an Open Access basis (and intends to pay the relevant APC), the terms of reuse of such Open Access shall be governed by a Creative Commons licence – details of these licences and which Creative Commons licence will apply to this Work are set out in our licence referred to above.

Other than as permitted in any relevant BMJ Author's Self Archiving Policies, I confirm this Work has not been accepted for publication elsewhere, is not being considered for publication elsewhere and does not duplicate material already published. I confirm all authors consent to publication of this Work and authorise the granting of this licence.

For peer review only

SARS-CoV-2 variants of concern in children and adolescents with COVID-19: a systematic review

Margarethe Wiedenmann[1,2], Aziz Mert Ipekci[3], Lucia Araujo Chaveron[4,5], Nirmala Prajapati[6,7], Yin Ting Lam[3], Muhammad Irfanul Alam[4], Arnaud G. L'Huillier[8], Ivan Zhelyazkov[9], Leonie Heron[3], Nicola Low[3*], Myrofora Goutaki[3*]

[1] Swiss Tropical and Public Health Institute, Basel, Switzerland
[2] University of Basel, Basel, Switzerland
[3] Institute of Social and Preventive Medicine (ISPM), University of Bern, Bern, Switzerland
[4] EHESP French School of Public Health, Paris/Rennes, France
[5] Institute Pasteur, Paris, France
[6] Université Paris-Saclay, Paris, France
[7] Institut national de la santé et de la recherche médicale (INSERM), Paris, France
[8] Geneva University Hospitals and Faculty of Medicine, Geneva, Switzerland
[9] University of Sheffield, Sheffield, UK

[*]These authors have contributed equally to this work and share last authorship

Correspondence to

Margarethe Wiedenmann

margarethe.wiedenmann@swisstph.ch

For peer review only

For peer review only - http://bmjopen.bmj.com/site/about/guidelines.xhtml

Abstract

Objectives: Infections by SARS-CoV-2 variants of concern (VOCs) might affect children and adolescents differently than earlier viral lineages. We aimed to address five questions about SARS-CoV-2 VOC infections in children and adolescents: i) symptoms and severity, ii) risk factors for severe disease, iii) the risk of infection, iv) the risk of transmission and v) long-term consequences following a VOC infection.

Design: Systematic review.

Data sources: The COVID-19 Open Access Project database was searched up to 1 March 2022 and PubMed was searched up to 9 May 2022.

Eligibility criteria: We included observational studies about Alpha, Beta, Gamma, Delta and Omicron VOCs among ≤18-year-olds. We included studies in English, German, French, Greek, Italian, Spanish and Turkish.

Data extraction and synthesis: Two reviewers extracted and verified the data and assessed the risk of bias. We descriptively synthesised the data and assessed the risks of bias at the outcome level.

Results: We included 53 articles. Most children with any VOC infection presented with mild disease, with more severe disease being described with the Delta or the Gamma VOC. Diabetes and obesity were reported as risk factors for severe disease during the whole pandemic period. The risk of becoming infected with a SARS-CoV-2 VOC seemed to increase with age, while in daycare settings the risk of onward transmission of VOCs was higher for younger than older children or partially vaccinated adults. Long-term symptoms following an infection with a VOC were described in <5% of children and adolescents.

Conclusion: Overall patterns of SARS-CoV-2 VOC infections in children and adolescents are similar to those of earlier lineages. Comparisons between different pandemic periods, countries and age groups should be improved with complete

For peer review only

For peer review only - http://bmjopen.bmj.com/site/about/guidelines.xhtml

reporting of relevant contextual factors, including VOCs, vaccination status of study participants and the risk of exposure of the population to SARS-CoV-2.

PROSPERO registration number: CRD42022295207.

Strengths and limitations of this study

- This review is based on a wide range of worldwide studies found during repeated systematic searches of different databases, reducing our risk of having missed relevant information.

- We included studies in the various languages the review team spoke, only excluding three studies for language reasons.

- We assessed the included studies' risk of bias at the outcome level to evaluate the quality of the studies included.

- The included studies did not provide information on the specific Omicron subvariants, limiting the interpretation of the respective data.

For peer review only

For peer review only - http://bmjopen.bmj.com/site/about/guidelines.xhtml

Introduction

The role of children and adolescents in the pandemic of severe acute respiratory syndrome coronavirus 2 (SARS-CoV-2), the cause of coronavirus disease 2019 (COVID-19), remains an important topic of discussion. In our systematic review, covering the period when SARS-CoV-2 ancestral strains circulated, we found that children (≤12 years of age) and adolescents (13-17 years of age) usually present with mild disease, with a minority of children of all ages (<18 years) remaining asymptomatic throughout the course of infection, and their risk of acquiring and transmitting SARS-CoV-2 seemingly increases with age.[1]

Since late 2020 SARS-CoV-2 variants of concern (VOCs) have emerged, with the Alpha variant (previously referred to as B.1.1.7) being the first. These lineages possess one or more mutations, which are associated with different phenotypic properties, such as changes in the severity of disease, increased transmissibility, or the ability to evade infection- or vaccine-induced immunity.[2] The circulation of Alpha, Beta, Gamma, Delta variants and the Omicron group of variants and subvariants has led to large waves of COVID-19 in different countries.[3] At around the same time as the first VOCs emerged and spread, vaccines that gave protection against infection and severe disease caused by the original SARS-CoV-2 lineage were introduced in adults.[4,5] Vaccines were first licensed for 16-18 year-olds in spring 2021 and later for children <16 years old, but vaccination availability and uptake varied by country.[6] Also, preventive interventions introduced at the population level have been relaxed in many countries. A re-evaluation of the role of children and adolescents in the SARS-CoV-2 pandemic is warranted, given the potential for the mutations in VOCs to affect age-associated patterns of COVID-19 clinical disease and SARS-CoV-2 transmissibility, and the higher numbers of children and adolescents testing positive for SARS-CoV-2 than in the pre-VOC period.

The objectives of this study were to address five research topics about SARS-CoV-2 VOC infections in children and adolescents younger than 20 years: 1. What are the symptoms caused by SARS-CoV-2 VOCs in children and adolescents, and do they differ from those of SARS-CoV-2 wild type infections? 2. What are the risk factors for severe COVID-19 in children and adolescents infected with a SARS-CoV-2 VOC? 3. Does the risk of infection with a SARS-CoV-2 VOC differ between children,

For peer review only - http://bmjopen.bmj.com/site/about/guidelines.xhtml

adolescents and adults who have not received a COVID-19 vaccine? 4. Does the risk of transmission of a SARS-CoV-2 VOC differ between children, adolescents and adults who have not received a COVID-19 vaccine? 5. Does the risk of developing long-term effects (duration >28 days) after infection with a SARS-CoV-2 VOC differ between children, adolescents and adults who have not received a COVID-19 vaccine?

Methods

Design

We registered a protocol for this review in the International Prospective Register of Systematic Reviews (PROSPERO) (registration ID: CRD42022295207).[7] We report the study according to the Preferred Reporting Items for Systematic Review and Meta-Analyses guidelines (Supplementary Table 1).[8]

Search strategy

We searched: 1) the COVID-19 Open Access Project (COAP) database, which aggregates COVID-19 research from PubMed, EMBASE, PsycINFO, bioRxiv and medRxiv from 1 January 2020 to 1 March 2022 and 2) PubMed using MeSH terms from 1 August 2020 to 7 March 2022. [9] Owing to large numbers of records that did not fulfil the inclusion criteria, we revised the PubMed search strategy, including each VOC, identified by their Greek letter and Pangolin lineage terminology, and conducted a new search between 8 March and 9 May 2022. We also searched reference lists of systematic reviews and included studies, and we included studies suggested by experts in the field, but not identified by our search. Full search terms are included in the Supplementary Note 1.

Eligibility and screening

Observational epidemiological studies (case series with more than one case, cross-sectional studies, case-control studies and cohort studies) and modelling studies that were based on analysis of the original data were eligible for inclusion if data collection occurred during a period when VOCs were predominant in the study setting. The presence of a VOC could be based on genetic sequencing in the study or by assumptions based on the authors' reporting, the dominant lineage at the time of testing, and the study location. We did not apply any geographical or language

For peer review only - http://bmjopen.bmj.com/site/about/guidelines.xhtml

For peer review only

restrictions to the search, but we only read the full text of papers written in languages spoken fluently by the review team (English, German, French, Greek, Italian, Spanish or Turkish). Identified titles and abstracts were screened for potential eligibility by one member from an international team of ten reviewers, including records about which they were unsure. A second member verified the selection. If either reviewer was unsure about the potential eligibility, we obtained the full-text. We followed the same approach for the full-text assessment. A third reviewer resolved disagreements.

Data extraction

We developed, piloted and revised a data extraction template in Microsoft Excel at the beginning of the study. One reviewer extracted data from each included study and a second reviewer verified the extraction. Extracted data included information on the publication characteristics, study design, study setting, study population overall and those younger than 20 years old, age groups, prevalent VOC(s) at the time of study, and outcomes for each relevant review question.

Risk of bias assessment

We assessed the risk of bias at the outcome level using published tools for cohort and case control studies.[10,11] The credibility and relevance of modelling studies was evaluated using a published checklist.[12] To help with interpretation of the findings, we also recorded whether the authors of included studies reported on the stage of the SARS-CoV-2 pandemic, on any existing control measures, or on the vaccination status of the study participants at the time of the study.

Data synthesis

We synthesised the information from the included studies descriptively for each question. We tabulated summaries of the data extracted, including the number of studies identified for each VOC, the age groups studied and the outcomes reported.

Definitions of disease severity differed between studies. To allow comparisons, we applied the following categorisation: mild or asymptomatic disease (patients were not hospitalised), moderate disease (patients were hospitalised, but did not require intensive care), severe disease (patients were hospitalised and intensive care was necessary). If we did not have sufficient information to apply these categories, we used the authors' definitions and reported these in the text. Where possible, we

For peer review only - http://bmjopen.bmj.com/site/about/guidelines.xhtml

presented the results separately for different age groups: neonates and infants 0-1 years old; young children 1-4 years old; older children 5-9 years old; younger and older adolescents 10-20 years old. If the intended age groups were not reported, we used the groups reported by study authors. We categorised the country in which the study was conducted according to World Bank income groups as a simplified proxy for the level of government resources spent on health services and public health surveillance activities.[13] We report our findings descriptively. There were too few studies that reported quantitative findings in a comparable way to conduct meta-analysis. Any comparison with non-VOC lineages (any strain not considered by the World Health Organization as a variant of concern) included in this manuscript were made by the authors of the original manuscripts.[2] We did not make independent comparisons between outcomes during COV and non-VOC periods.

Patient and public involvement

None.

Results

We screened 2407 articles published up to 09 May 2022, 53 of which were included (Figure 1 and Supplementary Figures 1-4). According to World Bank data, forty-seven (89%) articles were from 14 high-income countries, six (11%) were from five upper middle-income countries and none was from a lower middle-income or low-income country (Table 1 and Figure 2).

Of the 53 studies included in the review, 44 (83%) were cohort studies, 4 (7.5%) were cross-sectional studies, 3 (5.7%) were case-control studies, 1 (1.9%) was a modelling study and 1 (1.9%) was a case-series (Table 1). Some included studies compared different variants and others different age groups, infected with the same variant.

Most studies were judged to be at moderate risk of bias in most domains. There were high risks of bias in a few domains especially regarding matching of exposed and unexposed cohorts as well as the assessment of prognostic factors (irrespective of the research question they addressed) as most studies did not control for possible confounding factors (Supplementary Tables 2-6).

For peer review only - http://bmjopen.bmj.com/site/about/guidelines.xhtml

Table 1. Characteristics of included studies

**Characteristic**	**Total**	**Variant of concern**				
		**Alpha**	**Beta**	**Gamma**	**Delta**	**Omicron**
Number of studies, n*	53	27	2	2	27	12
Review question addressed, n*						
- review question no. 1	35	13	2	2	21	11
- review question no. 2	2	1	1	1	2	2
- review question no. 3	15	10	-	-	4	1
- review question no. 4	15	13	-	-	3	1
- review question no. 5	-	2	1	1	2	-
Study design						
- cohort	44	21	2	2	24	9
- case-control	3	3	-	-	-	-
- cross-sectional	4	2	-	-	3	2
- modelling	1	1	-	-	-	-
- case series	1	-	-	-	-	1
Income category according to world bank categories, n						
- high income	47	26	1	1	23	11
- upper middle income	6	1	1	1	4	1
- lower middle income	-	-	-	-	-	-
- low income	-	-	-	-	-	-

* The total number of studies included is 53. The added number for each VOC is more than 53 as some studies reported on more than just one VOC.

Review question 1: Symptoms and severity of disease among children and adolescents infected with VOCs

Symptoms

Symptoms experienced by children and adolescents infected with a VOC were presented in 19 studies involving the Alpha (n=7), Delta (n=6), Omicron (n=4) or Gamma variant (n=1) (Supplementary Tables 7-10).[14-32] Children and adolescents experienced a wide range of symptoms. We did not identify any symptoms consistently associated with a specific VOC or whether symptom frequencies with VOC infections differed from the pre-VOC period. One study reported on croup in children and adolescents infected with the Omicron variant and found an increase in croup cases in hospital inpatients and outpatients compared with the pre-omicron period (median weekly croup cases (Omicron vs. pre-Omicron): 11 [IQR 2–17] vs. 0 (IQR 0-0), $p < 0.001$) (Supplementary Table 9).[14] We did not find any studies investigating symptoms in children infected with the Beta variant. A

For peer review only - http://bmjopen.bmj.com/site/about/guidelines.xhtml

detailed comparison between studies was not possible owing to differences in study populations (community or health care setting), symptoms recorded and age groups reported.[15-32]

Severity

Children and adolescents mostly experienced mild disease when infected with a SARS-CoV-2 VOC. More severe infections were described with the Gamma variant compared to pre-VOC variants. On the other hand, infections with the Omicron variant were less severe compared to Delta variant infections.

Paediatric disease severity associated with SARS-CoV-2 VOC infections was described in 25 studies (Supplementary Tables 7-10).[18-20,26-29,31-48] The Alpha variant did not appear to result in more severe COVID-19 disease than disease caused by non-VOCs in children and adolescents in five studies.[18-20,33,34] In two studies reporting on paediatric inpatients and outpatients one from Brazil and one from the USA, the overall hospitalisation rate as well as the ICU admission rate of children infected with the Gamma variant was higher than for non-VOCs (Gamma vs. non-VOC: 28.3% vs. 24.9% [35], Gamma vs. non-VOC: 5.2% vs. 1.2% [36]).[35,36] Severity of Delta variant infection was compared with the pre-Delta period (including periods where previous VOCs where predominant) in eight studies but the reported data were inconsistent, and differences in disease severity could not be assessed.[26-28,37-41] Studies of Omicron variant infection described less severe disease than infection with the Delta variant in children and adolescents <18 years old, based on data from nine studies from South Africa, Qatar and the USA.[29,31,32,42-47]

One study from Denmark analysed the symptoms and severity of disease experienced by children and adolescents younger than 17 years old, hospitalised for multi-inflammatory syndrome in children (MIS-C) during the Delta and non-VOC predominant periods. The range of symptoms was similar in both periods, as was the proportion of children admitted to ICU (Delta, 55% (28/51) vs. non-VOC 52% (12/23)).[48]

We did not find any studies showing an association between the SARS-CoV-2 Omicron lineages and the global outbreak of paediatric hepatitis of unknown origin, which has been attributed to adenoviruses in spring 2022.[49]

For peer review only - http://bmjopen.bmj.com/site/about/guidelines.xhtml

No studies reported on how symptoms or severity differed between different paediatric age groups or compared children and adolescents infected with a VOC to adults infected with the same variant.

Review question 2: Risk factors for severe COVID-19 in children and adolescents infected with a SARS-CoV-2 VOC

We found one article that used data from the National COVID-19 database in Qatar and compared risk factors for severe COVID-19 in children and adolescents infected with the Omicron and Delta variants. The study showed that children and adolescents <18 years old with ≥1 comorbidity (mostly chronic lung disease) were more likely to have moderate or severe disease than those without comorbidities if they were infected with the Omicron lineage (aOR 95%CI 3.2, 1.1-9.0), but not if they were infected with the Delta lineage (aOR 95%CI 0.8, 0.5-1.4).[31] One paper reported on risk factors for severe COVID-19 disease in children 5-11 years old during the whole pandemic period, including the time when the Omicron variant became predominant. The study assessed several possible risk factors and found evidence of an increased risk of severe disease in children with diabetes or obesity.[46]

Review question 3: Risk of becoming infected with a SARS-CoV-2 VOC

The risk of becoming infected with a SARS-CoV-2 VOC was analysed in time periods when the Alpha, Delta or Omicron variants were predominant in 15 studies (Supplementary Table 11).[15,17-19,33,50-59] Immunity due to previous infection or vaccination appeared to be a relevant factor in determining the risk of infection but these factors were poorly reported in the included studies. Some studies (4/15) summarised the vaccination status of the entire study sample, which ranged from an undefined proportion of partially vaccinated individuals to 70% fully vaccinated, without analysing it at the individual level for adults and children and adolescents.[18,50-52]

Six studies evaluated data from Alpha variant outbreaks in daycare centres and compared the risk of infection between children and adults.[15,19,53-56] They generally showed a lower attack rate for infection with the Alpha variant ranging from 0% to 27% for young children (mostly ≤6 years old) than for adults (0%-71%). However, there were large variations in attack rates between different facilities or

For peer review only - http://bmjopen.bmj.com/site/about/guidelines.xhtml

classes at the same facility. The study participants' vaccination status was not reported in any of these studies.

One study reported the attack rates of children by age group. The attack rates for young children (≤4 years old) decreased with increasing age from 44% to 4%, with the highest rate for infants <1 year old and the lowest for children 3-4 years old.[15] The attack rates of older children (10-19 years old) and adults (30-39 years old) were similar in one study from Qatar, but the vaccination status was not reported.[57]

In one study analysing household transmission of the Alpha variant in the USA, the risk of infection was high in both younger children ≤11 years old (78%, 95%CI: 51-76%) and older children and adolescents 12-17 years old (56%, 95%CI: 40-72%) with overlapping CIs.[18] Compared with the pre-VOC period, a similar or lower risk of infection was reported in three household or school studies for children and adolescents of all age groups when the Alpha variant was predominant.[18,19,53] However, in two studies from Israel, including a large hospital study, a higher risk of infection for the Alpha variant than for non-VOCs was described for all paediatric age groups.[17,33] Data on the risk of infection during the Delta predominant time period analysed in four studies comparing children and adolescents to adults were inconclusive.[50-52,58] Two of these four studies, one cohort study from Singapore and one from the UK found the risk of infection to be higher for younger children ≤11years old/school-aged children 5-18 years old than for adults who were either completely or partially vaccinated.[51,52] One study analysed the risk of infection in daycare centres during the Omicron predominant time period and found it to be slightly lower for young children 3-7 years old (44%) than for adults (50%). As described, during the Alpha-predominant period large variations in attack rates (15.4%-61.1%) were observed between different school classes.[59]

Review question 4: Risk of SARS-CoV-2 transmission from children and adolescent infected with a VOC

The risk of SARS-CoV-2 transmission was evaluated in time periods when Alpha, Delta or Omicron variants were predominant in 14 studies (Supplementary Table 12).[18,33,50,55,59,60-68]

Overall, transmission risk with a VOC was reported to be higher for younger children in most studies. A higher risk of onward SARS-CoV-2 Alpha variant transmission in

For peer review only - http://bmjopen.bmj.com/site/about/guidelines.xhtml

household settings from younger children <10 years old than from older children 10-19 years old and adults was found in four studies.[55,60-62] These studies did not report on whether the participating adults or older children were vaccinated. One household study from the USA found comparable transmission of Alpha variant infections from children ≤11 years old compared with adolescents 12-17 years old (97% of whom were unvaccinated) (OR 1.37, 95% CI 0.61, 3.04).[18] The risk of transmission from children <10 years old with an Alpha variant infection was found to be higher than from children with a pre-VOC or non-VOC infection in three studies.[33,62,63] One study found the transmission risk from 10-19 year olds with an Alpha variant infection to be similar to that of pre-VOC infection.[63] Comparing the risk of onward transmission between age groups for participants infected with either the Alpha or Delta variant, the risk was similar for younger children <12 years old and lower for older children and adolescent 10-19 years old, compared with 30-39 year old adults.[64,65] One household transmission study from Singapore found the transmission risk of younger children ≤11 years old infected with the Delta variant to be higher (25%) than the transmission risk of young adults 18-29 years old who were at least partially vaccinated (9%).[50] The availability of evidence on the risk of onward transmission of children with an Omicron variant infection was limited. We only found one small study that analysed the risk of onward transmission of young children ≤6 years old with an Omicron variant infection in a daycare setting and found it to be lower (3/24, 12.5%) than the risk of onward transmission from adults (1/2, 50%).[59]

Review question 5: Risk of long-term symptoms or signs (duration >28 days) following an infection with a SARS-CoV-2 VOC

Few children reported long-term symptoms or signs of infection with a defined duration of >28 days.[26,27] The risk for children and adolescents experiencing symptoms longer than 28 days was similar for children infected with the Alpha (12/694, 1.73%) and the Delta lineage (15/706, 2.12%) in the UK, and was similar for younger (5-11 years old) compared to older (12-17 years old) children infected with the same variant. For those children with longer illness duration, no new symptoms developed after day 28.[26] A study comparing Danish children who tested positive for SARS-CoV-2 with a reference cohort of children who tested negative during the overall pandemic period (Alpha and Delta variant predominant times included) found

For peer review only - http://bmjopen.bmj.com/site/about/guidelines.xhtml

that 0.12% (95%CI: 0.09%-0.15%) of children experienced symptoms for more than 29 days (58 of 48948 children).[27]

Discussion

Children and adolescents (<20 years old) with a SARS-CoV-2 VOC infection mostly experienced mild disease and the symptoms experienced did not vary widely between VOCs. Compared to non-VOC and other VOC induced infections more severe infection was observed with either the Delta or the Gamma SARS-CoV-2 variant. Compared to otherwise healthy peers, children and adolescents with comorbidities appeared to have an increased risk of infection by Omicron, but not Delta, and diabetes and obesity were reported as risk factors for severe infection during the whole pandemic period. The studies reviewed suggest that the risk of SARS-CoV-2 infection in daycare centres was lower overall for younger children than for adults, while the risk compared to adults decreased with increasing age of the children. The transmission risk of SARS-CoV-2 from younger children infected with a VOC seemed to have been higher than that of older children and adults as well. The proportion of children and adolescents reporting symptoms more than 28 days after a SARS-CoV-2 VOC infection was low. Interpretation and generalisation of the evidence was limited by inconsistent reporting of age groups and by inadequate reporting of vaccination and other control measures in included studies.

Strength and limitations

We used a systematic approach to search different databases, covering the period of VOC circulation that includes the first Omicron variants (BA.1 and BA.2) in 2022. Unlike preceding VOCs, Omicron comprises an increasingly large groups of multiple variants and subvariants. Information on the specific subvariants was not provided in the included studies, which limits the interpretation of the associated data in our review. We included a broad selection of study designs in various languages and screened reference lists of included studies. Our search strategy might have missed relevant studies, although we only excluded three records for language reasons. A limitation of the review is that misclassification of the variant could have occurred as only a minority of study samples from all cases was sequenced in several studies and we relied on dates during which a variant was dominant. Non-differential

For peer review only - http://bmjopen.bmj.com/site/about/guidelines.xhtml

misclassification would reduce the magnitude of associations with VOC infection status.

Interpretation and implications of findings

The severity of SARS-CoV-2 infections, the risk of becoming infected and the risk of onward transmission depend on both intrinsic factors associated with the person (e.g. host immunity) and the pathogen (e.g. variant/subvariant transmissibility, virulence, immune evasion) and extrinsic factors (e.g. study setting, COVID-19 at the population level, vaccination status of study participants, preventive measures in place). Differentiation between the contributions of intrinsic and extrinsic factors in studies included in this review was challenging because frequent deficiencies in reporting of extrinsic factors hindered comparisons and interpretation of outcomes from different regions or different pandemic periods, even from the same country. All studies were conducted in high or upper-middle income countries, which might affect the generalisability of the review findings to children in settings with less-resourced health systems, lower vaccine coverage and different patterns of comorbidity and co-infection with other respiratory viruses. We assessed the risk of bias using published tools for cohort and case-control studies but found that the selection bias was hard to assess, especially for studies addressing the symptoms and severity of disease in hospitalised populations, since reporting of indications for hospitalisation (especially for children) varied between countries and between different stages of the pandemic.[10,11]

Risk of exposure to, and transmission of, SARS-CoV-2

The risk of exposure to SARS-CoV-2 has a direct effect on the risk of becoming infected, which influences the risk of onward transmission. Younger children are more dependent on adult care takers, who were most likely to be the index case, than older children and adolescents. This increased risk of exposure to infected adults might explain their higher risk of becoming infected and for onward transmission compared to older children and adolescents. The risk of exposure to SARS-CoV-2 also influences the interpretation of the data on disease severity in children and adolescents with chronic diseases. We found an increased risk of

For peer review only - http://bmjopen.bmj.com/site/about/guidelines.xhtml

severe disease for children with diabetes or obesity, but not for children and adolescents with respiratory conditions, except if they were infected with Omicron.[46] As high-risk populations have been known to take additional measures to stay safe since the beginning of the pandemic, these findings could be partly explained by more intense shielding.[69]

Vaccination status

Vaccines protecting people from severe COVID-19 were first licensed for adults at the end of 2020 at around the same time as the Alpha VOC became widespread. These vaccines also reduced the risk of becoming infected with SARS-COV-2 as well as the risk of transmitting it.[4,70,71] At more recent stages of the pandemic, such as when the Omicron lineage first emerged, the original COVID-19 vaccine from Pfizer (Comirnaty) had become licensed for children ≥5 years old and offered protection from severe disease and at least partial protection from becoming infected and potentially from onward transmission.[72-74] Vaccine uptake, however, remained low for children in most countries. More importantly, a majority of countries with less financial resources and restricted access to health services have not received a sufficient number of vaccine doses to ensure high vaccination coverage even for healthcare workers and vulnerable elderly populations let alone for children and adolescents.[75] Irrespective of the prevalent SARS-CoV-2 lineage this low vaccine uptake and accessibility increased the risk of children and adolescents not only becoming infected themselves but of transmitting the disease to other children and adults. Vaccines for children <5 years old have only recently been licensed in countries with sufficient financial resources.[76,77] In addition to the increased care needs of younger children and thus their higher risk of potential exposure to COVID-19 infected adults, their higher risk of infection and onward transmission compared to older children and adolescents might also be explained by a difference in vaccination status.

Recommendations for hospitalisation

Recommendations for hospitalisation (as a sign of at least moderate disease) and treatment guidelines for children and adolescents infected with COVID-19 were not standardised, either at the country level or internationally, have changed over the course of the pandemic and might differ between different hospitals within and

For peer review only - http://bmjopen.bmj.com/site/about/guidelines.xhtml

between countries. In addition, the availability of beds on wards and ICUs varied between countries and over the course of the pandemic greatly influencing the decisions made on hospitalisations. These variations made it impossible to compare the prevalence of moderate and severe COVID-19 disease both between countries or between different pandemic periods in the same country, irrespective of the prevalent SARS-CoV-2 lineage.

Conclusion

Children are susceptible to infection with a SARS-CoV-2 VOC and can transmit them. Overall patterns of SARS-CoV-2 caused by VOCs among children and adolescents appear similar to those observed in the pre-VOC period. Identification of characteristics associated with specific VOCs or VOC infections in certain age groups is challenged by the frequent lack of data reporting of contextual factors, including the vaccination status of included participants and levels of prevalent SARS-CoV-2. Further studies assessing age-related differences in the risk of infection, the risk of transmission as well as risk factors for severe disease and long-term symptoms or signs after SARS-CoV-2 VOC infection are needed globally. These studies should report the vaccination status of participating adults and children as well as any measures affecting transmission (exposure type and intensity) and hospitalisation (local guidelines) during the study period. The available data are from studies in higher income settings, where the population is not only assumed to have easier access to health services but where resources for surveillance and research are also more readily available than in low- and middle-income settings. Owing to a lack of access to vaccines and generally low vaccine coverage, children account for a largely unvaccinated part of the population, especially in low- and middle-income countries. Local data acquisition and dissemination about SARS-CoV-2 in children and adolescents remain important both for affected countries and worldwide as SARS-CoV-2 continues to mutate and spread.

For peer review only - http://bmjopen.bmj.com/site/about/guidelines.xhtml

Acknowledgements

We would like to thank all members of WHO's Maternal, Newborn, Child and Adolescent Health and Ageing department, who contributed to the development of this review: Kathleen Strong, Theresa Diaz, Anshu Banerjee, Maria van Kerkhove, Abdi Mahamud, Valentina Baltag, Judith Ann Mandelbaum-Schmid.

Contributors

MW, NL and MG conceived and designed the review. MW, LH and MG performed the literature search. MW, MI, LAC, NP, YTL, MIA, ALH, IZ, LH and MG screened the articles for relevance and extracted the data. MW, MG and NL adjudicated as third reviewers in case of disagreements during the screening and extraction. MW, MI, LAC and MG synthesised the data. MW, MI, LAC, LH, NP, YTL, MIA, ALH, NL and MG all discussed the interpretation of the findings. MW and MG wrote the first draft of the manuscript. MI, LAC, NP, YTL, MIA, ALH, LH and NL all reviewed and revised the manuscript and approved the final version.

Competing interests

MW, MI, LAC, NP, YTL, MIA, ALH, IZ, LH, MG and NL declare no conflict of interest.

Funding

MW received funding from the World Health Organization (purchase order: 202807927, award grand number: n/a). MIA received the Erasmus Mundus Excellence Scholarship for a European Master of Public Health (2018-2020, award grand number: n/a). He also received subsistence allowance from WHO HQ during an internship and is now receiving a salary from WHO Bangladesh. NL received funds from the Swiss National Science Foundation (project number 176233) and European Union Horizon 2020 research and innovation programme - project EpiPose (grant agreement number 101003688). MG received grants from the Swiss National Science Foundation Ambizione fellowship (PZ00P3_185923) as well as from the Fondation Johanna Dürmüller-Bol (award grand number: n/a).

Data availability statement

No novel datasets were generated. No additional data are available.

Ethics approval statement

This study did not directly involve human participants or animal subjects and was based on the available literature. Ethics approval by an ethics committee or institutional reviewer board was therefore not necessary.

For peer review only - http://bmjopen.bmj.com/site/about/guidelines.xhtml

References

1. Wiedenmann M, Goutaki M, Keiser O, Stringhini S, Tanner M, Low N. The role of children and adolescents in the SARS-CoV-2 pandemic: a rapid review. Swiss Med Wkly. 2021;151:w30058.

2. World Health Organization. Tracking SARS-CoV-2 variants 2022 [updated May 25 2022. Available from: https://www.who.int/activities/tracking-SARS-CoV-2-variants.

3. Thakur V, Bhola S, Thakur P, Patel SKS, Kulshrestha S, Ratho RK, et al. Waves and variants of SARS-CoV-2: understanding the causes and effect of the COVID-19 catastrophe. Infection. 2022;50(2):309-25.

4. Ssentongo P, Ssentongo AE, Voleti N, Groff D, Sun A, Ba DM, et al. SARS-CoV-2 vaccine effectiveness against infection, symptomatic and severe COVID-19: a systematic review and meta-analysis. BMC Infect Dis. 2022;22(1):439. doi: 10.1186/s12879-022-07418-y.

5. Lauring AS, Tenforde MW, Chappell JD, Gaglani M, Ginde AA, McNeal T, et al. Clinical severity of, and effectiveness of mRNA vaccines against, covid-19 from omicron, delta, and alpha SARS-CoV-2 variants in the United States: prospective observational study. BMJ. 2022;376:e069761. doi: 10.1136/bmj-2021-069761.

6. Hunter DJ, Abdool Karim SS, Baden LR, Farrar JJ, Hamel MB, Longo DL, et al. Addressing Vaccine Inequity - Covid-19 Vaccines as a Global Public Good. N Engl J Med. 2022;386(12):1176-9.

7. Wiedenmann M, Goutaki M, Low N, Stringhini S, l'Huillier A. The role of children/adolescents in the SARS-CoV-2 pandemic with regards to variants of concern: a rapid review PROSPERO 2022 CRD42022295207 2022 [Available from: https://www.crd.york.ac.uk/prospero/display_record.php?RecordID=295207.

8. PRISMA, Transparent Reporting of Systematic Reviews and Meta-Analyses. PRISMA Checklist 2021 [Available from: https://prisma-statement.org/PRISMAStatement/Checklist.]

9. COVID-19 Open Access Project. (2021). *Living Evidence on COVID-19*. Retrieved from https://ispmbern.github.io/covid-19/living-review/

10. CLARITY Group at McMaster University. Tool to Assess Risk of Bias in Cohort Studies [Available from: https://www.evidencepartners.com/wp-content/uploads/2021/03/Tool-to-Assess-Risk-of-Bias-in-Cohort-Studies-DistillerSR.pdf.

11. CLARITY Group at McMaster University. Tool to Assess Risk of Bias in Case Control Studies [Available from: https://www.evidencepartners.com/wp-content/uploads/2021/03/Tool-to-Assess-Risk-of-Bias-in-Case-Control-Studies-DistillerSR.pdf.

12. Jaime Caro J, Eddy DM, Kan H, Kaltz C, Patel B, Eldessouki R, et al. Questionnaire to assess relevance and credibility of modeling studies for informing health care decision making: an ISPOR-AMCP-NPC Good Practice Task Force report. Value Health. 2014;17(2):174-82. doi: 10.1016/j.jval.2014.01.003.

For peer review only - http://bmjopen.bmj.com/site/about/guidelines.xhtml

13. The World Bank. Data for High income, Low income, Lower middle income, Upper middle income [Available from: https://data.worldbank.org/?locations=XD-XM-XN-XT.

14. Brewster RC, Parsons C, Laird-Gion J, Hilker S, Irwin M, Sommerschield A, et al. COVID-19-Associated Croup in Children. Pediatrics. 2022;149(6).

15. Roberts RJ, Brooker A, Lakshman R. Outbreak of SARS-CoV-2 in a Children's Nursery in the United Kingdom. Pediatr Infect Dis J. 2021;40(12):e455-e8. doi: 10.1097/INF.0000000000003362.

16. Ulyte A, Radtke T, Abela IA, Haile SR, Ammann P, Berger C, et al. Evolution of SARS-CoV-2 seroprevalence and clusters in school children from June 2020 to April 2021: prospective cohort study Ciao Corona. Swiss Med Wkly. 2021;151:w30092. doi: 10.4414/smw.2021.w30092.

17. Somekh I, Sharabi A, Dory Y, Simoes EAF, Somekh E. Intrafamilial Spread and Altered Symptomatology of SARS-CoV-2, During Predominant Circulation of Lineage B.1.1.7 Variant in Israel. Pediatr Infect Dis J. 2021;40(8):e310-e1. doi: 10.1097/INF.0000000000003167.

18. Waltenburg MA, Whaley MJ, Chancey RJ, Donnelly MAP, Chuey MR, Soto R, et al. Household Transmission and Symptomology of Severe Acute Respiratory Syndrome Coronavirus 2 Alpha Variant Among Children-California and Colorado, 2021. J Pediatr. 2022.

19. Nakel J, Robitaille A, Gunther T, Rosenau L, Czech-Sioli M, Plenge-Bonig A, et al. Comparing susceptibility and contagiousness in concurrent outbreaks with a non-VOC and the VOC SARS-CoV-2 variant B.1.1.7 in daycare centers in Hamburg, Germany. Int J Hyg Environ Health. 2022;240:113928. doi: 10.1016/j.ijheh.2022.113928.

20. Swann OV, Pollock L, Holden KA, Munro APS, Bennett A, Williams TC, et al. Comparison of UK paediatric SARS-CoV-2 admissions across the first and second pandemic waves. Pediatr Res. 2022. doi: 10.1038/s41390-022-02052-5.

21. Stewart DJ, Mudalige NL, Johnson M, Shroff R, du Pre P, Stojanovic J. Acute kidney injury in paediatric inflammatory multisystem syndrome temporally associated with SARS-CoV-2 (PIMS-TS) is not associated with progression to chronic kidney disease. Arch Dis Child. 2022;107(3):e21. doi: 10.1136/archdischild-2021-322866.

22. Li H, Lin H, Chen X, Li H, Li H, Lin S, et al. Unvaccinated Children Are an Important Link in the Transmission of SARS-CoV-2 Delta Variant (B1.617.2): Comparative Clinical Evidence From a Recent Community Surge. Front Cell Infect Microbiol. 2022;12:814782. doi: 10.3389/fcimb.2022.814782.

23. Hao J, Hu XC, Fan MX, Chen J, Cheng QR, Li Z, et al. Analysis of clinical characteristics of 66 pediatric patients with B.1.617.2 (Delta) variant of COVID-19. World J Pediatr. 2022;18(5):343-9.

24. Ryu BH, Hong SI, Lim SJ, Cho Y, Hong KW, Bae IG, et al. Features of COVID-19 Among Children and Adolescents Without Risk Factors Before and After the Delta Variant Outbreak in South Korea. Pediatr Infect Dis J. 2022;41(1):e34-e5. doi: 10.1097/INF.0000000000003394.

For peer review only - http://bmjopen.bmj.com/site/about/guidelines.xhtml

25. Butt AA, Dargham SR, Chemaitelly H, Al Khal A, Tang P, Hasan MR, et al. Severity of Illness in Persons Infected With the SARS-CoV-2 Delta Variant vs Beta Variant in Qatar. JAMA Intern Med. 2022;182(2):197-205. doi: 10.1001/jamainternmed.2021.7949.

26. Molteni E, Sudre CH, Canas LS, Bhopal SS, Hughes RC, Chen L, et al. Illness characteristics of COVID-19 in children infected with the SARS-CoV-2 Delta variant. MedRxiv. 2021. doi: 10.1101/2021.10.06.21264467.

27. Kildegaard H, Lund LC, Hojlund M, Stensballe LG, Pottegard A. Risk of adverse events after covid-19 in Danish children and adolescents and effectiveness of BNT162b2 in adolescents: cohort study. BMJ. 2022;377:e068898.

28. Edward PR, Lorenzo-Redondo R, Reyna ME, Simons LM, Hultquist JF, Patel AB, et al. Severity of Illness Caused by Severe Acute Respiratory Syndrome Coronavirus 2 Variants of Concern in Children: A Single-Center Retrospective Cohort Study. medRxiv. 2021. doi: 10.1101/2021.10.23.21265402.

29. Cloete J, Kruger A, Masha M, du Plessis NM, Mawela D, Tshukudu M, et al. Paediatric hospitalisations due to COVID-19 during the first SARS-CoV-2 omicron (B.1.1.529) variant wave in South Africa: a multicentre observational study. Lancet Child Adolesc Health. 2022. doi: 10.1016/S2352-4642(22)00027-X.

30. Ludvigsson JF. Convulsions in children with COVID-19 during the Omicron wave. Acta Paediatr. 2022. doi: 10.1111/apa.16276.

31. Butt AA, Dargham SR, Loka S, Shaik RM, Chemaitelly H, Tang P, et al. COVID-19 Disease Severity in Children Infected with the Omicron Variant. Clin Infect Dis. 2022.

32. Fowlkes AL, Yoon SK, Lutrick K, Gwynn L, Burns J, Grant L, et al. Effectiveness of 2-Dose BNT162b2 (Pfizer BioNTech) mRNA Vaccine in Preventing SARS-CoV-2 Infection Among Children Aged 5-11 Years and Adolescents Aged 12-15 Years - PROTECT Cohort, July 2021-February 2022. MMWR Morb Mortal Wkly Rep. 2022;71(11):422-8.

33. Somekh I, Stein M, Karakis I, Simoes EAF, Somekh E. Characteristics of SARS-CoV-2 Infections in Israeli Children During the Circulation of Different SARS-CoV-2 Variants. JAMA Netw Open. 2021;4(9):e2124343. doi: 10.1001/jamanetworkopen.2021.24343.

34. Brookman S, Cook J, Zucherman M, Broughton S, Harman K, Gupta A. Effect of the new SARS-CoV-2 variant B.1.1.7 on children and young people. Lancet Child Adolesc Health. 2021;5(4):e9-e10. doi: 10.1016/S2352-4642(21)00030-4.

35. Oliveira EA, Simoes ESAC, Oliveira MCL, Colosimo EA, Mak RH, Vasconcelos MA, et al. Comparison of the First and Second Waves of the Coronavirus Disease 2019 (COVID-19) Pandemic in Children and Adolescents in a Middle-Income Country: Clinical Impact Associated with Severe Acute Respiratory Syndrome Coronavirus 2 (SARS-CoV-2) Gamma Lineage. J Pediatr. 2022. doi: 10.1016/j.jpeds.2022.01.001.

36. Edward PR, Lorenzo-Redondo R, Reyna ME, Simons LM, Hultquist JF, Patel AB, et al. Severity of Illness Caused by Severe Acute Respiratory Syndrome Coronavirus 2 Variants of Concern in Children: A Single-Center Retrospective Cohort Study. medRxiv. 2021. doi: 10.1101/2021.10.23.21265402.

For peer review only - http://bmjopen.bmj.com/site/about/guidelines.xhtml

37. Murillo-Zamora E, Trujillo X, Huerta M, Rios-Silva M, Baltazar-Rodriguez LM, Guzman-Esquivel J, et al. Decreased risk of COVID-19 pneumonia in children and adolescents during the Delta variant emergence. Public Health. 2022;204:9-11. doi: 10.1016/j.puhe.2021.12.017.

38. Fisman DN, Tuite AR. Age-Specific Changes in Virulence Associated with SARS-CoV-2 Variants of Concern. Clin Infect Dis. 2022. doi: 10.1093/cid/ciac174.

39. Shoji K, Akiyama T, Tsuzuki S, Matsunaga N, Asai Y, Suzuki S, et al. Comparison of the clinical characteristics and outcomes of COVID-19 in children before and after the emergence of Delta variant of concern in Japan. J Infect Chemother. 2022;28(4):591-4. doi: 10.1016/j.jiac.2022.01.009.

40. Martin B, DeWitt PE, Russell S, Anand A, Bradwell KR, Bremer C, et al. Characteristics, Outcomes, and Severity Risk Factors Associated With SARS-CoV-2 Infection Among Children in the US National COVID Cohort Collaborative. JAMA Netw Open. 2022;5(2):e2143151. doi: 10.1001/jamanetworkopen.2021.43151.

41. Yilmaz Celebi M, Kiymet E, Boncuoglu E, Sahinkaya S, Cem E, Duzgol M, et al. Evaluation of childhood hospitalization rates and degree of severity of SARS-CoV-2 variants, including B.1.1.7 (Alpha), B.1.315/P.1 (Beta/Gamma), and B.1.617.2 (Delta). J Med Virol. 2022;94(5):2050-4. doi: 10.1002/jmv.27587.

42. Wang L, Berger NA, Kaelber DC, Davis PB, Volkow ND, Xu R. COVID infection severity in children under 5 years old before and after Omicron emergence in the US. medRxiv. 2022. doi: 10.1101/2022.01.12.22269179.

43. Wang L, Berger NA, Kaelber DC, Davis PB, Volkow ND, Xu R. COVID infection rates, clinical outcomes, and racial/ethnic and gender disparities before and after Omicron emerged in the US. medRxiv. 2022. doi: 10.1101/2022.02.21.22271300.

44. Marks KJ, Whitaker M, Anglin O, Milucky J, Patel K, Pham H, et al. Hospitalizations of Children and Adolescents with Laboratory-Confirmed COVID-19 - COVID-NET, 14 States, July 2021-January 2022. MMWR Morb Mortal Wkly Rep. 2022;71(7):271-8. doi: 10.15585/mmwr.mm7107e4.

45. Iuliano AD, Brunkard JM, Boehmer TK, Peterson E, Adjei S, Binder AM, et al. Trends in Disease Severity and Health Care Utilization During the Early Omicron Variant Period Compared with Previous SARS-CoV-2 High Transmission Periods - United States, December 2020-January 2022. MMWR Morb Mortal Wkly Rep. 2022;71(4):146-52. doi: 10.15585/mmwr.mm7104e4.

46. Shi DS, Whitaker M, Marks KJ, Anglin O, Milucky J, Patel K, et al. Hospitalizations of Children Aged 5-11 Years with Laboratory-Confirmed COVID-19 - COVID-NET, 14 States, March 2020-February 2022. MMWR Morb Mortal Wkly Rep. 2022;71(16):574-81. doi: 10.15585/mmwr.mm7116e1.

47. Marks KJ, Whitaker M, Agathis NT, Anglin O, Milucky J, Patel K, et al. Hospitalization of Infants and Children Aged 0-4 Years with Laboratory-Confirmed COVID-19 - COVID-NET, 14 States, March 2020-February 2022. MMWR Morb Mortal Wkly Rep. 2022;71(11):429-36.

48. Nygaard U, Holm M, Hartling UB, Glenthoj J, Schmidt LS, Nordly SB, et al. Incidence and clinical phenotype of multisystem inflammatory syndrome in children

For peer review only - http://bmjopen.bmj.com/site/about/guidelines.xhtml

after infection with the SARS-CoV-2 delta variant by vaccination status: a Danish nationwide prospective cohort study. Lancet Child Adolesc Health. 2022;6(7):459-65.

49. Baker JM, Buchfellner M, Britt W, Sanchez V, Potter JL, Ingram LA, et al. Acute Hepatitis and Adenovirus Infection Among Children - Alabama, October 2021-February 2022. MMWR Morb Mortal Wkly Rep. 2022;71(18):638-40.

50. Ng OT, Koh V, Chiew CJ, Marimuthu K, Thevasagayam NM, Mak TM, et al. Impact of SARS-CoV-2 Vaccination and Paediatric Age on Delta Variant Household Transmission. Clin Infect Dis. 2022.

51. Dougherty K, Mannell M, Naqvi O, Matson D, Stone J. SARS-CoV-2 B.1.617.2 (Delta) Variant COVID-19 Outbreak Associated with a Gymnastics Facility - Oklahoma, April-May 2021. MMWR Morb Mortal Wkly Rep. 2021;70(28):1004-7. doi: 10.15585/mmwr.mm7028e2.

52. Singanayagam A, Hakki S, Dunning J, Madon KJ, Crone MA, Koycheva A, et al. Community transmission and viral load kinetics of the SARS-CoV-2 delta (B.1.617.2) variant in vaccinated and unvaccinated individuals in the UK: a prospective, longitudinal, cohort study. Lancet Infect Dis. 2022;22(2):183-95. doi: 10.1016/S1473-3099(21)00648-4.

53. Schenk B, Hoehl S, Rudych O, Menger D, Farmand S, Wrobel F, et al. Longitudinal testing for SARS-CoV-2 RNA in day care centers in Hesse, Germany, during increased local incidence and with VOC Alpha as dominant variant: Results of the SAFE KiDS 2 and SAFE KiDS 3 study. MedRxiv. 2021. doi: 10.1101/2021.06.29.21259633.

54. Neuberger F, Grgic M, Diefenbacher S, Spensberger F, Lehfeld A-S, Buchholz U, et al. COVID-19 infections in day care centres in Germany: Social and organizational determinants of infections in children and staff in the second and third wave of the pandemic. MedRxiv. 2021. doi: 10.1101/2021.06.07.21257958.

55. Loenenbach A, Markus I, Lehfeld AS, An der Heiden M, Haas W, Kiegele M, et al. SARS-CoV-2 variant B.1.1.7 susceptibility and infectiousness of children and adults deduced from investigations of childcare centre outbreaks, Germany, 2021. Euro Surveill. 2021;26(21). doi: 10.2807/1560-7917.ES.2021.26.21.2100433.

56. Lorthe E, Bellon M, Michielin G, Berthelot J, Zaballa M-E, Pennacchio F, et al. Epidemiological, virological and serological investigation into a SARS-CoV-2 outbreak (Alpha variant) in a primary school: a prospective longitudinal study. medRxiv. 2021. doi: 10.1101/2021.10.26.21265509.

57. Abu-Raddad LJ, Chemaitelly H, Ayoub HH, Coyle P, Malek JA, Ahmed AA, et al. Introduction and expansion of the SARS-CoV-2 B.1.1.7 variant and reinfections in Qatar: A nationally representative cohort study. PLoS Med. 2021;18(12):e1003879. doi: 10.1371/journal.pmed.1003879.

58. Clifford S, Waight P, Hackman J, Hué S, Gower CM, Kirsebom FCM, et al. Effectiveness of BNT162b2 and ChAdOx1 against SARS-CoV-2 household transmission - a prospective cohort study in England. MedRxiv. 2021. doi: 10.1101/2021.11.24.21266401.

For peer review only - http://bmjopen.bmj.com/site/about/guidelines.xhtml

59. Lorthe E, Bellon M, Berthelot J, Michielin G, L'Huillier AG, Posfay-Barbe KM, et al. A SARS-CoV-2 omicron (B.1.1.529) variant outbreak in a primary school in Geneva, Switzerland. Lancet Infect Dis. 2022;22(6):767-8.

60. Lyngse FP, Molbak K, Skov RL, Christiansen LE, Mortensen LH, Albertsen M, et al. Increased transmissibility of SARS-CoV-2 lineage B.1.1.7 by age and viral load. Nat Commun. 2021;12(1):7251. doi: 10.1038/s41467-021-27202-x.

61. Chudasama DY, Flannagan J, Collin SM, Charlett A, Twohig KA, Lamagni T, et al. Household clustering of SARS-CoV-2 variant of concern B.1.1.7 (VOC-202012-01) in England. J Infect. 2021;83(1):e26-e8. doi: 10.1016/j.jinf.2021.04.029.

62. Loss J, Wurm J, Varnaccia G, Schienkiewitz A, Iwanowski H, Loer A-KM, et al. Transmission of SARS-CoV-2 among children and staff in German daycare centers: results from the COALA study. MedRxiv. 2021. doi: 10.1101/2021.12.21.21268157.

63. Buchan SA, Tibebu S, Daneman N, Whelan M, Vanniyasingam T, Murti M, et al. Increased Household Secondary Attacks Rates With Variant of Concern Severe Acute Respiratory Syndrome Coronavirus 2 Index Cases. Clin Infect Dis. 2022;74(4):703-6. doi: 10.1093/cid/ciab496.

64. Allen H, Vusirikala A, Flannagan J, Twohig KA, Zaidi A, Chudasama D, et al. Household transmission of COVID-19 cases associated with SARS-CoV-2 delta variant (B.1.617.2): national case-control study. Lancet Reg Health Eur. 2022;12:100252. doi: 10.1016/j.lanepe.2021.100252.

65. Trobajo-Sanmartin C, Martinez-Baz I, Miqueleiz A, Fernandez-Huerta M, Burgui C, Casado I, et al. Differences in Transmission between SARS-CoV-2 Alpha (B.1.1.7) and Delta (B.1.617.2) Variants. Microbiol Spectr. 2022;10(2):e0000822.

66. Lindstrom JC, Engebretsen S, Kristoffersen AB, Ro GOI, Palomares AD, Engo-Monsen K, et al. Increased transmissibility of the alpha SARS-CoV-2 variant: evidence from contact tracing data in Oslo, January to February 2021. Infectious diseases (London, England). 2022;54(1):72-7. doi: 10.1080/23744235.2021.1977382.

67. Julin CH, Robertson AH, Hungnes O, Tunheim G, Bekkevold T, Laake I, et al. Household Transmission of SARS-CoV-2: A Prospective Longitudinal Study Showing Higher Viral Load and Increased Transmissibility of the Alpha Variant Compared to Previous Strains. Microorganisms. 2021;9(11). doi: 10.3390/microorganisms9112371.

68. Ratmann O, Bhatt S, Flaxman S. Implications of a highly transmissible variant of SARS-CoV-2 for children. Arch Dis Child. 2021;106(9):e37. doi: 10.1136/archdischild-2021-321903.

69. Pedersen ESL, Collaud ENR, Mozun R, Dexter K, Kruljac C, Silberschmidt H, et al. Facemask Usage Among People With Primary Ciliary Dyskinesia During the COVID-19 Pandemic: A Participatory Project. Int J Public Health. 2021;66:1604277.

70. Lin DY, Gu Y, Wheeler B, Young H, Holloway S, Sunny SK, et al. Effectiveness of Covid-19 Vaccines over a 9-Month Period in North Carolina. N Engl J Med. 2022;386(10):933-41.

71. Eyre DW, Taylor D, Purver M, Chapman D, Fowler T, Pouwels KB, et al. Effect of Covid-19 Vaccination on Transmission of Alpha and Delta Variants. N Engl J Med. 2022;386(8):744-56.

For peer review only - http://bmjopen.bmj.com/site/about/guidelines.xhtml

72. Price AM, Olson SM, Newhams MM, Halasa NB, Boom JA, Sahni LC, et al. BNT162b2 Protection against the Omicron Variant in Children and Adolescents. N Engl J Med. 2022;386(20):1899-909.

73. Tan SHX, Cook AR, Heng D, Ong B, Lye DC, Tan KB. Effectiveness of BNT162b2 Vaccine against Omicron in Children 5 to 11 Years of Age. N Engl J Med. 2022;387(6):525-32.

74. Sacco C, Del Manso M, Mateo-Urdiales A, Rota MC, Petrone D, Riccardo F, et al. Effectiveness of BNT162b2 vaccine against SARS-CoV-2 infection and severe COVID-19 in children aged 5-11 years in Italy: a retrospective analysis of January-April, 2022. Lancet. 2022;400(10346):97-103.

75. Hunter DJ, Abdool Karim SS, Baden LR, Farrar JJ, Hamel MB, Longo DL, et al. Addressing Vaccine Inequity - Covid-19 Vaccines as a Global Public Good. N Engl J Med. 2022;386(12):1176-9.

76. Food and Drug Administration. Coronavirus (COVID-19) Update: FDA Authorizes Moderna and Pfizer-BioNTech COVID-19 Vaccines for Children Down to 6 Months of Age 2022 [Available from: https://www.fda.gov/news-events/press-announcements/coronavirus-covid-19-update-fda-authorizes-moderna-and-pfizer-biontech-covid-19-vaccines-children.]

77. GOV.UK. Pfizer/BioNTech COVID-19 vaccine authorised for use in infants and children aged 6 months to 4 years 2022 [Available from: https://www.gov.uk/government/news/pfizerbiontech-covid-19-vaccine-authorised-for-use-in-infants-and-children-aged-6-months-to-4-years.]

FIGURE TITLES AND LEGENDS

Figure 1. Flow chart
Of the 2407 studies found via database searches 53 were found to be eligible for this systematic review (including one study recommended to us by experts).

Figure 2. Map of the number of studies conducted in each country
Most studies came from high-income countries (n = 47, 89%). Six studies (11%) were from upper-middle-income countries.

For peer review only - http://bmjopen.bmj.com/site/about/guidelines.xhtml

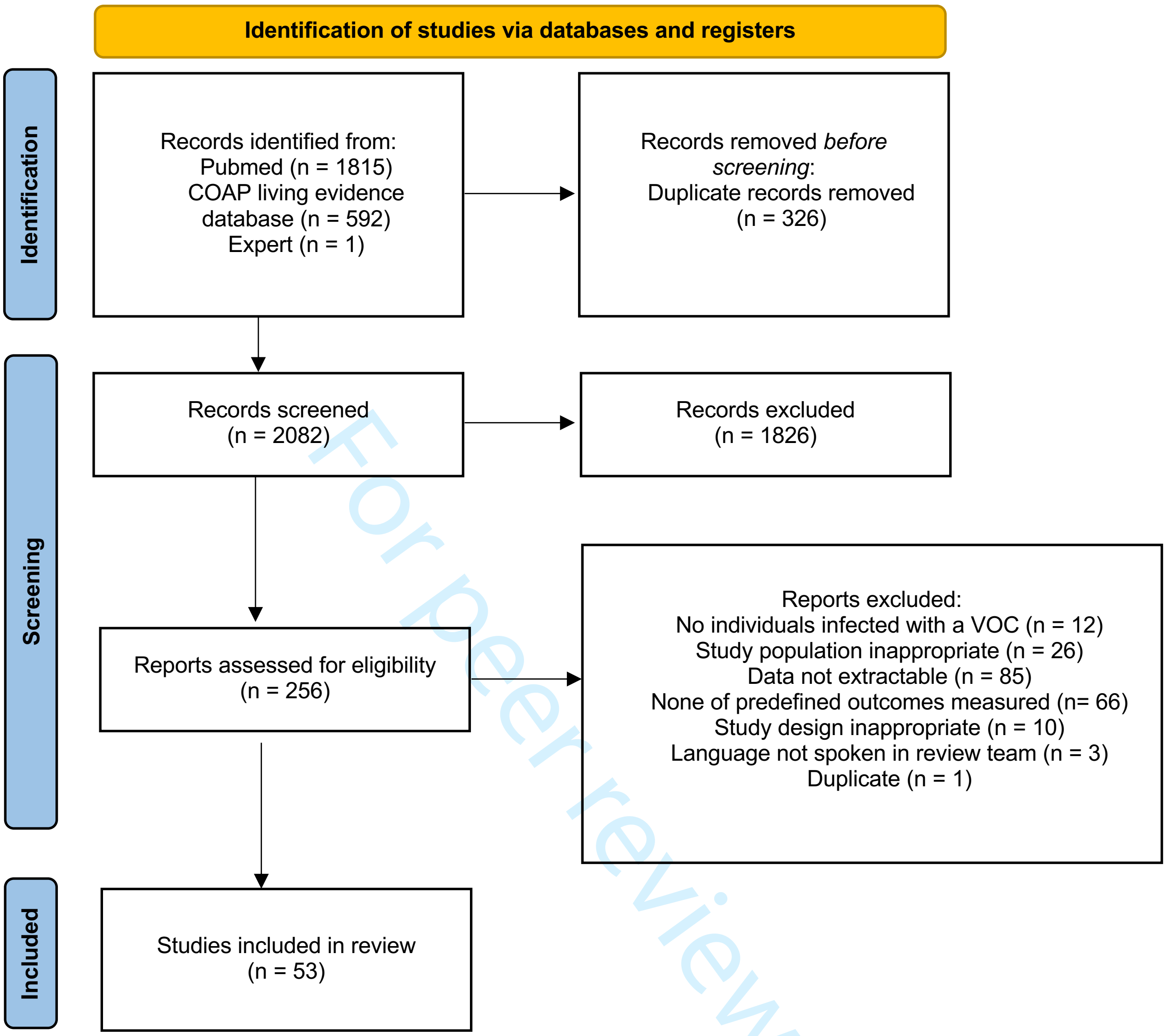

For peer review only

Figure 1: Flow chart
Of the 2407 studies found via database searches 53 were found to be eligible for this systematic review (incl. 1 study recommended to us by experts).

From: Page MJ, McKenzie JE, Bossuyt PM, Boutron I, Hoffmann TC, Mulrow CD, et al. The PRISMA 2020 statement: an updated guideline for reporting systematic reviews. BMJ 2021;372:n71. doi: 10.1136/bmj.n71

For more information, visit: http://www.prisma-statement.org/

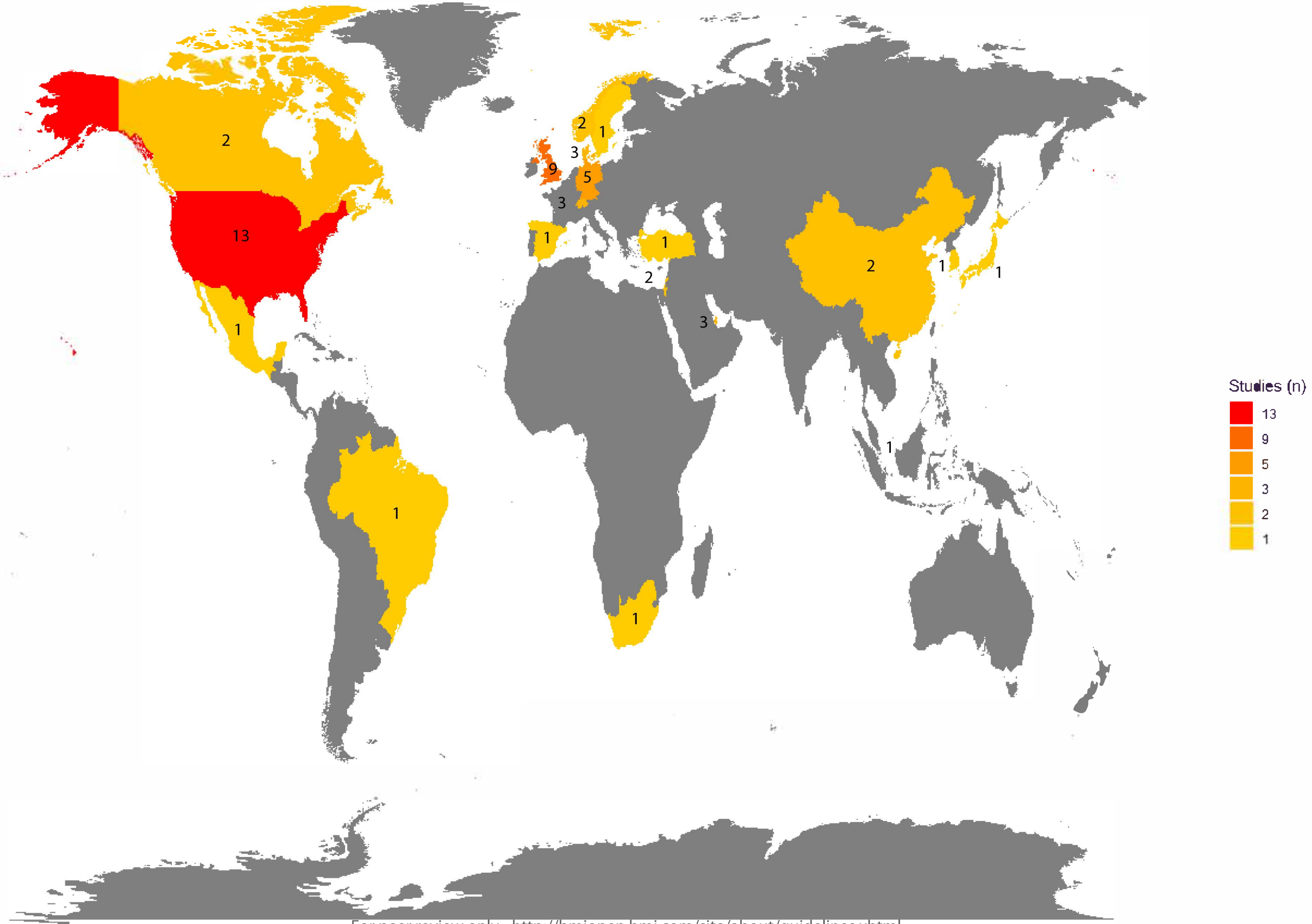

For peer review only - http://bmjopen.bmj.com/site/about/guidelines.xhtml

Figure 2. Map of the number of studies conducted in each country
Most studies came from high-income countries (n = 47, 89%). 6 studies (11%) were from upper-middle income countries.

Search strategy

1. COAP database (https://zika.ispm.unibe.ch/assets/data/pub/search_beta/)
   searched from: 1 January 2020 to 1 March 2022 - the database was not updated after 28 February 2022
   search query: child* AND variant*
   search field: Title OR Abstract

2. PubMed
   a) Search strategy from 1 August 2020 to 7 March 2022.
      Publication date filter:
      1 August 2020 to 30 January 2022: none (publications from before 1 August 2020 were manually removed afterwards)
      31 January to 7 March 2022: 2022/1/20- 2022/3/7

      Search query:
      “child*"[Title/Abstract] OR "neonat*"[Title/Abstract] OR "adolesc*"[Title/Abstract] OR "infan*"[Title/Abstract] OR "teen*"[Title/Abstract] OR "pediatr*"[Title/Abstract] OR "paediatr*"[Title/Abstract] OR ("educational status"[MeSH Terms] OR ("educational"[Title/Abstract] AND "status"[Title/Abstract]) OR "educational status"[Title/Abstract] OR "schooling"[Title/Abstract] OR "education"[MeSH Terms] OR "education"[Title/Abstract] OR "school s"[Title/Abstract] OR "schooled"[Title/Abstract] OR "schools"[MeSH Terms] OR "schools"[Title/Abstract] OR "school"[Title/Abstract]) OR ("nurseries, infant"[MeSH Terms] OR ("nurseries"[Title/Abstract] AND "infant"[Title/Abstract]) OR "infant nurseries"[Title/Abstract] OR "nurseries"[Title/Abstract] OR "nursery"[Title/Abstract]) OR ("infant, newborn"[MeSH Terms] OR ("infant"[Title/Abstract] AND "newborn"[Title/Abstract]) OR "newborn infant"[Title/Abstract] OR "newborn"[Title/Abstract] OR "newborns"[Title/Abstract] OR "newborns"[Title/Abstract]) OR "toddler*"[Title/Abstract] OR ("minority groups"[MeSH Terms] OR ("minority"[Title/Abstract] AND "groups"[Title/Abstract]) OR "minority groups"[Title/Abstract] OR "minorities"[Title/Abstract] OR "minority"[Title/Abstract] OR "minority s"[Title/Abstract] OR "minors"[MeSH Terms] OR "minors"[Title/Abstract] OR "minor"[Title/Abstract]) AND “COVID-19”[tiab] OR 2019-novel-cov*[tiab] OR coronavirus[tiab] OR coronavirusdisease-19*[tiab] OR corona-virus-disease-19*[tiab] OR covid-19*[tiab] OR covid19*[tiab] OR new-coronavirus[tiab] OR new-corona-virus[tiab] OR novelcoronavirus[tiab] OR novel-corona-virus[tiab] OR sars-2*[tiab] OR sars2*[tiab] OR sars-cov-19*[tiab] OR sarscov19*[tiab] OR sars-cov-2*[tiab] OR sars-cov2*[tiab] OR sarscov2*[tiab] OR sarscov-2*[tiab] AND "VOC"[All Fields] OR "variant of concern"[All Fields] OR "alpha variant"[All Fields] OR "beta variant"[All Fields] OR "gamma variant"[All Fields] OR "delta variant"[All Fields] OR “omicron variant”[All Fields] OR "variant of interest"[All Fields]

   b) Search strategy from 8 March 2022 to 9 May 2022
      Publication date filter: 2022/3/1- 2022/5/9

For peer review only - http://bmjopen.bmj.com/site/about/guidelines.xhtml

<u>Search query:</u>
(("Alpha"[Title/Abstract] OR "Beta"[Title/Abstract] OR "Gamma"[Title/Abstract] OR "Omicron"[Title/Abstract] OR "Delta"[Title/Abstract]) AND "variant"[Title/Abstract]) OR "B.1.1.529"[Title/Abstract] OR "B.1.617.2"[Title/Abstract] OR "B.1.1.7"[Title/Abstract] OR "B.1.351"[Title/Abstract] AND ((((((((child*) OR (neonat*)) OR (adolesc*)) OR (infant*)) OR (teen*)) OR (pediatr*)) OR (paediatr*)) OR (toddler*)) OR (newborn*)

The date filters applied in the last 2 searches overlapped on purpose to reduce the risk of us missing any publications due to their publication date.

For peer review only

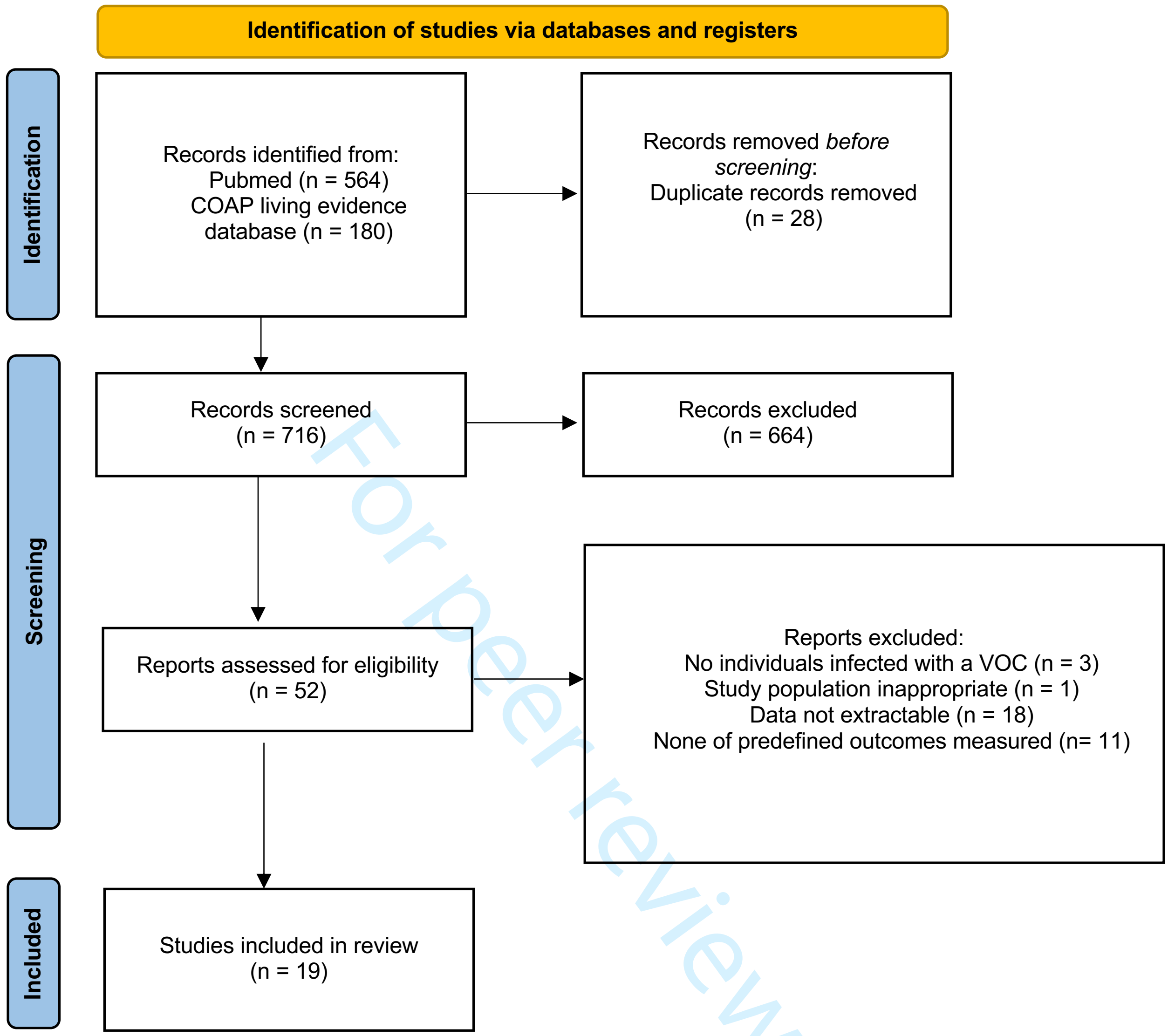

For peer review only

Supplementary figure 1: Flow chart – first search (until 15.10.2021)
Of the 744 studies found via database searches 19 were found to be eligible for this systematic review.

From: Page MJ, McKenzie JE, Bossuyt PM, Boutron I, Hoffmann TC, Mulrow CD, et al. The PRISMA 2020 statement: an updated guideline for reporting systematic reviews. BMJ 2021;372:n71. doi: 10.1136/bmj.n71

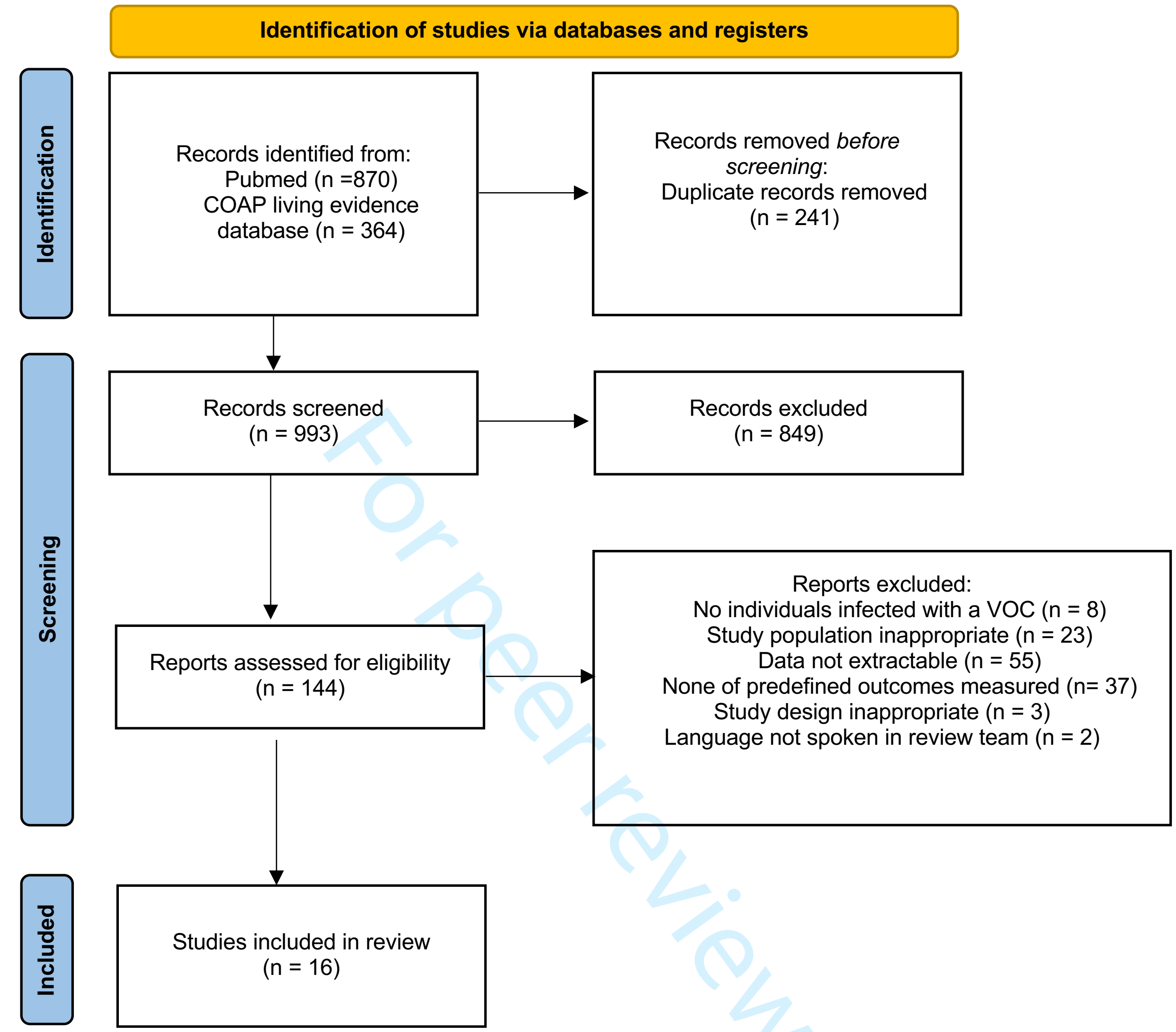

Supplementary figure 2: Flow chart – second search (15.10.2021-31.01.2022)
Of the 1234 studies found via database searches 16 were found to be eligible for this systematic review.

From: Page MJ, McKenzie JE, Bossuyt PM, Boutron I, Hoffmann TC, Mulrow CD, et al. The PRISMA 2020 statement: an updated guideline for reporting systematic reviews. BMJ 2021;372:n71. doi: 10.1136/bmj.n71

For peer review only

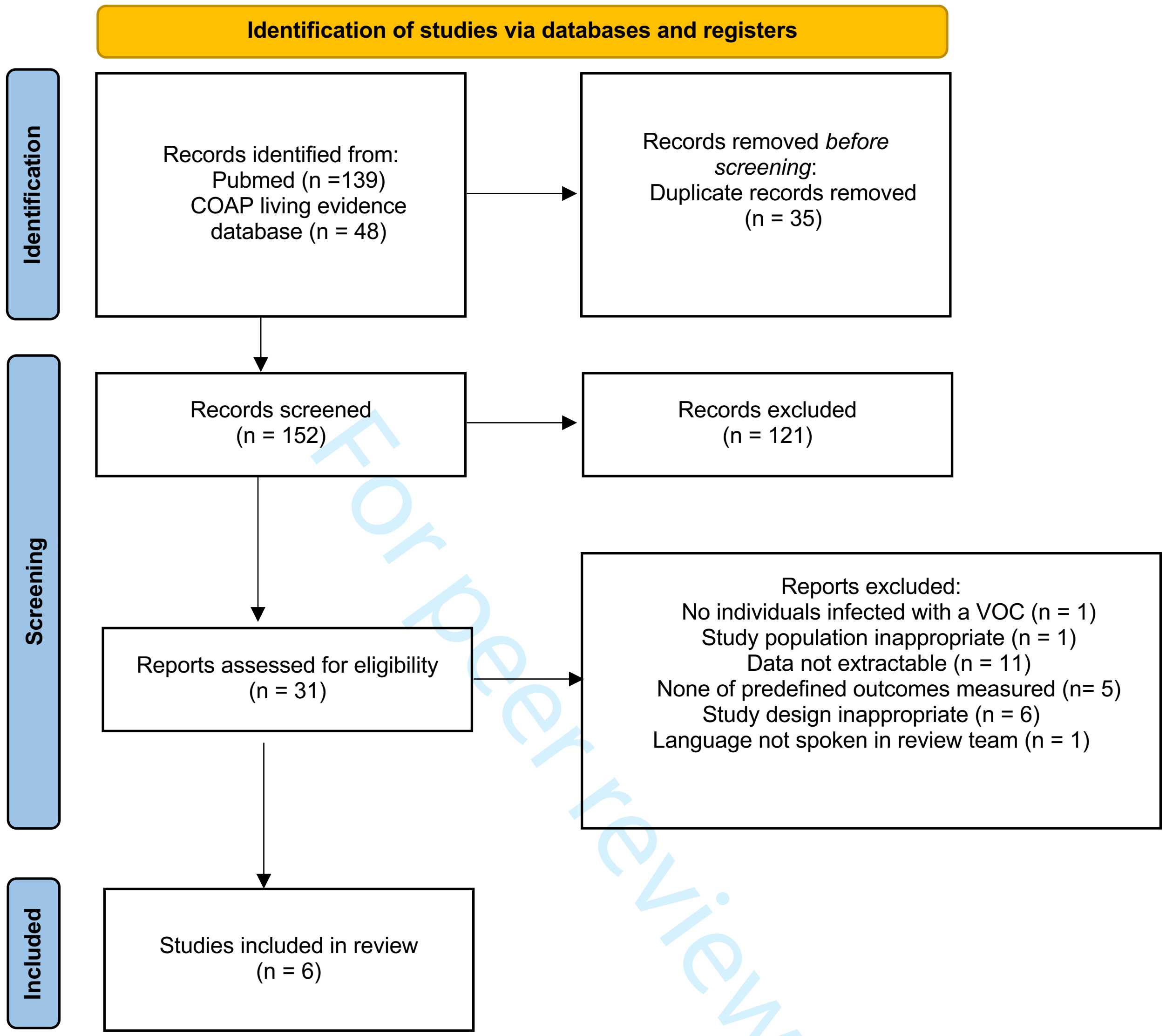

Supplementary figure 3: Flow chart – third search (31.01.2022-07.03.2022)
Of the 187 studies found via database searches 6 were found to be eligible for this systematic review.

From: Page MJ, McKenzie JE, Bossuyt PM, Boutron I, Hoffmann TC, Mulrow CD, et al. The PRISMA 2020 statement: an updated guideline for reporting systematic reviews. BMJ 2021;372:n71. doi: 10.1136/bmj.n71

For peer review only

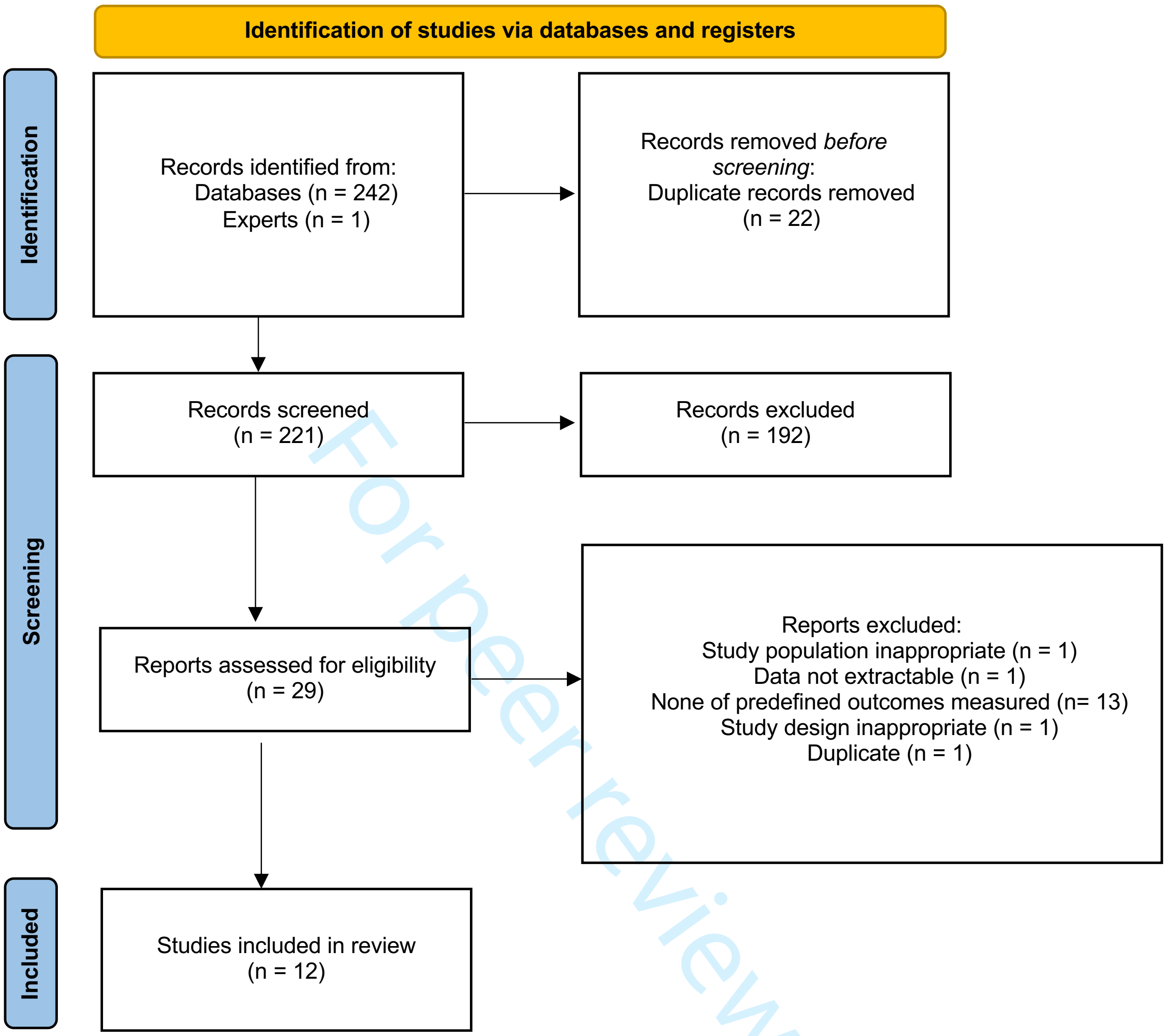

Supplementary figure 4: Flow chart – fourth search (07.03.2022-09.05.2022)
Of the 243 studies found via the database search (incl. 1 study recommended by experts) 12 studies were found to be eligible.

From: Page MJ, McKenzie JE, Bossuyt PM, Boutron I, Hoffmann TC, Mulrow CD, et al. The PRISMA 2020 statement: an updated guideline for reporting systematic reviews. BMJ 2021;372:n71. doi: 10.1136/bmj.n71

For more information, visit: http://www.prisma-statement.org/

For peer review only

PRISMA 2020 Checklist

Section and Topic	Item #	Checklist item	Location where item is reported
**TITLE**			
Title	1	Identify the report as a systematic review.	Title page
**ABSTRACT**			
Abstract	2	See the PRISMA 2020 for Abstracts checklist.	Abstract
**INTRODUCTION**			
Rationale	3	Describe the rationale for the review in the context of existing knowledge.	Introduction
Objectives	4	Provide an explicit statement of the objective(s) or question(s) the review addresses.	Introduction
**METHODS**			
Eligibility criteria	5	Specify the inclusion and exclusion criteria for the review and how studies were grouped for the syntheses.	Methods - Eligibility and screening/Data synthesis
Information sources	6	Specify all databases, registers, websites, organisations, reference lists and other sources searched or consulted to identify studies. Specify the date when each source was last searched or consulted.	Methods - Search strategy
Search strategy	7	Present the full search strategies for all databases, registers and websites, including any filters and limits used.	Supplementary note 1
Selection process	8	Specify the methods used to decide whether a study met the inclusion criteria of the review, including how many reviewers screened each record and each report retrieved, whether they worked independently, and if applicable, details of automation tools used in the process.	Methods - Eligibility and screening
Data collection process	9	Specify the methods used to collect data from reports, including how many reviewers collected data from each report, whether they worked independently, any processes for obtaining or confirming data from study investigators, and if applicable, details of automation tools used in the process.	Methods - Data extraction/Risk of bias assessment
Data items	10a	List and define all outcomes for which data were sought. Specify whether all results that were compatible with each outcome domain in each study were sought (e.g. for all measures, time points, analyses), and if not, the methods used to decide which results to collect.	Methods - Data extraction/Risk of bias assessment
	10b	List and define all other variables for which data were sought (e.g. participant and intervention characteristics, funding sources). Describe any assumptions made about any missing or unclear information.	Methods - Data extraction/Risk of bias assessment
Study risk of bias assessment	11	Specify the methods used to assess risk of bias in the included studies, including details of the tool(s) used, how many reviewers assessed each study and whether they worked independently, and if applicable, details of automation tools used in the process.	Methods - Risk of bias assessment
Effect measures	12	Specify for each outcome the effect measure(s) (e.g. risk ratio, mean difference) used in the synthesis or presentation of results.	n/a
Synthesis	13a	Describe the processes used to decide which studies were eligible for each synthesis (e.g. tabulating the study intervention characteristics	Methods -

For peer review only - http://bmjopen.bmj.com/site/about/guidelines.xhtml

PRISMA 2020 Checklist

Section and Topic	Item #	Checklist item	Location where item is reported
methods		and comparing against the planned groups for each synthesis (item #5)).	Data synthesis
	13b	Describe any methods required to prepare the data for presentation or synthesis, such as handling of missing summary statistics, or data conversions.	Methods - Data synthesis
	13c	Describe any methods used to tabulate or visually display results of individual studies and syntheses.	Methods - Data synthesis
	13d	Describe any methods used to synthesize results and provide a rationale for the choice(s). If meta-analysis was performed, describe the model(s), method(s) to identify the presence and extent of statistical heterogeneity, and software package(s) used.	Methods - Data synthesis
	13e	Describe any methods used to explore possible causes of heterogeneity among study results (e.g. subgroup analysis, meta-regression).	n/a
	13f	Describe any sensitivity analyses conducted to assess robustness of the synthesized results.	n/a
Reporting bias assessment	14	Describe any methods used to assess risk of bias due to missing results in a synthesis (arising from reporting biases).	Methods - Risk of bias assessment
Certainty assessment	15	Describe any methods used to assess certainty (or confidence) in the body of evidence for an outcome.	Methods - Risk of bias assessment
**RESULTS**			
Study selection	16a	Describe the results of the search and selection process, from the number of records identified in the search to the number of studies included in the review, ideally using a flow diagram.	Results, figure 1, supplementary figures 1-4
	16b	Cite studies that might appear to meet the inclusion criteria, but which were excluded, and explain why they were excluded.	Figure 1, supplementary figures 1-4
Study characteristics	17	Cite each included study and present its characteristics.	Supplementary tables 7-12
Risk of bias in studies	18	Present assessments of risk of bias for each included study.	Supplementary tables 2-6
Results of individual studies	19	For all outcomes, present, for each study: (a) summary statistics for each group (where appropriate) and (b) an effect estimate and its precision (e.g. confidence/credible interval), ideally using structured tables or plots.	Supplementary tables 7-12
Results of syntheses	20a	For each synthesis, briefly summarise the characteristics and risk of bias among contributing studies.	Discussion - Interpretation and implications of findings
	20b	Present results of all statistical syntheses conducted. If meta-analysis was done, present for each the summary estimate and its precision (e.g. confidence/credible interval) and measures of statistical heterogeneity. If comparing groups, describe the direction of the effect.	n/a
	20c	Present results of all investigations of possible causes of heterogeneity among study results.	Results
	20d	Present results of all sensitivity analyses conducted to assess the robustness of the synthesized results.	n/a

For peer review only

For peer review only - http://bmjopen.bmj.com/site/about/guidelines.xhtml

PRISMA 2020 Checklist

Section and Topic	Item #	Checklist item	Location where item is reported
Reporting biases	21	Present assessments of risk of bias due to missing results (arising from reporting biases) for each synthesis assessed.	n/a
Certainty of evidence	22	Present assessments of certainty (or confidence) in the body of evidence for each outcome assessed.	Results
**DISCUSSION**			
Discussion	23a	Provide a general interpretation of the results in the context of other evidence.	Discussion
	23b	Discuss any limitations of the evidence included in the review.	Discussion - Strengths and limitations
	23c	Discuss any limitations of the review processes used.	Discussion - Strengths and limitations
	23d	Discuss implications of the results for practice, policy, and future research.	Discussion
**OTHER INFORMATION**			
Registration and protocol	24a	Provide registration information for the review, including register name and registration number, or state that the review was not registered.	Abstract, Methods - Design
	24b	Indicate where the review protocol can be accessed, or state that a protocol was not prepared.	Methods - Design
	24c	Describe and explain any amendments to information provided at registration or in the protocol.	n/a
Support	25	Describe sources of financial or non-financial support for the review, and the role of the funders or sponsors in the review.	Funding
Competing interests	26	Declare any competing interests of review authors.	Competing interests
Availability of data, code and other materials	27	Report which of the following are publicly available and where they can be found: template data collection forms; data extracted from included studies; data used for all analyses; analytic code; any other materials used in the review.	extracted and used data: supplementary tables 2-12

From: Page MJ, McKenzie JE, Bossuyt PM, Boutron I, Hoffmann TC, Mulrow CD, et al. The PRISMA 2020 statement: an updated guideline for reporting systematic reviews. BMJ 2021;372:n71. doi: 10.1136/bmj.n71

For more information, visit: http://www.prisma-statement.org/

For peer review only

Supplementary table 2: Risk of bias assessment - Child/adolescent symptoms/severity of disease when infected with a VOC

Cohort studies[1]	Exposed and non-exposed cohorts from the same population	Confidence in exposure assessment	Confidence that outcome of interest was not present at start of study	Matching of exposed and unexposed for all variables associated with outcome of interest or adjustment of prognostic variables by statistical analysis	Confidence in assessment of presence/absence of prognostic factors	Confidence in outcome assessment	Adequate follow-up of cohorts	Similar co-interventions between groups
Ulyte et al. [16]	definitely yes	probably no	definitely no	probably no	probably yes	probably no	definitely no	definitely yes
Nakel et al. [19]	probably yes	definitely yes	probably yes	definitely no	definitely no	probably no	definitely no	probably yes
Somekh et al. [33]	probably yes	definitely yes	probably yes	probably no	probably no	definitely yes	probably yes	probably no
Swann et al. [20]	definitely yes	definitely yes	probably no	probably yes	definitely yes	definitely yes	probably yes	probably yes
Edward et al. [28]	probably yes	definitely yes	definitely yes	probably yes	probably no	definitely yes	probably yes	probably no
Celebi et al. [41]	probably yes	probably yes	definitely yes	definitely no	definitely no	probably yes	probably no	definitely no
Stewart et al. [21]	probably yes	definitely yes	probably yes	probably no	probably no	definitely yes	definitely yes	probably no
Brookman et al. [34]	probably yes	definitely yes	definitely yes	definitely no	definitely no	probably yes	probably no	probably yes
Li et al. [22]	definitely yes	definitely yes	probably yes	definitely yes	probably no	probably yes	definitely yes	definitely yes
Shoji et al. [39]	definitely yes	definitely yes	definitely yes	probably no	probably yes	definitely yes	probably yes	probably no
Fisman et al. [38]	probably yes	definitely yes	definitely yes	probably yes	probably yes	definitely yes	probably yes	probably yes
Ryu et al. [24]	probably yes	definitely yes	probably yes	definitely no	definitely no	probably yes	probably no	probably no
Martin et al. [40]	probably yes	probably yes	definitely yes	probably no	probably no	definitely yes	probably no	definitely no
Molteni et al. [26]	definitely yes	probably yes	probably yes	probably yes	probably no	probably yes	probably yes	probably no
Murillo-Zamora et al. [37]	probably yes	definitely yes	definitely yes	definitely no	probably no	definitely yes	probably no	probably no
Butt et al. [25]	probably yes	probably yes	probably yes	probably yes	probably no	definitely yes	probably no	probably no
Oliveira et al. [35]	probably yes	definitely yes	definitely yes	definitely no	definitely no	definitely yes	probably no	probably no
Cloete et al. [29]	probably yes	probably yes	probably yes	probably yes	probably yes	probably yes	definitely yes	definitely yes
Wang et al. [42]	probably yes	probably yes	probably yes	probably yes	probably yes	definitely yes	probably no	probably no
Wang et al. [43]	probably yes	probably yes	probably yes	probably no	probably no	definitely yes	probably no	probably no

[1] CLARITY Group at McMaster University. Tool to Assess Risk of Bias in Cohort Studies [Available from: https://www.evidencepartners.com/wp-content/uploads/2021/03/Tool-to-Assess-Risk-of-Bias-in-Cohort-Studies-DistillerSR.pdf.

For peer review only

Marks et al. [44]	definitely yes	definitely yes	definitely yes	definitely no	definitely no	probably no	definitely yes	probably no
Butt et al. [31]	probably yes	definitely yes	probably yes	probably yes	probably yes	definitely yes	definitely no	probably yes
Edward et al. [36]	probably yes	definitely yes	definitely yes	probably yes	probably no	definitely yes	probably yes	probably no
Kildegaard et al. [27]	probably yes	probably yes	probably yes	definitely no	probably no	definitely yes	definitely yes	probably yes
Fowlkes et al. [32]	definitely yes	probably yes	probably yes	probably no	definitely no	probably yes	probably yes	probably yes
Hao et al. [23]	probably yes	probably yes	probably yes	probably no	definitely no	probably yes	probably yes	probably yes
Brewster et al. [14]	definitely yes	definitely yes	definitely yes	definitely no	definitely yes	definitely yes	probably yes	probably no
Waltenburg et al. [18]	probably yes	probably yes	probably yes	probably no	probably no	probably yes	definitely no	probably yes
Marks et al. [47]	probably yes	probably no	probably yes	probably no	probably no	definitely yes	probably yes	probably yes
Nygaard et al. [48]	probably yes	probably yes	definitely yes	probably no	definitely no	definitely yes	definitely yes	probably yes

**Case control studies[2]**	**Confidence in exposure assessment**	**Confidence in cases but not controls acquiring outcome of interest**	**Proper selection of cases (those that were exposed and developed outcome of interest)**	**Proper selection of controls (those that were exposed and did not develop outcome of interest)**	**Matching of cases and controls according to prognostic variables or statistical adjustment for these variables**
Roberts et al. [15]	probably yes	probably yes	definitely yes	probably yes	definitely no

[2] CLARITY Group at McMaster University. Tool to Assess Risk of Bias in Case Control Studies [Available from: https://www.evidencepartners.com/wp-content/uploads/2021/03/Tool-to-Assess-Risk-of-Bias-in-Case-Control-Studies-DistillerSR.pdf.

For peer review only - http://bmjopen.bmj.com/site/about/guidelines.xhtml

**Cross sectional studies**	**Exposed and non-exposed groups from the same population**	**Confidence in exposure assessment**	**Confidence that outcome of interest was not present at start of study**	**Matching of exposed and unexposed for all variables associated with outcome of interest or adjustment of prognostic variables by statistical analysis**	**Confidence in assessment of presence/absence of prognostic factors**	**Confidence in outcome assessment**	**Similar co-interventions between groups**
Iuliano et al. [45]	probably yes	probably yes	definitely yes	definitely no	definitely no	definitely yes	probably no
Shi et al. [46]	definitely yes	definitely yes	probably yes	definitely no	definitely yes	definitely yes	probably yes
Somekh et al. [17]	probably yes	definitely yes	probably no	definitely no	probably no	definitely yes	probably yes

For peer review only

Supplementary table 3: Risk of bias assessment - Child/Adolescent risk of severe disease when infected with a VOC

Cohort studies[1]	Exposed and non-exposed cohorts from the same population	Confidence in exposure assessment	Confidence that outcome of interest was not present at start of study	Matching of exposed and unexposed for all variables associated with outcome of interest or adjustment of prognostic variables by statistical analysis	Confidence in assessment of presence/absence of prognostic factors	Confidence in outcome assessment	Adequate follow-up of cohorts	Similar co-interventions between groups
Butt et al. [31]	probably yes	definitely yes	probably yes	probably yes	probably yes	definitely yes	definitely no	probably yes

Cross sectional studies	Exposed and non-exposed groups from the same population	Confidence in exposure assessment	Confidence that outcome of interest was not present at start of study	Matching of exposed and unexposed for all variables associated with outcome of interest or adjustment of prognostic variables by statistical analysis	Confidence in assessment of presence/absence of prognostic factors	Confidence in outcome assessment	Similar co-interventions between groups
Shi et al. [46]	definitely yes	definitely yes	probably yes	definitely no	definitely yes	definitely yes	probably yes

[1] CLARITY Group at McMaster University. Tool to Assess Risk of Bias in Cohort Studies [Available from: https://www.evidencepartners.com/wp-content/uploads/2021/03/Tool-to-Assess-Risk-of-Bias-in-Cohort-Studies-DistillerSR.pdf.

For peer review only

Supplementary table 4: Risk of bias assessment - Child/Adolescent risk of becoming infected with a VOC

Cohort studies[1]	Exposed and non-exposed cohorts from the same population	Confidence in exposure assessment	Confidence that outcome of interest was not present at start of study	Matching of exposed and unexposed for all variables associated with outcome of interest or adjustment of prognostic variables by statistical analysis	Confidence in assessment of presence/absence of prognostic factors	Confidence in outcome assessment	Adequate follow-up of cohorts	Similar co-interventions between groups
Nakel et al. [19]	probably yes	definitely yes	probably yes	definitely no	definitely no	probably no	definitely no	probably yes
Somekh et al. [33]	probably yes	definitely yes	probably yes	probably no	probably no	definitely yes	probably yes	probably no
Abu Raddad et al. [57]	definitely yes	definitely yes	probably no	definitely no	probably no	probably yes	definitely yes	probably no
Loenenbach et al. [55]	probably yes	probably yes	probably no	definitely no	definitely no	definitely yes	probably no	probably no
Lorthe et al. [56]	definitely yes	definitely yes	probably yes	definitely no	probably yes	probably yes	definitely yes	definitely yes
Clifford et al. [58]	probably yes	probably yes	definitely yes	definitely no	definitely no	definitely yes	probably no	probably yes
Dougherty et al. [51]	probably yes	definitely yes	definitely yes	probably no	probably yes	definitely yes	probably yes	probably no
Singanayagam et al. [52]	probably yes	probably yes	probably yes	probably no	probably no	definitely yes	probably yes	probably no
Schenk et al. [53]	probably yes	probably yes	probably no	probably no	probably no	probably yes	probably no	probably yes
Lorthe et al. [59]	probably yes	definitely yes	definitely no	definitely no	definitely no	definitely yes	probably no	definitely no
Waltenburg et al. [18]	probably yes	probably yes	probably yes	probably no	probably no	probably yes	definitely no	probably yes
Ng et al. [50]	probably yes	probably yes	probably yes	probably no	definitely no	probably yes	probably yes	probably yes

[1] CLARITY Group at McMaster University. Tool to Assess Risk of Bias in Cohort Studies [Available from: https://www.evidencepartners.com/wp-content/uploads/2021/03/Tool-to-Assess-Risk-of-Bias-in-Cohort-Studies-DistillerSR.pdf.

For peer review only

Case control studies[2]	Confidence in exposure assessment	Confidence in cases but not controls acquiring outcome of interest	Proper selection of cases (those that were exposed and developed outcome of interest)	Proper selection of controls (those that were exposed and did not develop outcome of interest)	Matching of cases and controls according to prognostic variables or statistical adjustment for these variables
Roberts et al. [15]	probably yes	probably yes	definitely yes	probably yes	definitely no

Cross sectional studies	Exposed and non-exposed groups from the same population	Confidence in exposure assessment	Confidence that outcome of interest was not present at start of study	Matching of exposed and unexposed for all variables associated with outcome of interest or adjustment of prognostic variables by statistical analysis	Confidence in assessment of presence/absence of prognostic factors	Confidence in outcome assessment	Similar co-interventions between groups
Somekh et al. [17]	probably yes	definitely yes	probably no	definitely no	probably no	definitely yes	probably yes
Neuberger et al. [54]	probably yes	probably yes	probably yes	probably no	probably no	probably yes	probably yes

[2] CLARITY Group at McMaster University. Tool to Assess Risk of Bias in Case Control Studies [Available from: https://www.evidencepartners.com/wp-content/uploads/2021/03/Tool-to-Assess-Risk-of-Bias-in-Case-Control-Studies-DistillerSR.pdf.

For peer review only

Supplementary table 5: Risk of bias assessment - Child/adolescent risk of transmission when infected with a VOC

Cohort studies[1]	Exposed and non-exposed cohorts from the same population	Confidence in exposure assessment	Confidence that outcome of interest was not present at start of study	Matching of exposed and unexposed for all variables associated with outcome of interest or adjustment of prognostic variables by statistical analysis	Confidence in assessment of presence/absence of prognostic factors	Confidence in outcome assessment	Adequate follow-up of cohorts	Similar co-interventions between groups
Somekh et al. [33]	probably yes	definitely yes	probably yes	probably no	probably no	definitely yes	probably yes	probably no
Buchan et al. [63]	definitely yes	probably yes	probably no	definitely no	definitely no	probably yes	probably no	probably no
Lindstrom et al. [66]	definitely yes	definitely yes	definitely no	probably no	definitely no	probably yes	probably no	probably yes
Loenenbach et al. [55]	probably yes	probably yes	probably no	definitely no	definitely no	definitely yes	probably no	probably no
Lorthe et al. [56]	definitely yes	definitely yes	probably yes	definitely no	probably yes	probably yes	definitely yes	definitely yes
Julin et al. [67]	probably yes	definitely yes	definitely yes	probably no	definitely no	definitely yes	definitely yes	probably yes
Lyngse et al. [60]	definitely yes	definitely yes	definitely yes	probably yes	probably yes	probably yes	probably yes	probably no
Chudasama et al. [61]	probably yes	probably no	probably no	definitely no	definitely no	probably yes	probably no	definitely no
Lorthe et al. [59]	probably yes	definitely yes	definitely no	definitely no	definitely no	definitely yes	probably no	definitely no
Trobajo-Sanmartin et al. [65|	probably yes	definitely yes	probably yes	probably yes	probably yes	definitely yes	probably no	probably yes
Waltenburg et al. [18]	probably yes	probably yes	probably yes	probably no	probably no	probably yes	definitely no	probably yes
Ng et al. [50]	probably yes	probably yes	probably yes	probably no	definitely no	probably yes	probably yes	probably yes

[1] CLARITY Group at McMaster University. Tool to Assess Risk of Bias in Cohort Studies [Available from: https://www.evidencepartners.com/wp-content/uploads/2021/03/Tool-to-Assess-Risk-of-Bias-in-Cohort-Studies-DistillerSR.pdf.

For peer review only

Case control studies[2]	Confidence in exposure assessment	Confidence in cases but not controls acquiring outcome of interest	Proper selection of cases (those that were exposed and developed outcome of interest)	Proper selection of controls (those that were exposed and did not develop outcome of interest)	Matching of cases and controls according to prognostic variables or statistical adjustment for these variables
Loss et al. [62]	probably yes	definitely yes	definitely yes	definitely yes	probably no
Allen et al. [64]	definitely yes	definitely yes	definitely yes	definitely yes	probably yes

Modelling studies[3]	Is the population relevant?	Are any critical interven-tions missing?	Are any relevant out-comes missing?	Is the context (settings and circum-stances) applicable?	Is external validation of the model sufficient to make its results credible for your decision?	Is internal verification of the model sufficient to make its results credible for your decision?	Does the model have sufficient face validity to make its results credible for your decision?	Is the design of the model adequate for your decision problem?	Are the data used in populating the model suitable for your decision problem?	Were the analyses performed using the model adequate to inform your decision problem?	Was there an adequate assessment of the effects of uncertainty?	Was the reporting of the model adequate to inform your decision problem?	Was the interpre-tation of results fair and balanced?	Were there any potential conflicts of interest?	If there were potential conflicts of interest, were steps taken to address these?
Ratman et al. [68]	definitely yes	probably yes	probably no	probably yes	definitely no	definitely no	probably no	probably yes	probably yes	probably yes	definitely no	probably yes	probably yes	definitely no	probably no

[2] CLARITY Group at McMaster University. Tool to Assess Risk of Bias in Case Control Studies [Available from: https://www.evidencepartners.com/wp-content/uploads/2021/03/Tool-to-Assess-Risk-of-Bias-in-Case-Control-Studies-DistillerSR.pdf.
[3] Jaime Caro J, Eddy DM, Kan H, Kaltz C, Patel B, Eldessouki R, et al. Questionnaire to assess relevance and credibility of modeling studies for informing health care decision making: an ISPOR-AMCP-NPC Good Practice Task Force report. Value Health. 2014;17(2):174-82. doi: 10.1016/j.jval.2014.01.003.

For peer review only

Supplementary table 6: Risk of bias assessment - Risk of long-term effects when infected with a VOC

**Cohort studies**[7]	**Exposed and non-exposed cohorts from the same population**	**Confidence in exposure assessment**	**Confidence that outcome of interest was not present at start of study**	**Matching of exposed and unexposed for all variables associated with outcome of interest or adjustment of prognostic variables by statistical analysis**	**Confidence in assessment of presence/absence of prognostic factors**	**Confidence in outcome assessment**	**Adequate follow-up of cohorts**	**Similar co-interventions between groups**
Molteni et al. [38]	definitely yes	probably yes	probably yes	probably yes	probably no	probably yes	probably yes	probably no
Kildegaard et al. [27]	probably yes	probably yes	probably yes	definitely no	probably no	definitely yes	definitely yes	probably yes

For peer review only

[7]CLARITY Group at McMaster University. Tool to Assess Risk of Bias in Cohort Studies [Available from: https://www.evidencepartners.com/wp-content/uploads/2021/03/Tool-to-Assess-Risk-of-Bias-in-Cohort-Studies-DistillerSR.pdf.

Supplementary table 7: Review question 1 - Child/adolescent symptoms/severity of disease when infected with the alpha variant (n=9)

Author	Study design	Country	Study setting	Number of study participants <20 yo[1]	Number of adult study participants	Age groups	Mean/ Median age	VOC[2]	Symptoms/Severity	Comments
Roberts et al. [15]	case-control study	United Kingdom	schools kindergartens crèches	overall: 83<br><br>infected: 21	34	≤4yo, adults	n/r	Alpha	**Symptoms**<br>24% fever, 19% cough (52% asymptomatic)	**sequencing:** 1 case was sequenced, rest was assumed based on date of sampling and national prevalence
Ulyte et al. [16]	cohort study	Switzerland	schools kindergartens crèches	overall: 2 487<br><br>newly infected: 182	n/a	7-17 yo	median: 12 yo	Alpha	**Symptoms**<br>17% fatigue (30/182), 17% sore throat (30/182), 15% headache (27/182), 14% fever (26/182)	**sequencing:** n/r
Somekh et al. [17]	cross-sectional study	Israel	households	alpha infection: 31<br><br>non-VOC infection: 20	n/a	11-17 yo	n/r	Alpha	**Symptoms (alpha vs. non-VOC)**<br>sensory impairment: 6.5% (2/31) vs. 40% (8/20)<br>(RR[3]: 0.16; 95%CI[4]: 0.04–0.6)	**sequencing:** n/r

[1] yo: years old
[2] VOC: Variants Of Concern
[3] RR: Risk Ratio
[4] 95%CI: 95% Confidence Interval

For peer review only

Waltenburg et al.<br>[18]	cohort study	USA[5]	households	n/r	n/r	<5 yo,<br>5-11 yo,<br>12-17 yo	median:<br>12 yo<br>(IQR[6] 7-15)	Alpha	**Symptoms (alpha vs. non-VOC)**<br>constitutional (chills, fever, myalgia):<br>OR[7] 5.5 (95%CI: 1.55-19.20)<br>lower respiratory (e.g. cough):<br>OR 3.52 (95%CI: 1.26-9.86)<br>gastrointestinal (e.g. diarrhoea, pain):<br>OR 3.55 (95%CI: 1.16-10.89)<br><br>**Severity (alpha vs. non-VOC):**<br>Symptom duration (median, days):<br>3 vs. 2, p[8] = 0.64	**vaccination status:**<br>97% unvaccinated (147/151), 3% partially vaccinated (4/151)<br><br>**sequencing:**<br>majority of primary cases sequenced
Nakel<br>et al.<br>[19]	cohort study	Germany	schools<br>kindergartens<br>crèches	infected: 40<br><br>alpha infection: 18<br><br>non-VOC infection: 22	n/a	≤6 yo	n/r	Alpha	**Symptoms (alpha vs. non-VOC)**<br>fever: 38.9% (7/18) vs. 50% (11/22)<br>cough: 44.4% (8/18) vs. 36.4% (8/22)<br>shortness of breath: 11.1% (2/18) vs. 0% (0/22)<br><br>**Severity (alpha vs. non-VOC)**<br>ICU[9] admission: 0 vs. 0	**sequencing:**<br>partially
Somekh<br>et al.<br>[33]	cohort study	Israel	hospital inpatients<br>hospital outpatients	alpha infection:<br>72 796<br><br>non-VOC infection:<br>26 689	n/a	≤9 yo	n/r	Alpha	**Severity (alpha vs. non-VOC)**<br>Hospitalisation: 0.52% (379/72796) vs. 0.98% (261/26689)<br>RR 0.53 (95% CI: 0.46-0.63), p < 0.001<br><br>Severe disease/death: 6.9% (26/379) vs. 6.5% (17/261)<br>RR 0.99 (95% CI: 0.96-1.04)	**sequencing:** n/r
Swann<br>et al.<br>[20]	cohort study	United Kingdom	hospital inpatients	infected:<br>1 617<br><br>alpha infection:<br>952<br><br>non-VOC infection:<br>665	n/a	≤19 yo	median (alpha):<br>6.5 yo<br>(IQR 0.3-14.9)<br><br>median (non-VOC): 4.0 yo<br>(IQR 0.4-13.6)	Alpha	**Symptoms (alpha vs. non-VOC)**<br>fever: 57.1% (544/952) vs. 73.8% (491/665), p<0.001<br><br>**Severity (alpha vs. non-VOC)**<br>Length of hospitalisation: 2 days (IQR 1-4) vs. 2 days (IQR 1-4), p=0.079<br>critical care: 12.0% (109/910) vs. 12.7 (78/616), p=0.989	**sequencing:** n/r

[5] USA: United States of America
[6] IQR: Interquartile Range
[7] OR: Odd Ratio
[8] p: p-value
[9] ICU: Intensive Care Unit

Brookman et al. [34]	cohort study	United Kingdom	hospital outpatients	infected: 80<br><br>alpha infection: 60<br><br>non-VOC infection: 20	n/a	≤18 yo	n/r	Alpha	**Severity (alpha vs. non-VOC)**<br>Critical disease: 3% (2/60) vs. 20% (4/20)<br>Oxygen: 8% (5/60) vs. 35% (7/20)<br>non-invasive ventilation: 3% (2/60) vs. 15% (3/20)<br>invasive ventilation: 2% (1/60) vs. 20% (4/20)	**sequencing:** n/r
Stewart et al. [21]	cohort study	United Kingdom	hospital inpatients	infected: 110<br><br>alpha infection: 67<br>non-VOC infection: 43	n/a	<18 yo	median overall: 10.2 yo (IQR 7.6-12.6)	Alpha	**Symptoms (alpha vs. non-VOC)**<br>PIMS-TS[10]: 67 vs. 43 cases<br>acute kidney injury in PIMS-TS: 28.4% (19/67) vs. 33% (14/43), p=0.85	**sequencing:** n/r

[10] 6PIMS-TS/MIS-C: Paediatric Inflammatory Multisystem Syndrome/Multisystem Inflammatory Syndrome in Children

For peer review only - http://bmjopen.bmj.com/site/about/guidelines.xhtml

Supplementary table 8: Review question 1 - Child/adolescent symptoms/severity of disease when infected with the delta variant (n=13)

Author	Study design	Country	Study setting	Number of study participants <20 yo[1]	Number of adult study partici-pants	Age groups	Mean/ Median age	VOC[2]	Symptoms/Severity	Comments
Li et al. [22]	cohort study	China	hospital inpatients	infected: 77	n/a	<12 yo	median: 9 yo	Delta	**Symptoms**<br>75.3% fever (58/77), 18.2% cough (14/77), 7.8% expectoration (6/77)	**sequencing:** all everyone was hospitalised for isolation
Hao et al. [23]	cohort study	China	hospital inpatients	infected: 66	n/a	≤18 yo	mean (±SD[3]): 10.35±5.86 yo	Delta	**Symptoms**<br>47.0% fever (31/66), 47.0% cough (31/66), 34.8% nasal congestion (23/66), 25.8% sore throat (17/66), 10.6% fatigue (7/66), 4.5% decreased sense of smell (3/66)	**Sequencing:** n/r
Nygaard et al. [48]	cohort study	Denmark	hospital inpatients	infected: 74<br><br>delta infection : 51<br><br>non-VOC infection: 23	n/a	≤17 yo	n/r	Delta	**MIS-C[4] symptoms (delta vs. non-VOC)**<br>hypotension/shock: 51% (26/51) vs. 57% (13/23)<br>cardiac involvement: 90% (46/51) vs. 100% (23/23)<br>gastrointestinal involvement: 98% (50/51) vs. 100% (23/23)<br>respiratory involvement: 12% (6/51) vs. 26% (6/23)<br><br>**MIS-C severity (delta vs. non-VOC)**<br>ICU[5] admission: 55% (28/51) vs. 52% (12/23)<br>vasoactive support: 16% (8/51) vs. 22% (5/23)<br>no need for mechanical ventilation or ECMO[6], no deaths for both delta and non-VOC infection<br><br>(risk of MIS-C comparable with delta and non-VOC infection)	**vaccination status:** none vaccinated<br><br>**sequencing:** majority sequenced

[1] yo: years old
[2] VOC: Variants Of Concern
[3] SD: Standrad Deviation
[4] MIS-C: Multisystem Inflammatory Syndrome in Children
[5] ICU: Intensive Care Unit
[6] ECMO: Extracorporeal Membrane Oxygenation

For peer review only

Murillo-Zamora et al. [37]	cohort study	Mexico	hospital inpatients	infected: 26 961<br><br>person-days: 297 099	n/r	≤19 yo	mean (±SD): 14.4±5.8 yo	Delta	**Severity (delta vs. pre-delta)**<br>pneumonia: incidence rate 30.3/10 000 person-days vs. 9.4/10 000 person-days<br>RR[7] 0.98 (95%CI: 0.97-0.99), p[8]<0.001	**sequencing:** n/r<br>no information on pre-delta strains
Ryu et al. [24]	cohort study	South Korea	population	infected: 235<br><br>delta infection: 92<br><br>pre-delta infection: 143	n/r	<18 yo	mean (delta): 10.2 yo<br><br>mean (pre-delta): 13.8 yo<br>p<0.001	Delta	**Symptoms (delta vs. pre-delta)**<br>rhinorrhoea: 10.5% (15/143) vs. 25% (23/92), p=0.003<br>nasal stuffiness: 15.4% (22/143) vs. 34.8% (32/92), p=0.001<br>sore throat: 12.6% (18/143) vs. 23.9% (22/143), p=0.02<br><br>no difference in prevalence of other COVID symptoms (e.g. fever, cough, pneumonia)	**sequencing:** n/r<br>no information on pre-delta strains
Fisman et al. [38]	cohort study	Canada	population	infected: 20 434<br><br>delta infection: 12 387<br><br>non-VOC infection: 8 047	n/a	≤19 yo	n/r	Delta	**Severity (delta vs. non-VOC)**<br>hospitalization (<10 yo): OR[9] 2.51 (95%CI: 1.23-5.11)<br>hospitalization (10-19 yo): OR 0.78 (95%CI: 0.38-1.91)<br><br>ICU admission (10-19 yo): OR 1.37 (95%CI: 0.11-16.46)	**sequencing:** all sequenced
Shoji et al. [39]	cohort study	Japan	hospital inpatients	infected: 1 299<br><br>delta infection: 349<br><br>pre-delta infection: 950	n/a	<18 yo	median (delta): 7.0 yo (IQR[10]: 2-13)<br><br>median (pre-delta): 10.0 yo (IQR: 4-15)<br>p<0.001	Delta	**Severity (delta vs. pre-delta)**<br>ICU admission: 1.4% (5/349) vs. 0.1% (1/950), p=0.006<br>length of hospitalisation (median, days): 7.0 (IQR 5.0-9.0) vs. 8.0 (IQR 5.0-9.0), p=0.031<br>mechanical ventilation: 0% vs. 0%	**sequencing:** n/r<br>no information on pre-delta strains

[7] RR: Risk Ratio
[8] p: p-value
[9] OR: Odd Ratio
[10] IQR: Interquartile Range

For peer review only

Martin et al. [40]	cohort study	USA[11]	hospital inpatients hospital outpatients	infected: 167 262<br><br>delta infection: 29 191<br><br>pre-delta infection: 138 071	n/a	<19 yo	median (delta): 10.3 yo (IQR 5.3-14.9)<br><br>median (pre-delta): 12.3 yo (IQR 6.2-16.4)	Delta	**Severity (delta vs. pre-delta)**<br>hospitalisation: 6.0% (1738/29191) vs. 6.2% (8507/138071), p=0.18<br>severe disease: 10.3% (179/1738) vs. 14.6% (1242/8507), (OR 0.67, 95%CI: 0.57-0.79), p<0.001<br>severe disease (children classified as non-black, non-white): OR 1.69 (95%CI: 1.02-2.82, p=0.04	**sequencing:** n/r<br>no information on pre-delta strains
Butt et al. [25]	cohort study	Qatar	population	infected: 246<br><br>delta infection: 125<br><br>beta infection: 121	n/a	≤19 yo	n/a	Beta, Delta	**Symptoms (delta vs. beta)**<br>moderate disease: aOR[12] 0.17 (0.06-0.48) vs. 0.35 (0.09-1.39), p=0.54<br>severe disease: aOR 0.08 (0.01-0.48) vs. NA, p>0.99	**sequencing:** all sequenced
Molteni et al. [26]	cohort study	United Kingdom	population	infected: 1 400<br><br>delta infection: 706<br>5-11 yo: 227<br>12-17 yo: 479<br><br>alpha infection: 694<br>5-11 yo: 276<br>12-17 yo: 418	n/r	5-17 yo	median (alpha): 12 yo (IQR 9-15)<br><br>median (delta): 14 yo (IQR 13-16)	Alpha, Delta	**Symptoms (delta vs. alpha)**<br>5-11yo<br>headache: 60.8% (138/227) vs. 39.9% (110/276), OR 2.31 (95%CI: 1.6-3.33), p<0.001<br>fever: 46.3% (105/227) vs. 31.9% (88/276), OR 1.89 (95%CI: 1.31-2.73) (p=0.001)<br>12-17yo<br>headache: 73.7% (353/479) vs. 61.5% (257/418), OR 1.81 (95%CI: 1.36-2.42), p<0.001<br>fever: 43.2% (207/479) vs. 27.0% (113/418), OR 2.05 (95%CI: 1.55-2.72), p<0.001<br>rhinorrhoea: 57.6% (276/479) vs. 47.4% (198/418), OR 1.52 (95%CI: 1.17-1.98), p=0.002<br>sore throat: 60.5% (290/479) vs. 45.7% (191/418), OR 1.87 (95%CI: 1.43-2.45), p<0.001<br><br>**Severity (delta vs. alpha)**<br>symptom burden (5-11 yo, median/days): 4 (IQR 2-7) vs 3 (IQR 2-5)<br>symptom burden (12-17 yo, median/days): 6 (IQR 3-9) vs 5 (IQR 3-8)	**sequencing:** n/r

[11] USA: United States of America
[12] aOR: Adjusted Odd Ratio

For peer review only - http://bmjopen.bmj.com/site/about/guidelines.xhtml

For peer review only

Kildegaard et al. [27]	cohort study	Denmark	Hospital inpatients and outpatients	infected: 74 611<br><br>alpha infection: n/r<br><br>delta infection: n/r	n/a	<18 yo	Median (infected): 8 yo (IQR 1-14)	Alpha, Delta	**Severity (alpha vs. delta)**<br>hospitalization: RR[13] 0.49% (98/20102) (95%CI[14]: 0.4-0.59) vs. 0.48% (18/16694) (95%CI: 0.38-0.6)<br>MIS-C: RR 0.04% (9/20164) (95%CI: 0.02-0.08) vs. 0.04% (5/12778) (95%CI: 0.01-0.09)	**vaccination status:** partially vaccinated<br><br>**sequencing:** n/r
Çelebi et al. [41]	cohort study	Turkey	hospital inpatients hospital outpatients	infected: 680<br><br>alpha infection: 329<br><br>beta/gamma infection: 17<br><br>delta infection: 165<br><br>non-VOC infection: 169	n/a	≤17 yo	median: 9.25 yo	Alpha, Beta, Delta	**Severity (alpha vs. delta vs. beta/gamma vs. non-VOC)**<br>hospitalization: 9.4% (31/329) vs. 19.4% (32/165) vs. 18% (3/17) vs. 10.1% (17/169)<br><br>**Severity (alpha vs. delta)**<br>hospitalization: 9.4% (31/329) vs. 19.4% (32/165), p=0.016<br><br>**Severity (delta vs. non-VOC)**<br>hospitalization: 19.4% (32/165) vs. 10.1% (17/169), p=0.002	**sequencing:** all sequenced, but no differentiation between gamma and beta

[13] RR: Risk Ratio
[14] 95%CI: 95% Confidence Interval

For peer review only - http://bmjopen.bmj.com/site/about/guidelines.xhtml
For peer review only

Edward et al. [28]	cohort study	USA	hospital outpatients	infected: 499<br><br>alpha infection: 96<br><br>gamma infection: 38<br><br>delta infection: 119<br><br>non-VOC infection: 243	n/a	≤18 yo	median (alpha): 9 yo (IQR 2-14)<br><br>median (gamma): 7 yo (IQR 1-14)<br><br>median (delta): 4 yo (IQR 1-11)<br><br>median (non-VOC): 7 yo (IQR 1-12)	Alpha, Delta, Gamma	**Symptoms (alpha vs. gamma vs. delta vs. non-VOC)**<br>symptomatic: 91.6% (88/96) vs. 89.4% (34/38) vs. 89.9% (107/119) vs. 91.7% (223/243), p=0.92<br><br>**Severity - Hospitalization**<br>non-VOC: 3.2% (8/243) reference<br>alpha: 7.2% (7/96): OR 1.9 (95%CI: 0.59-6.3)<br>gamma: 15.7% (6/38): OR 5.9 (95%: 1.6-21.5), p=0.007<br>delta: 6.7% (8/119): OR 2.2 (95%CI: 0.69-7.7)<br><br>**ICU admission**<br>non-VOC: 1.2% (3/243): reference<br>alpha: 1.0% (1/96) :OR 0.47 (95%CI: 0.02-4.0)<br>gamma: 5.2% (2/38): OR 2.6 (95%CI: 0.31-17.5)<br>delta: 2.5% (3/119): OR 1.4 (95%CI: 0.24-9.0)<br><br>**Respiratory support**<br>non-VOC: 1.2% (3/243): reference<br>alpha: 0% : OR 0<br>gamma: 10.5% (4/38) : OR 8.3 (95%CI: 1.5-56.3), p=0.02<br>delta: 2.5% (3/119) : OR 2.2 (95%CI: 0.34-16.9)	**sequencing:** all sequenced

For peer review only

Supplementary table 9: Review question 1 - Child/adolescent symptoms/severity of disease when infected with the omicron variant (n=11)

Author	Study design	Country	Study setting	Number of study participants <20 yo[1]	Number of adult study partici-pants	Age groups	Mean/ Median age	VOC[2]	Symptoms/severity	Comments
Cloete et al. [29]	cohort study	South Africa	hospital inpatients	infected: 139	n/a	≤13 yo	n/r	Omicron	**Symptoms**<br>47% fever, 40% cough, 24% vomiting, 23% difficulty breathing, 20% convulsions<br><br>**Severity**<br>hospitalization: 92% standard ward care, 25% oxygen therapy, 2% high flow oxygen, 6% ventilation	**sequencing:** partially sequenced
Brewster et al. [14]	cohort study	USA[3]	hospital inpatients, hospital outpatients	infected: 75<br><br>omicron: 61<br><br>pre-omicron: 14	n/a	n/r	median (omicron): ED[4]: 2.4 yo (0.8–2.6) hospitalized: 1.6 yo (0.7–1.1)<br><br>median (pre-omicron): ED: 2.6 yo (1.6–3.4) hospitalized: 1.3 yo (1.1–1.5)	Omicron	**Symptoms (omicron vs. pre-omicron):**<br>Croup: 81% (61/75) vs. 19% (14/75)<br><br>Median weekly croup cases (omicron vs. pre-omicron): 11 [IQR[5] 2–17] vs. 0 (IQR 0-0), p[6] < 0.001<br><br>none required invasive ventilation or died (irrespective of variant)	**sequencing:** n/r
Ludvigsson et al. [30]	case series (>1 case)	Sweden	hospital inpatients	infected: 3	n/r	<15 yo	n/r	Omicron	**Symptoms**<br>**Case 1:** repeated convulsions over several hours, hospitalisation: 4 days<br>**Case 2:** status epilepticus, hospitalisation: 2 days<br>**Case 3:** one convulsion (30-60sec), short behavioural change, hospitalisation: 2 days	**sequencing:** n/r<br>14yo: clinical diagnosis

[1] yo: years old
[2] VOC: Variants Of Concern
[3] USA: United States of America
[4] ED: Emergency Department
[5] IQR: Interquartile Range
[6] p: p-value

For peer review only

Butt et al. [31]	cohort study	Qatar	population	Infected: 985<br><br>(both the omicron and delta cohort)	n/r	<18 yo	median (delta): 7 yo (IQR 3-9)<br><br>median (omicron): 6 yo (IQR 3-10)	Delta, Omicron	**Severity (omicron vs. delta)**<br>mild/not hospitalised: 97.8% (963/985) vs. 84.2% (829/985), p<0.001<br>moderate disease: 2.2% (22/985) vs. 15.7% (155/985)<br>critical disease: 0% (0/985) vs. 0.1% (1/985)<br><br>moderate+critical disease: 2.2% (22.985) vs. 15.8% (156/985), p<0.001, aOR[7] 0.12 (95%CI: 0.07-0.18)	**vaccination status:** none vaccinated<br><br>**sequencing:** n/r
Fowlkes et al. [32]	cohort study	USA	population	infected: 252<br><br>omicron infection: 150<br>delta infection: 102	n/r	5-11 yo, 12-15 yo	n/r	Delta, Omicron	**Symptoms (omicron vs. delta)**<br>COVID[8]- symptoms: 48.7% (73/150) vs. 65.7% (67/102); OR[9] 2.0 (95%CI[10]: 1.2-3.45), p=0.008‘<br><br>**Severity (omicron vs. delta)**<br>total days of symptoms: 5.4% vs. 8.0%; OR -3.4 (95%CI: (-)5.7 - (-)1.0), p=0.006<br>hours of missed school: 21.8% vs. 24.1%**;** OR -10.6 (95%CI: (-)18.6 - (-)2.7), p=0.01<br><br>(no differences in febrile symptoms, received medical care, days spent sick in bed)	**vaccination status:** 5-11 yo: 65% (682/1052) double vaccinated, 7% (69/1052) 1 dose, and 29% (301/1052) unvaccinated. 12–15 yo: 68% (212/312) double vaccinated, 5% (15/312) 1 dose, 27% (85/312) unvaccinated<br><br>**sequencing:** n/r
Wang et al. [42]	cohort study	USA	hospital inpatients hospital outpatients	infected: 7 198<br><br>(both the omicron and the delta cohort)	n/r	<5 yo	mean (omicron): 1.49±1.42<br><br>mean (delta): 1.48±1.42	Delta, Omicron	**Severity (omicron vs. delta)**<br>ED visits: 18.83 %(1355/7198) vs. 26.67 (1920/7198); RR[11] 0.71 (95% CI: 0.66-0.75)<br>hospitalisation: 1.04% (75/7198) vs. 3.14 (226/7198); RR 0.33 (95%CI: 0.26-0.43)<br>ICU[12] admission: 0.14% (10/7198) vs. 0.43% (31/7198); RR 0.32 (95%CI: 0.16-0.66)<br>mech. ventilation: 0.33% (24/7198) vs. 1.15% (83/7198); RR 0.29 (95%CI: 0.18-0.46)	**sequencing:** n/r

[7] aOR: Adjusted Odd Ratio
[8] COVID: Coronavirus Disease
[9] OR: Odd Ratio
[10] 95%CI: 95% Confidence Interval
[11] RR: Risk Ratios
[12] ICU: Intensive Care Units

For peer review only

Wang et al. [43]	cohort study	USA	hospital inpatients<br>hospital outpatients	infected ≤4 yo: 11 556 (both the omicron and the delta cohort)<br><br>infected 5-17 yo: 18 715 (both the omicron and the delta cohort)	n/a	≤4 yo, 5-17yo	n/a	Delta, Omicron	**Severity (omicron vs. delta)**<br>≤ 4yo<br>ED visits: 20.41% (2359/11556) vs. 23.96% (2796/1156); RR 0.85 (95%CI: 0.81-0.89)<br>hospitalization: 1.55% (179/11556) vs. 3.0% (347/11556); RR 0.52 (95%CI: 0.43-0.62)<br>ICU admissions: 0.1% (12/11556) vs. 0.42% (49/11556); RR 0.25 (95%CI: 0.13-0.46)<br>mech. ventilation: 0.09% (10/11556) vs. 0.2% (23/11556); RR 0.44 (95%CI: 0.21-0.91)<br><br>5-17yo<br>ED visits: 10.09% (1889/18715) vs. 13.07% (2446/18715); RR 0.77 (95%CI: 0.73-0.82)<br>hospitalization: 0.77% (144/18715) vs. 1.37% (144/18715);<br>RR 0.56 (95%CI: 0.46-0.69)<br>ICU admissions: 0.23% (43/18715) vs. 0.38% (71/18715); RR 0.61 (95%CI: 0.42-0.88)<br>mech. ventilation: 0.05% (10/18715) vs. 0.06 (11/18715); RR 0.91 (95%CI: 0.39-2.14)	**sequencing:** n/r<br>**vaccination status:** n/r
Marks et al. [44]	cohort study	USA	hospital inpatients	infected: 2 100<br><br>omicron infection: 266<br><br>delta infection: 1 834	n/r	≤4 yo, 5-11 yo, 12-17yo	median: 7 yo (IQR 1-14)	Delta, Omicron	**Severity (omicron vs. delta)**<br>ICU admission (≤17 yo): 20.2% (52/266) vs. 27.8% (510/1834)<br>mech. ventilation (≤17 yo): 2.3% (6/266) vs. 6.3% (112/1834)<br><br>hospitalization (≤4yo): 54.2% (142/266) vs. 42.5% (778/1834); RR 5.4 (95%CI: 4.0-7.2)<br>hospitalization (5-11 yo): 16.9% (43/266) vs. 22.5% (417/1834); RR 2.3 (95%CI: 1.5-3.6)<br>hospitalization (12–17 yo): 28.9% (81/266) vs. 34.9% (639/1834); RR 3.5 (95%CI: 2.5-5.0)	**sequencing:** n/r<br>**vaccination status:** n/r
Iuliano et al. [45]	cross-sectional study	USA	hospital inpatients	infected: 824<br><br>omicron infection: 405<br><br>delta infection: 272<br><br>non-VOC infection: 147	n/a	≤17 yo	n/r	Delta, Omicron	**Severity (omicron vs. delta)**<br>ICU admission: 10.4% (42/405) vs. 18.4% (50/272)<br>(ICU admission non-VOC: 17.0% (25/147))	**sequencing:** n/r<br>**vaccination status:** n/r

For peer review only

Shi et al. [46]	cross-sectional study	USA	hospital inpatients	infected: 1 475<br><br>omicron infection: 397<br><br>delta infection: 482<br><br>pre-Delta infection: 596	n/a	5-11 yo	median: 8 yo (IQR 6-10)	Delta, Omicron	**Severity (omicron vs. delta)**<br>ICU admission: 18.9% vs. 26.1%, p=0.05<br>mech. ventilation: 4.6% vs. 6.8%, p=0.28<br>death: 0% vs. 0%<br><br>**Severity (omicron vs. pre-delta)**<br>ICU admission pre-Delta: 18.9% vs. 32.6%, p<0.01<br>mech. ventilation: 4.6% vs. 6.7%, p=0.28<br>death: 0%. vs. 0.7%<br><br>**underlying diseases (omicron vs. delta):**<br>neurologic disorders: 33% vs. 21%, p<0.01<br>obesity: 33% vs. 21%, p = 0.01	**sequencing:** n/r<br>**vaccination status:** n/r
Marks et al. [47]	cohort study	USA	Hospital inpatients	infected: 2 562<br><br>omicron infection: 572<br><br>delta infection: 790<br><br>pre-delta infection: 1 200	n/a	≤4 yo	median: 0.6yo (IQR 0.1–1.0)	Delta, Omicron	**Severity (omicron vs. delta vs. pre-delta):**<br>length of hospitalisation (days, median, IQR): 1.5 (0.5-2.5) vs. 2 (1-3.5) vs. 1.5 (1-3.5), p (omicron vs. pre-delta)= 0.001, p (omicron vs. delta)=0.002<br>ICU admission: 21.0 % vs. 26.7% vs. 24%, p (omicron vs. pre-delta)= 0.19, p (omicron vs. delta)=0.02<br>BiPAP/CPAP[13]: 5.1% vs. 9.1% vs. 5.9%, p (omicron vs. pre-delta)= 0.53, p (omicron vs. delta)=0.008<br>high flow nasal canula: 13.4% vs. 20.4% vs. 8.3%, p (omicron vs. pre-delta)= 0.002, p (omicron vs. delta)=0.002<br><br>No difference in invasive mechanical ventilation and in hospital deaths	**vaccination status:** none vaccinated<br><br>**sequencing:** n/r

[13] BiPAP/CPAP: Bilevel Positive Airway Pressure/ Continuous Positive Airway Pressure

For peer review only - http://bmjopen.bmj.com/site/about/guidelines.xhtml
For peer review only

Supplementary table 10: Review question 1 - Child/adolescent symptoms/severity of disease when infected with the gamma variant (n=2)

Author	Study design	Country	Study setting	Number of study participants <20 yo[1]	Number of adult study participants	Age groups	Mean/Median age	VOC[2]	Symptoms/Severity	Comments
Oliveira et al. [35]	cohort study	Brazil	hospital inpatients	infected: 21 591<br><br>gamma infection: 10 017<br><br>non-VOC infection: 11 574	n/r	<20 yo	mean (gamma): 4.0 yo (IQR[3] 0.6 - 14.7)<br><br>mean (non-VOC): 5.2 yo (IQR 1.0 - 14.4)	Gamma	**Severity (gamma vs. non-VOC)**<br>hypoxemia: 52.5% vs. 41.1%, p[4]<0.0001<br>ICU[5] admissions: 28.3% (2498/8830) vs. 24.9% (2745/11037), p<0.0001<br>invasive vent. support: 10.1% (974/9659) vs. 10.8% (1156/10733) p<0.0001	**sequencing:** n/r

For peer review only

[1] yo: years old
[2] VOC: Variant Of Concern
[3] IQR: Interquartile Range
[4] p: p-value
[5] ICU: Intensive Care Unit

Edward et al. [36]	cohort study	USA[6]	hospital outpatients	infected: 499<br><br>alpha infection: 96<br><br>gamma infection: 38<br><br>delta infection: 119<br><br>non-VOC infection: 243	n/a	≤ 18 yo	median (alpha): 9 yo (IQR 2-14)<br><br>median (gamma): 7 yo (IQR 1-14)<br><br>median (delta): 4 yo (IQR 1-11)<br><br>median (non-VOC): 7 yo (IQR 1-12)	Alpha, Delta, Gamma	**Symptoms**<br>**(alpha vs. gamma vs. delta vs. non-VOC)**<br>symptomatic: 91.6% (88/96) vs. 89.4% (34/38) vs. 89.9% (107/119) vs. 91.7% (223/243), p=0.92<br><br>**Severity - Hospitalization**<br>non-VOC: 3.2% (8/243) reference<br>alpha: 7.2% (7/96): OR[7] 1.9 (95%CI[8]: 0.59-6.3)<br>gamma: 15.7% (6/38):<br>OR 5.9 (95%: 1.6-21.5), p=0.007<br>delta: 6.7% (8/119):<br>OR 2.2 (95%CI: 0.69-7.7)<br><br>**ICU admission**<br>non-VOC: 1.2% (3/243): reference<br>alpha: 1.0% (1/96):<br>OR 0.47 (95%CI: 0.02-4.0)<br>gamma: 5.2% (2/38):<br>OR 2.6 (95%CI: 0.31-17.5)<br>delta: 2.5% (3/119):<br>OR 1.4 (95%CI: 0.24-9.0)<br><br>**Respiratory support**<br>non-VOC: 1.2% (3/243): reference<br>alpha: 0%: OR 0<br>gamma: 10.5% (4/38):<br>OR 8.3 (95%CI: 1.5-56.3), p=0.02<br>delta: 2.5% (3/119)<br>OR 2.2 (95%CI: 0.34-16.9)	**sequencing:** all sequenced

For peer review only

[6] USA: United States of America
[7] OR: Odds Ratio
[8] 95%CI: 95% Confidence Interval

Supplementary table 11: Review question 3 - Child/Adolescent risk of becoming infected with a SARS-CoV-2 variant of concern (n=15)

Author	Study design	Country	Study setting	Number of contacts <20 yo[1]	Number of adult contacts	Age groups	Mean/ Median age	VOC[2]	Risk of infection compared to non-VOCs/other VOCs	Risk of infection compared to adults	Comments
Schenk et al. [53]	cohort study	Germany	schools kindergartens crèches	non-VOC: 577<br><br>alpha: 756	non-VOC: 334<br>alpha: 226	≤7 yo, adults	median: 4yo	Alpha	**Risk of infection (alpha vs. non-VOC)**<br>0% (0/756) vs. 1.2% (7/577)	**Risk of infection (children vs. adults)**<br>0% (0/756) vs. 0% (0/226)	**vaccination status:** n/r<br>**sequencing:** n/r
Neuberger et al. [54]	cross-sectional study	Germany	schools kindergartens crèches	n/r<br>8500 early childhood education and care centres	n/r	≤2 yo, 4-6 yo, ≥7 yo, adults	n/r	Alpha	n/a	**Risk of infection (children vs. adults)**<br>higher absolute case number, but overall rate of infection still higher in adults	**vaccination status:** n/r<br>**sequencing:** n/r
Nakel et al. [19]	cohort study	Germany	schools kindergartens crèches	113	31	≤6 yo	n/r	Alpha	**Risk of infection (alpha vs. non-VOC)**<br>15.9% (18/113) vs. 64.7% (22/34)	**Risk of infection (children vs. adults)**<br>15.9% (18/113) vs. 35.5% (11/31)	**vaccination status:** n/r<br>**Sequencing:** few cases sequenced, presence of variant assumed for the rest based on date of sampling and national prevalence
Roberts et al. [15]	case-control study	United Kingdom	schools kindergartens crèches	83	34	≤4 yo, adults	n/r	Alpha	n/a	**Risk of infection AR[3] (children vs. adults)**<br>25.3% (21/83) vs. 70.6% (24/34)<br><br>**AR (children):**<br>- 0-1yo: 44%<br>- 1-2 yo: 31%<br>- 2-3yo: 31%<br>- 3-4 yo: 4%	**vaccination status:** n/r<br>**sequencing:** 1 case sequenced, presence of variant assumed for the rest based on date of sampling and national prevalence

[1] yo: years old
[2] VOC: Variant Of Concern
[3] AR : Attack Rate

For peer review only - http://bmjopen.bmj.com/site/about/guidelines.xhtml

Loenenbach et al. [55]	cohort study	Germany	schools kindergartens crèches	133	60	≤6 yo, adults	n/r	Alpha	n/a	**Risk of infection SAR[4] overall (children vs. adults)** 21.1% (28/133) vs. 31.7% (19/60) **SAR per daycare centre (children vs. adults)** 1. 31% (15/49) vs. 53% (10/19) 2. 27% (7/26) vs. 28% (5/18) 3. 17% (6/36) vs. 17% (4/23)	**vaccination status:** n/r **sequencing:** n/r
Lorthe et al. [56]	cohort study	Switzerland	schools kindergartens crèches	70	9	4-6 yo, adults	n/r	Alpha	n/a	**Risk of infection AR (children vs. adults)** 27.4% (22/70) vs. 22.2% (2/9) **AR (children by classes)** 1. 62.9% (13/21) 2. 10.5% (2/19) 3. 16.7% (3/18) 4. 13.3% (2/15)	**vaccination status:** n/r **sequencing:** all cases
Abu-Raddad et al. [57]	cohort study	Qatar	population	55 638 <10 yo: 28 791 10-19 yo: 26 847	30-39yo: 81 760	<10 yo, 10-19 yo, adults	n/r	Alpha	n/a	**Risk of infection (children <10 yo vs. adults)** 1.7% (502/28791) vs. 3.6% (2966/81760) OR[5] 0.35 (95%CI[6]: 0.32–0.39) **Risk of infection (adolescents 10-19 yo vs. adults)** 3.6% (965/26847) vs. 3.6% (2966/81760)	**vaccination status:** n/r **sequencing:** 85% of all cases sequenced

[4] SAR: Secondary Attack Rate
[5] OR: Odd Ratio
[6] 95%CI: 95% Confidence Interval

For peer review only

Somekh et al. [33]	cohort study	Israel	hospital inpatients hospital outpatients	72 426<br><br>Alpha: 50 811<br><br>non-VOC: 21 615	n/r	<10 yo	n/r	Alpha	**Risk of infection (alpha vs. non-VOC)** slope of weekly adjusted incidence: 84.4 (95%CI: 71.1- 97.7) vs 39.1 (95%CI:23.9-54.3)	n/a	**sequencing:** n/r
Somekh et al. [17]	cross-sectional study	Israel	households	Alpha: 77<br><br>non-VOC: 58	n/r	≤5 yo, 6-17 yo	n/r	Alpha	**Risk of infection (alpha vs. non-VOC)** ≤5 yo: 72% (18/25) vs. 11.1% (2/18) RR[7] 7.8 (95%CI: 2.1–29.0)<br><br>6-17 yo: 75% (39/52) vs. 32.5% (13/40) RR 2.5 (95%CI: 1.6–4.0)	n/a	**sequencing:** n/r
Waltenburg et al. [18]	cohort study	USA[8]	households	Alpha : 61<br><br>non-VOC : 39	n/r	≤11 yo, 12-17 yo	Median : 12 yo (IQR[9] 7-15)	Alpha	**Risk of infection (alpha vs. non-VOC)** 64% (39/61) (95%CI: 51-76) vs. 56% (22/39) (95%CI: 40-72); OR 1.08 (95% CI: 0.40- 2.98)<br><br>**(≤11 yo vs. 12-17yo)** 78% (21/27) (95%CI: 58-91) vs. 53% (18/34) (95%CI: 35-70); OR 1.37 (95%CI:0.61-3.04)	n/a	**vaccination status:** 97% unvaccinated (147/151), 3% partially vaccinated (4/151) **sequencing:** majority of primary cases sequenced
Clifford et al. [58]	cohort study	United Kingdom	households	67	406	<18 yo, adults	n/r	Delta	n/a	**Risk of infection (children vs. adults)** RR 0.84 (95%CI: 0.66-0.99)<br><br>(risk of being infected by non-elderly adults)	**vaccination status:** n/r **sequencing:** 63.1% of cases sequenced, rest was assumed based on date of sampling and national prevalence

[7] RR: Risk Ratio
[8] USA: United States of America
[9] IQR: Interquartile Range

For peer review only - http://bmjopen.bmj.com/site/about/guidelines.xhtml

For peer review only

Ng et al. [50]	cohort study	Singapore	households	1 136	7223	≤11 yo, 12-17 yo, adults	median : 36 yo (IQR 26-51)	Delta	n/a	**Risk of infection SAR (≤11 yo vs. 18-29 yo)** 22.9% (173/757) vs. 12.0% (212/1765) ; aOR[10] 1.43 (95%CI: 1.07-1.93), p=0.017<br><br>**SAR (12-17 yo vs. 18-29 yo)** 12.9% (49/379) vs. 12.0% (212/1765) ; aOR 0.97 (95%CI: 0.66-1.42), p[11]=0.87	**vaccination status:** >12 yo partially vaccinated (results adjusted for vaccination status) **sequencing:** partially sequenced
Dougherty et al. [51]	cohort study	USA	camps, sport clubs	122	11	<12 yo, 12-19 yo, adults	median: 14 yo (IQR 5-58)	Delta	n/a	**Risk of infection AR (children vs. adults)** 18.9% (23/122) vs. 27.3% (3/11)	**vaccination status:** majority unvaccinated/partially vaccinated **sequencing:** 21 sequenced, rest was assumed based on date of sampling and national prevalence
Singana-yagam et al. [52]	cohort study	United Kingdom	community	30	198	5-18 yo, adults	median: 41 yo (IQR 28-49)	Delta	n/a	**Risk of infection SAR (children vs. adults)** 40% (12/30) vs. 20.1% (41/198)	**vaccination status:** 70% were fully vaccinated, 23.7% were partially vaccinated, 23% were not vaccinated **sequencing:** partially sequenced, rest was assumed based on date of sampling and national prevalence

[10] aOR: adjusted Odd Ratio
[11] p: p-value

For peer review only

Lorthe et al.<br>[59]	Cohort study	Switzerland	schools kindergartens crèches	59	10	3-7 yo, adults	n/r	Omicron		**Risk of infection AR (children vs. adults)**<br>44.1% (26/59) vs. 50% (5/10)<br><br>**AR (children by classes)**<br>1. 33.3% (4/12)<br>2. 15.4% (2/13)<br>3. 56.3% (9/16)<br>4. 61.1% (11/18)	**vaccination status:** n/r<br>**sequencing:** none

For peer review only

Supplementary table 12: Review question 4 - Risk of child/adolescent transmission when infected with a SARS-CoV-2 variant of concern (n=15)

Author	Study design	Country	Study setting	Number of index cases <20 yo[1]	Number of adult index cases	number of contacts of children/ adolescents	number of contacts of adults	Age groups	Mean/ Median age	VOC[2]	Risk of transmission compared to non-VOCs/other VOCs	Risk of transmission compared to adults	Comments
Lindstrom et al. [66]	cohort study	Norway	population	42<br><br>alpha: 14<br><br>non-VOC: 28	373<br><br>alpha: 131<br><br>non-VOC: 242	mean (SD[3]): 2.79 (1.63)	40-59yo: 1.72 (1.87)<br><br>20-39yo: 1.03 (1.36)<br><br>>60yo: 1.25 (1.65)	<20 yo, adults	n/r	Alpha	n/a	**Transmission risk SAR[4] (<20 yo vs. 40-59 yo)** ref. vs. 1.97 (1.08-3.87)<br><br>no significant difference to adults 20-39 yo, >60yo	**vaccination status:** n/r **sequencing:** primary cases sequenced, variants in contacts assumed based on date of sampling and national prevalence
Lyngse et al. [60]	cohort study	Denmark	households	145<br><br><10 yo: 54<br><br>10-19 yo: 91	663	383<br><br><10yo: 141<br><br>10-19yo: 242	1 336	≤10 yo, 10-20 yo, adults	n/r	Alpha	n/a	**Transmission risk AR[5] (≤20 yo vs. adults)** 32.4% (124/383) vs. 39.8% (532/1336)<br><br>**AR (≤10 yo vs. 10-19 yo vs. adults)** 45% (64/141) vs. 25% (60/242) vs. 39.8% (532/1336)	**vaccination status:** n/r **sequencing:** 75% sequenced, rest was assumed based on date of sampling and national prevalence

[1] yo: years old
[2] VOC: Variant Of Concern
[3] SD: Standard Deviation
[4] SAR: secondary attack rate
[5] AR: attack rate

For peer review only - http://bmjopen.bmj.com/site/about/guidelines.xhtml

Chudasama et al. [61]	cohort study	United Kingdom	households	6 011<br><br><10 yo: 1 289<br><br>10-19 yo: 4 722	57 382	n/r	n/r	<10 yo, 10-19 yo, adults	n/r	Alpha	n/a	**Transmission risk OR[6] (10-19 yo vs. <10 yo)**<br>OR 0.83 (95%CI[7]: 0.69-0.99) (<10 yo: reference)<br><br>no difference in transmission risk compared to index cases aged 30-70 yo	**vaccination status:** n/r<br>**sequencing:** all index cases sequenced, no information on contacts
Julin et al. [67]	cohort study	Norway	households	3<br><br>alpha: 2<br><br>non-VOC: 1	55<br><br>alpha: 16<br><br>non-VOC: 39	55<br><br>alpha: 16<br><br>non-VOC: 39	68<br><br>alpha: 20<br><br>non-VOC: 48	<20 yo, adults	median: 31 yo (IQR[8] 2-73)	Alpha	n/a	**Transmission risk (irrespective of VOC)**<br>**Viral loads (mean, <20 yo vs. adults)**<br>2.09 log10 copies/ul RNA[9] vs. 2.98 log10 copies/ul RNA<br><br>**Length of testing positive (mean days, <20 yo vs. adults)**<br>11.3 (95%CI: 7.6–15.1) vs 16.4 (95%CI: 13.5–19.3), p[10] = 0.03	**vaccination status:** none vaccinated<br>**sequencing:** all sequenced

[6] OR: Odd Ratio
[7] 95%CI: 95% Confidence Interval
[8] IQR: Interquartile Range
[9] RNA: Ribonucleic acid
[10] p: p-value

For peer review only

Loenenbach et al. [55]	cohort study	Germany	schools kindergartens crèches households	22	16	59	33	≤6 yo, adults	n/r	Alpha	n/a	**Transmission risk SAR**[11] **(≤6 yo vs. adults)** 39% (95%CI: 28-52) vs. 33% (95%CI: 20-50) RR[12] 1.17 (95%CI: 0.66-2.09)	**vaccination status:** n/r **sequencing:** n/r
Lorthe et al. [56]	cohort study	Switzerland	schools kindergartens crèches households	n/r	n/r	24	2	≤6 yo, adults	n/r	Alpha	n/a	**Transmission risk SAR (≤6 yo vs. adults)** 12.5% (3/24) vs. 50% (1/2)	**vaccination status:** n/r **sequencing:** all cases
Loss et al. [62]	case-control study	Germany	schools kindergartens crèches households	15<br>alpha: 9<br>non-VOC: 6	9<br>alpha: 4<br>non-VOC: 5	172<br>alpha: 114<br>non-VOC: 58	38	≤6 yo, adults	n/r	Alpha	**Transmission risk AR**[13] **(alpha vs. non-VOC)** 16.7% (19/114) vs. 8.6% (5/58)	**Transmission risk AR (≤6 yo vs. adults)** 16.7% (19/114) vs. 10.5% (4/38)	**vaccination status:** n/r **sequencing:** majority sequenced, rest was assumed based on date of sampling and national prevalence
Buchan et al. [63]	cohort study	Canada	households	715<br><10 yo alpha: 55 non-VOC: 195<br>10-19 yo alpha: 114 non-VOC: 351	4 902<br>alpha: 1 149 non-VOC: 3 753	n/r	n/r	<10 yo, 10-19 yo, adults	n/r	Alpha	**Transmission risk SAR (alpha vs. non-VOC)** aRR[14] (<10y o): 1.47 (95%CI: 0.81-2.65) aRR (10-19 yo): 1.30 (95%CI: 0.80-2.10)	n/a	**sequencing:** all cases either sequenced or screened positive for N501Y (marker for alpha variant) by PCR[15]

[11] SAR: Secondary Attack Rate
[12] RR: Risk Ratio
[13] AR: Attack Rate
[14] aRR: Adjusted Risk Ratio
[15] PCR: Polymerase Chain Reaction

For peer review only - http://bmjopen.bmj.com/site/about/guidelines.xhtml

Waltenburg et al. [18]	cohort study	USA[16]	households	36<br><br>alpha: 21<br><br>non-VOC: 10	n/a	111<br><br>alpha : 61<br><br>non-VOC : 39	n/a	<5 yo<br>5-11 yo<br>12-17 yo	median: 12 yo (IQR 7-15)	Alpha	**Transmission risk**<br>**SAR (alpha vs. non-VOC)**<br>55% (24/44) (95%CI: 39-70) vs. 46% (12/26) (95%CI: 27-67); OR 1.52 (95%CI: 0.51-4.53)<br><br>**SAR – alpha (≤11 yo vs. 12-17 yo)**<br>65% (11/17) (95%CI: 38-68) vs. 48% (13/27) (95%CI: 29-68); OR 1.63 (95%CI: 0.4-6.7)	n/a	**vaccination status:** 97% unvaccinated (147/151), 3% partially vaccinated (4/151) (2 primary cases and 2 secondary cases)<br><br>**sequencing:** majority of primary cases sequenced
Ratman et al. [68]	modeling study	USA	population	n/a	n/a	n/a	n/a	<10 yo,<br>10-19 yo	n/r	Alpha	**Transmission risk**<br>**R[17] (alpha vs. non-VOC)**<br><10yo: <1 vs. <<1<br>10-19 yo: ~1 vs. <<1	n/a	n/a
Somekh et al. [33]	cohort study	Israel	hospital inpatients hospital outpatients	72 426<br><br>alpha: 50 811<br><br>non-VOC: 21 615<br><br>(overall numbers and thus potential index cases)	n/r	alpha: 313 871<br><br>non-VOC: 156 521	n/r	<10 yo	n/r	Alpha	**Transmission risk**<br>**AR (alpha vs. non-VOC)**<br>15.7% (49257/313871) vs. 7.5% (11770/156521)<br>RR: 2.24 (95%CI, 2.20-2.29), p < 0.001	n/a	**sequencing:** n/r

[16] USA: United States of America
[17] R: reproduction number

For peer review only - http://bmjopen.bmj.com/site/about/guidelines.xhtml

Allen et al. [64]	case-control study	United Kingdom	households	6 130<br><br>alpha: n/r<br><br>delta: n/r	17 928	n/r	n/r	<10 yo, 10-19 yo, adults	n/r	Alpha, Delta	n/a	**Trasmission risk aOR[18] (delta vs. alpha/ref. adults)**<br><10 yo: 0.98 (95%CI: 0.85-1.14)<br>10-19 yo: 0.71 (95%CI: 0.64-0.79)<br>(reference value 1.00 for 30-39 yo)	**vaccination status:** reported (but not in subanalysis comparing transmission risk in age groups)<br><br>**sequencing:** all index cases sequenced, nationwide 50% of all cases were sequenced at time of study
Trobajo-Sanmartin et al. [65]	cohort study	Spain	population	n/r	n/a	All contacts of <12 yo : 288<br><br>Alpha : 107<br><br>Delta : 181	n/a	<12yo, 12-17 yo, adults	n/r	Alpha, Delta	**Transmission risk SAR (alpha vs. delta)**<br><12 yo : 33% (35/107) vs. 23% (41/181); aRR 1 vs. 0.58 (95%CI: 0.28-1.23), p=0.155<br><br>higher risk of transmission for 12-39yo		**vaccination status:** none vaccinated<br><br>**sequencing:** none

[18] aOR: Adjusted Odd Ratio

For peer review only - http://bmjopen.bmj.com/site/about/guidelines.xhtml
For peer review only

Ng et al. [50]	cohort study	Singapore	households	n/r	n/r	472	18-29 yo : 1 467	≤11 yo, 12-17 yo, adults	median (contacts): 36 yo (IQR 26-51)	Delta	n/a	**Transmission risk SAR (≤11 yo vs. 18-29 yo)** 25.2% (88/349) vs. 9.4% (138/1457); aOR: 2.37 (95%CI:1.57-3.60), p<0.0001<br><br>**SAR (12-17 yo vs. 18-29 yo)** 17.9% (22/123) vs. 9.4% (138/1457) ; aOR 1.81 (95%CI: 0.91-3.6), p=0.089	**vaccination status:** >12 yo partially vaccinated (results adjusted for vaccination status)<br><br>**sequencing:** partially sequenced
Lorthe et al. [59]	cohort study	Switzerland	schools kindergartens crèches, households	n/r	n/a	52	n/a	3-7 yo, adults	n/r	Omicron	**Transmission risk SAR** 48% (25/52)	n/a	**vaccination status:** n/r<br><br>**sequencing:** none

For peer review only
